# Fascin-1 Promotes Cell Metastasis through Epithelial–Mesenchymal Transition in Canine Mammary Tumor Cell Lines

**DOI:** 10.3390/vetsci11060238

**Published:** 2024-05-25

**Authors:** Xin Wang, Ye Zhou, Linhao Wang, Abdul Haseeb, Hongquan Li, Xiaozhong Zheng, Jianhua Guo, Xiaoliang Cheng, Wei Yin, Na Sun, Panpan Sun, Zhenbiao Zhang, Huizhen Yang, Kuohai Fan

**Affiliations:** 1 Shanxi Key Laboratory for Modernization of TCVM, College of Veterinary Medicine, Shanxi Agricultural University, Jinzhong 030801, China; s20212386@stu.sxau.edu.cn (X.W.);; 2 Medical Research Council (MRC) Centre for Inflammation Research, Queen’s Medical Research Institute, The University of Edinburgh, Edinburgh EH16 4TJ, UK; 3 Department of Veterinary Pathobiology, Schubot Exotic Bird Health Center, Texas A&M University, College Station, TX 77843, USA

**Keywords:** dog cancer, cytoskeleton, metastasis, epithelial–mesenchymal transition, proteomics

## Abstract

**Simple Summary:**

Canine mammary tumors are the most common tumor in female dogs. In this study, we obtained a metastatic key protein by comparing the proteomics data of in situ tumor and metastatic cell lines from the same individual. Our results not only help to compare the difference in metastasis processes between canine mammary tumors and human breast cancer but also provide a theoretical basis for the prevention of tumor metastasis and the improvement of prognosis in dogs and humans.

**Abstract:**

Canine mammary tumors (CMTs) are the most common type of tumor in female dogs. In this study, we obtained a metastatic key protein, Fascin-1, by comparing the proteomics data of in situ tumor and metastatic cell lines from the same individual. However, the role of Fascin-1 in the CMT cell line is still unclear. Firstly, proteomics was used to analyze the differential expression of Fascin-1 between the CMT cell lines CHMm and CHMp. Then, the overexpression (CHMm-OE and CHMp-OE) and knockdown (CHMm-KD and CHMp-KD) cell lines were established by lentivirus transduction. Finally, the differentially expressed proteins (DEPs) in CHMm and CHMm-OE cells were identified through proteomics. The results showed that the CHMm cells isolated from CMT abdominal metastases exhibited minimal expression of Fascin-1. The migration, adhesion, and invasion ability of CHMm-OE and CHMp-OE cells increased, while the migration, adhesion, and invasion ability of CHMm-KD and CHMp-KD cells decreased. The overexpression of Fascin-1 can upregulate the Tetraspanin 4 (TSPAN4) protein in CHMm cells and increase the number of migrations. In conclusion, re-expressed Fascin-1 could promote cell EMT and increase lamellipodia formation, resulting in the enhancement of CHMm cell migration, adhesion, and invasion in vitro. This may be beneficial to improve female dogs’ prognosis of CMT.

## 1. Introduction

The incidence of tumors in pet dogs has increased due to the increasing population and longer lifespans [1]. Canine mammary tumors (CMTs) are the most common in female dogs [2,3], with over 50% of cases being malignant [4] and often metastatic [5]. Additionally, its occurrence and development are associated with dog breed, age, gender, and neutering status [6,7,8,9]. Notably, CMTs and human breast cancer (HBC) share a significant number of similarities in pathogenic factors, histological characteristics, molecular phenotypes, biological behaviors, and modes of metastasis [10,11], such as acquired chemotherapeutic resistance [12,13]. Furthermore, CMTs have been suggested as a model to study the pathogenesis and prognosis of HBC [14,15,16].

Fascin-1 is an actin-binding protein (ABP) in membrane folds, microspikes, and stress fibers [17]. It is a unique ABP found along the entire length of the filopodia [18]. Fascin-1 was first identified in sea urchins [19] and subsequently in HeLa cell lines [20]. Various human cancer studies have shown that Fascin-1 promotes [21,22,23,24] or inhibits [25,26] cell migration and invasion when overexpressed or knocked down in vitro, and it is related to the survival time in vivo [27]. Mechanically, a high expression of Fascin-1 is correlated with the process of epithelial–mesenchymal transition (EMT) in HBC cells and endows them with the ability to migrate and invade [24,25]. Simultaneously, the proliferation and EMT of epithelial cells in CMTs are more frequent than in HBC [28], leading cells to lose the original immunophenotype and complicating immunolabeling [29].

Metastatic tumor cells migrate in vivo through the dense extracellular matrix (ECM) by the highly dynamic filopodia formed by crosslinking Fascin-1 with F-actin, along with actin filament self-organization and membrane remodeling [30,31,32]. Filopodia formation has been shown to provide resistance to rigidity from the matrix and other cells, sense the environment around the cell, provide forward thrust for cell migration [33,34,35,36,37], and promote tumor cells’ survival at the metastasis site [38].

G-actin drives the growth of filopodia at the tip, and it is elongated in the direction away from the cell body by the synergism of formins and VASP [39,40,41,42], promoting the establishment of lamellipodia and cell adhesion [34,36]. In this process, Fascin-1 is very important for the growth of filopodia and the establishment of lamellipodia. Therefore, it can be assumed that the expression level of Fascin-1 in tumor cells should be higher than that before metastasis. Once the cells reach secondary tissues and organs, Fascin-1 expression is downregulated through a regulatory process to achieve settlement [43]. Therefore, tumor metastasis, from the detachment of cancer cells from the primary tumor tissue to the invasion of the bloodstream and lymphatic vessels and the settlement in secondary tissues or organs, is closely related to the expression of the Fascin-1 protein [44,45]. Furthermore, regulating Fascin-1 expression can affect tumor cell metastasis by altering the number and strength of cell filopodia [42,46,47,48].

Moreover, the role of Fascin-1 in tumor occurrence [49], colonization [50], resistance to anoikis and chemotherapy, and cancer stemness [51] has been gradually defined. Thus, Fascin-1 has been identified as one of the biomarkers for the clinical diagnosis and prognostication of human tumors [50].

Although the role of Fascin-1 in human tumor development and metastasis is well established, whether Fascin-1 has the same effect on canine tumors remains to be determined. Therefore, lentiviral transduction was used to study the expression of Fascin-1 protein in the CMT cell line to study the function of Fascin-1 in CMT. This project aimed to study the effect and mechanism of Fascin-1 on CMT cell migration, adhesion, and invasion and provide theoretical foundations for CMT treatment with Fascin-1 as a potential therapeutic target.

## 2. Materials and Methods

### 2.1. Cell Culture

All cells were maintained in Dulbecco’s modified Eagle’s medium with high glucose (DMEM, Gibco, Waltham, MA, USA) supplemented with 10% fetal bovine serum (FBS) and 1% antibiotic (penicillin and streptomycin) at 37 °C in a 5% CO_2_ incubator. Cells in the logarithmic growth phase were harvested for further analysis and digested when they were in the logarithmic growth phase for all experiments.

CMT cell lines, CHMm and CHMp, have already been maintained in our laboratory. In the original research, CHMm and CHMp cell lines were originated from a 12-year-old mixed-breed dog suffering from grade IV clinical stage. The TNM grade of the cancer in situ was T4N1(+) M1, and CHMm was isolated from a pleural effusion in this dog [52]. Thus, the HEK-293T cell line was purchased from the Cell Resource Center of Peking Union Medical College (Beijing).

### 2.2. Construction of Fascin-1 Overexpression and Knockdown Cell Line

The Fascin-1 CDS region (Sangon, Shanghai, China) and gene interference target sequence (GenePharma, Shanghai, China) were synthesized. The Fascin-1 CDS region fragment was then ligated to pLVX-IRES-neo (TaKaRa, San Jose, CA, USA) and the interference target sequence (shRNA) was then ligated to pLVX-U6-LUC&Puro. All ligated products were transformed into DH5α (Sangon, Shanghai, China).

Bacterial clones with pLVX-IRES-neo plasmid containing the Fascin-1 gene insert and pLVX-U6-LUC&Puro containing shRNA were selected and cultured at 37 °C with shaking at 225 r/min for 16 h. Endotoxin-free plasmids were extracted (OMEGA, Norcross, GA, USA), for subsequent cell transfection. The lentivirus was packaged in the HEK-293T cell line using the Lenti-X Bicistronic Expression System (TaKaRa, San Jose, CA, USA).

CMT cells were seeded at 2 × 10^5^ cells/well in a six-well plate. An appropriate amount of virus supernatant and Polybrene were added once the cells were adhered. After 24 h of transduction, the cells were passaged normally in a complete medium. After 48 h, the transduced cells were inoculated at a density of 20% in a 10 cm cell culture dish. G418 screened the overexpressed cells, and Puro screened the knockdown cells. While all cells in the control dish died, the remaining dish was cultured in a complete medium and passaged normally or preserved by freezing. Changes in the Fascin-1 gene and protein expression were identified in the transfected cell line. CMT cells transfected with the empty plasmids (pLVX-IRES-neo and pLVX-U6-LUC&Puro) lentivirus served as negative control and were named CHMm-Nt and CHMp-Nt, respectively. Furthermore, CMT cells transfected with the lentivirus with pLVX-IRES-neo/Fascin-1 were named CHMm-OE. Following transfection of the lentivirus with PLVX-U6-1140/1236/1355-LUC&Puro, the cell lines with the best knockdown rate were named CHMm-KD and CHMp-KD.

### 2.3. Cell Viability Assay

Cells were inoculated into a 96-well plate with an initial cell density of 5 × 10^3^ cells/well. Every 24 h, CCK-8 (BOSTER, Wuhan, China) was added to the cells. The plate was further incubated at 37 °C for 30 min, and then the absorbance at 450 nm (with a reference wavelength of 600 nm) was measured using Infinite^®^ E Plex (Tecan, Shanghai, China).

### 2.4. Cell Migration Assay

Cells were seeded at 4 × 10^5^ cells/well in a six-well plate. When the cell density reached 70%, the medium was replaced with a serum-free medium, and the cells were starved for 16 h. Following starvation, a line was scratched in the well using a 200 μL pipette tip, and the cells were washed with PBS to remove floating cells. Subsequently, 50 μM NP-G2-044 was added (IC_90_) to the medium containing 2% FBS for the NP-G2-044 group, and an equal amount of DMSO was added for the DMSO group. After 3 h of incubation, the cells were photographed using an Olympus microscope. After a further 24 h of incubation, the cells were washed and photographed. All pictures were analyzed using ImageJ software (v. 1.53). The percentage of wound healing was calculated as (0 h wound area—24 h wound area)/0 h wound area.

### 2.5. Cell Adhesion Assay

Matrix gel (Corning, Shanghai, China) was coated on 96-well plates at a dilution of 1:8 with DMEM medium and incubated overnight in the cell incubator. Three hours before harvesting the cells, 50 μM NP-G2-044 was added to the cells in the NP-G2-044 group, and an equal amount of DMSO was added to the cells in the DMSO group. Cells were then resuspended in a medium containing 2% FBS, seeded at a concentration of 2 × 10^4^ cells/well, and incubated for 6 h for adhesion. Cells were washed with PBS to remove un-adhered cells, and a blank medium with 10% CCK-8 was added to each well and incubated at 37 °C for 1 h. The absorbance at 450 nm (with a reference wavelength of 600 nm) was measured using a microplate reader.

### 2.6. Cell Invasion Assay

Matrix gel was diluted 1:8 with blank medium and coated onto transwell upper chambers overnight at 37 °C. NP-G2-044 and DMSO were added in the same way as in the cell adhesion assay. The lower chamber was filled with 500 μL medium containing 20% FBS. The transwell was gently inserted into the plate well. Cells were resuspended in DMEM medium and seeded onto the upper chamber with 2 × 10^4^ cells, and the chambers were placed back into the 24-well plates for further incubation.

After 24 h, the upper chamber cells were gently wiped off with a sterile cotton swab, fixed with 4% paraformaldehyde for 15 min, and stained with 0.1% crystal violet for 30 min. After washing with PBS, the membrane was photographed under a microscope. Furthermore, the cell invasion ability was evaluated based on the number of cells that passed through the membrane.

### 2.7. Immunocytochemistry (IHC) and Immunofluorescence Assay (IFA)

Cells (1 × 10^4^ cells) were cultured in a 30 mm glass-bottom dish for 24 h, fixed with 4% paraformaldehyde, blocked with 5% BSA for 1 h, and then incubated overnight with primary antibodies. Cells were washed thrice with PBST and probed with fluorescent-con jugated secondary antibodies (1:100) for 1 h. F-actin was subsequently stained with AF^®^ 680 Phalloidin (Invitrogen, Waltham, MA, USA) for 1 h at room temperature. The nucleus was counterstained with Hoechst 33342 (Solarbio, Beijing, China). Images were captured using a Leica TCS SP8 confocal microscope (Leica, Wetzlar, Germany).

### 2.8. Tandem Mass Tags (TMT) Quantitative Proteome Analysis

Two groups of cells, CHMp vs. CHMm and CHMm-OE vs. CHMm, were collected and TMT-based quantitative proteome analysis was performed using the Shanghai Luming Biotechnology Co., Ltd. The ProteomeDiscovererTM 2.4.1.15 (ThermoFisher Scientific, Waltham, MA, USA) was used for protein identification and quantification using the UniProt Canis lupus familiaris database (Organism ID 9615). DEPs were screened from the listed protein candidates according to the Score Sequest HT > 0 criteria and unique peptide ≥ 1. Foldchange (FC) ≥ 1.2 or FC < 1/1.2 and Student’s *t*-test *p*-value < 0.05 were set as the threshold for screening the proteins with significant differences. Gene ontology (GO), Kyoto Encyclopedia of Genes and Genomes (KEGG) pathway analysis, and Gene Set Enrichment Analysis (GSEA) were used to investigate the biological function of these DEPs. *p*-value < 0.05 was considered statistically significant.

### 2.9. Quantitative Reverse-Transcription PCR (RT-qPCR)

According to the manufacturer’s instructions, total RNA was extracted from cells using RNAiso Plus (TaKaRa, San Jose, CA, USA). Reverse transcription was performed using the Primer-ScriptTM RT reagent Kit with gDNA Eraser (TaKaRa, San Jose, CA, USA). A total of 1 μL of each 10-fold diluted cDNA sample, along with 0.8 μL of the upstream and downstream primers (10 μM), 7.4 μL of deionized water, and 10 μL of 2 × SYBR Green Low ROX RT-qPCR Master Mix (Selleck, Shanghai, China), was used for each PCR reaction. Furthermore, GAPDH was used as an internal control, and RT-qPCR was performed using a two-step amplification method. The primer sequence is shown in Supplementary Table S1.

### 2.10. Western Blot

Cells were lysed in RIPA buffer (Solarbio, Beijing, China) for 40 min and the supernatant was collected after centrifugation (13,000× *g*). The BCA assay (BOSTER, Wuhan, China) measured the protein concentration. The denatured proteins were separated by 8–12% SDS-PAGE gel electrophoresis and transferred to a PVDF membrane. The membrane was blocked with 5% skim milk at room temperature for 2 h and incubated with the primary antibody (Supplementary Table S2) overnight at 4 °C. The membranes were washed with TBST buffer and incubated with a secondary antibody [53,54] for an hour at room temperature. Subsequently, the target protein was visualized using an enhanced chemiluminescence reagent (MeilunBio, Dalian, China), and the membrane was imaged using the Chemi Doc XRS+ imaging system (BioRad, Hercules, CA, USA).

### 2.11. Observation of Migrasome Bodies by Scanning Electron Microscopy

Glass coverslips (20 mm) pretreated with tissue culture treatment (Solarbio, Beijing, China) were placed in a 12-well plate and seeded with 5 × 10^3^ cells/well. After 24 h of cell culture, the medium was removed with PBS, and the cells were fixed with 3% glutaraldehyde. Subsequently, the samples were sent to Sichuan Lai Si Nuo Biotechnology Co., Ltd. (Sichuan, China) for analysis using a JSM-IT700HR scanning electron microscope (Tokyo, Japan).

### 2.12. Data Analysis

All data were expressed as the mean ± SD and statistical analysis was performed using GraphPad PrismTM 8 software with one-way ANOVA and two-way ANOVA. *p* < 0.05 indicated a significant difference, while *p* < 0.01 indicated an extremely significant difference.

## 3. Results

### 3.1. Difference of Fascin-1 Expression between CHMm and CHMp Cells

**Figure 1:**
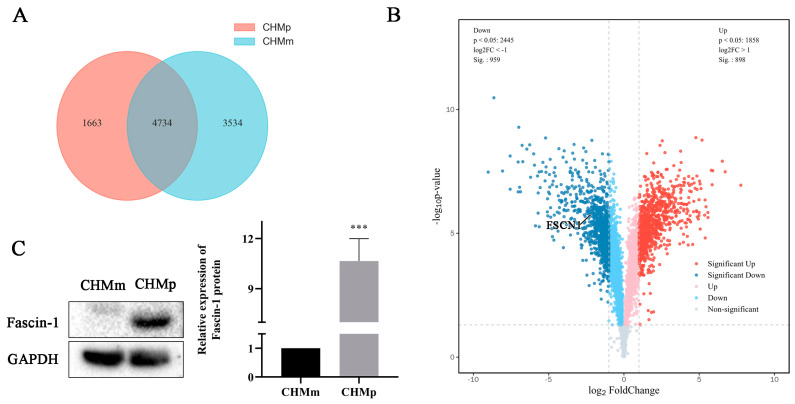
The expression difference of the Fascin-1 protein between the CHMm and CHMp cell lines. (**A**) Analysis of the proteomic data of CHMm and CHMp cell lines revealed 4734 credible protein data. (**B**) FC ≥ 2 and FC < ½ and *p* < 0.05 were set as the thresholds to screen the data, 1857 DEPs in CHMm vs. CHMp were obtained, and the volcano map was drawn. The lower dashed line separates the significant protein and the non-significant protein in DEPs, the left dashed line represents the protein of log2FC < −1, and the right dashed line represents log2FC > 1. (**C**) The Fascin-1 protein was detected by Western blot. *** means *p* < 0.001.

Comparison of the proteomic data of CHMp and CHMm cells revealed a total of 4734 credible proteins (Figure 1A). With FC ≥ 2 and FC < ½ and *p* < 0.05 set as the thresholds, 1857 DEPs were identified, of which 898 were upregulated and 959 were downregulated. Moreover, it was found that the expression of Fascin-1 in the metastatic cell line CHMm was much lower than that in the in situ cell line CHMp (Figure 1B), and its expression was verified using Western blot (Figure 1C). The details of the DEPs are summarized in Supplementary Table S3.

### 3.2. The Overexpression and Knockdown of Fascin-1 in CMT Cells

**Figure 2:**
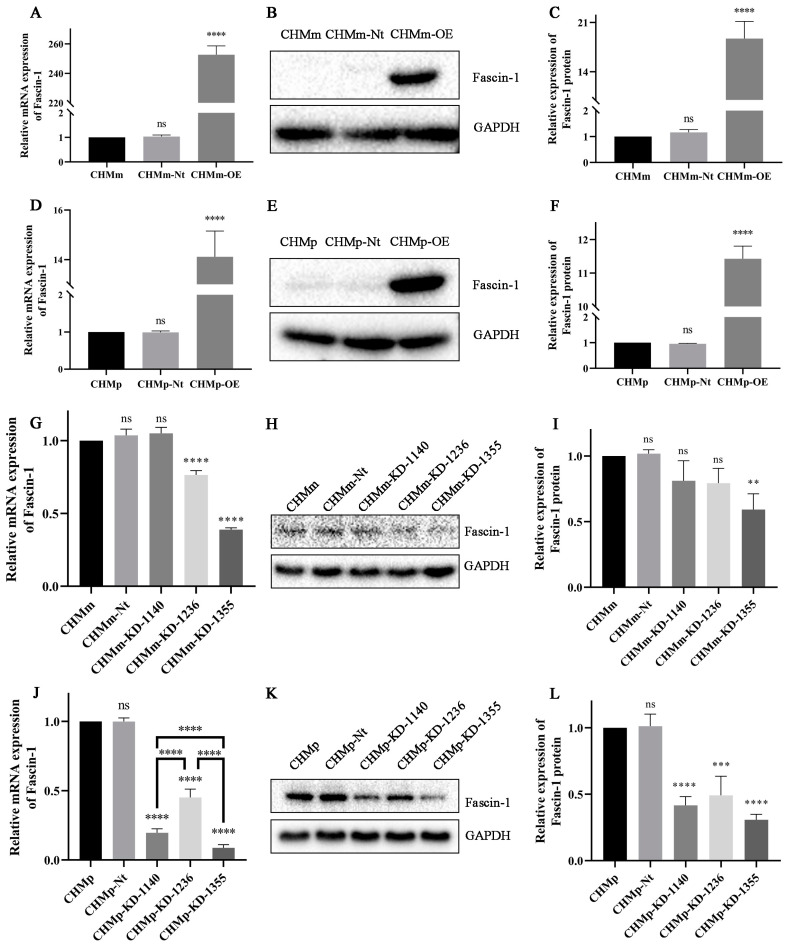
Functional characterization of the CHMm-OE cell line. (**A**) The mRNA expression of Fascin-1 was overexpressed in CHMm-OE cells. (**B**,**C**) Western blot analysis showed that Fascin-1 protein expression was overexpressed in CHMm-OE cells. (**D**) The mRNA expression of Fascin-1 was overexpressed in CHMp-OE cells. (**E**,**F**) Western blot analysis showed that the Fascin-1 protein was overexpressed in CHMp-OE cells. (**G**) The mRNA expression of Fascin-1 was knocked down in CHMm cells. (**H**,**I**) Western blot analysis showed that Fascin-1 protein expression was knocked down in CHMm-KD-1140/1236/1355 cells. (**J**) The mRNA expression of Fascin-1 was knocked down in CHMp cells. (**K**,**L**) Western blot analysis showed that Fascin-1 protein expression was knocked down in CHMp-KD-1140/1236/1355 cells. Significant differences were identified at ns *p* > 0.05, ** *p* < 0.01, *** *p* < 0.001, and **** *p* < 0.0001.

The CHMm-OE and CHMp-OE cell lines that overexpressed Fascin-1 were established. The expression of Fascin-1 in CHMm-OE cells was significantly higher than that in CHMm-Nt/CHMm cells at both the mRNA (Figure 2A) and protein (Figure 2B,C) levels (*p* < 0.001). Furthermore, the expression of Fascin-1 in CHMp-OE cells was significantly higher than that in CHMp-Nt/CHMp cells at both the mRNA (Figure 2D) and protein (Figure 2E,F) levels (*p* < 0.001).

At the mRNA level (Figure 2G), there was no significant difference in mRNA expression between CHMm cells and CHMm-KD-1140 cells. CHMm-KD-1236 and CHMm-KD-1355 showed extremely significant differences (*p* < 0.0001). However, at the protein level (Figure 2H,I), there was no statistically significant difference between CHMm-KD-1140 and CHMm-KD-1236 cells compared to CHMm cells (*p* > 0.05). In contrast, the expression of the Fascin-1 protein in CHMm-KD-1355 decreased by 40.79%, demonstrating significant differences (*p* < 0.0001).

For CHMp cells, all three target transcripts of shRNA could significantly reduce the Fascin-1 protein mRNA levels (Figure 2J, *p* < 0.0001). At the protein level, CHMp-KD-1236 significantly decreased the level of Fascin-1 (*p* < 0.001), while CHMp-KD-1140 and CHMp-KD-1355 (*p* < 0.01) significantly reduced the protein levels (*p* < 0.0001), with the knockdown efficiency of CHMp-KD-1355 at 69.23%. Subsequent experiments used CHMm-KD-1355 and CHMp-KD-1355 cells as knockdown cell lines for CHMm and CHMp cell lines, labeled as CHMm-KD and CHMp-KD, respectively. GAPDH was used as the loading control (Figure 2C).

### 3.3. Cell Viability, Migration, Adhesion, and Invasion in CMT Cells

**Figure 3:**
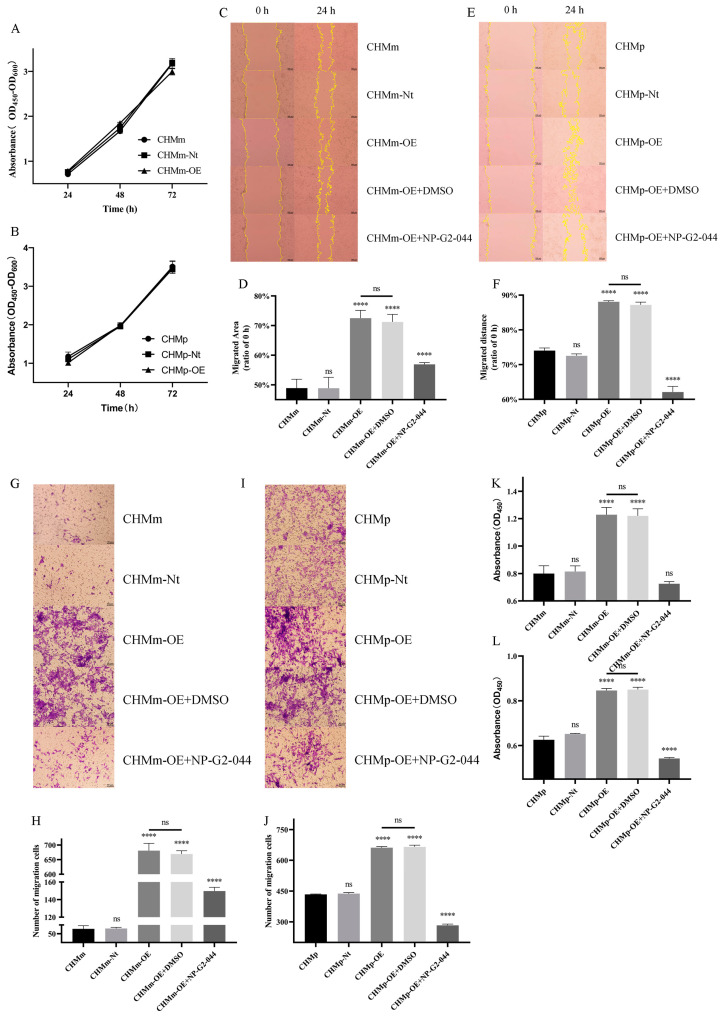
Cell viability, migration, adhesion, and invasion in CHMm-OE and CHMp-OE cells. (**A**,**B**) Detection of the proliferation ability of the Fascin-1 protein before and after overexpression and in negative control cell lines. The scale bars in the images represent 100 μm. (**C**,**E**) Under an objective lens of 10×, images in the scratch and healing areas are shown after 24 h. (**D**,**F**) Comparison of migrated area. (**G**,**I**) Photos of invasion assay under that objective lens of 20×. The scale bars in the images represent 50 μm. (**H**,**J**) Comparison of the number of migrating cells. (**K**,**L**) Light absorption value of successfully adhered cells after CCK-8 treatment for 1 h. Significant differences were identified at ns *p* > 0.05, and **** *p* < 0.0001.

From the proliferation assay (Figure 3A,B), the proliferation of CHMm-Nt and CHMm-OE at each time showed no difference compared to CHMm (Figure 3A, *p* > 0.05). Additionally, the proliferation of CHMp-Nt and CHMp-OE at each time point showed no difference compared to CHMp (Figure 3B, *p* > 0.05). These findings indicate that the increase in Fascin-1 expression does not affect the proliferation of CHMm and CHMp cells.

In the scratch assay (Figure 3C–F), comparing the scratch area at 24 h with 0 h (Figure 3C,E), the overexpression of Fascin-1 significantly increased the migration ability of CHMm-OE and CHMp-OE cells (Figure 3D,F, *p* < 0.0001). In contrast, treatment with the Fascin-1 specific inhibitor NP-G2-044 significantly inhibited the enhanced migration ability caused by Fascin-1 overexpression (Figure 3D,F, *p* < 0.0001). There was no significant difference in migration ability between the CHMm-Nt and CHMm cells, CHMm-OE and CHMm-OE + DMSO groups, CHMp-Nt and CHMp cells, and CHMp-OE and CHMp-OE + DMSO groups (Figure 3D,F, *p* > 0.05), indicating that increased Fascin-1 expression enhanced the migration ability of CHMm and CHMp cells.

From the invasion assay (Figure 3G–J), the invasion ability of CHMm-OE compared to CHMm/CHMm-Nt (Figure 3G,H) and CHMp-OE compared to CHMm/CHMm-Nt (Figure 3I,J) was significantly enhanced (*p* < 0.0001). Additionally, NP-G2-044 could suppress the invasion ability to a level close to CHMm/CHMm-Nt (*p* < 0.0001). There was no significant difference in invasion ability between CHMm-OE/CHMp-OE + DMSO group and CHMm-OE/CHMp-OE group (Figure 3H,J, *p* > 0.05), indicating that increased Fascin-1 expression can enhance the invasion ability of CHMm and CHMp cells.

From the adhesion assay (Figure 3K,L), the overexpression of Fascin-1 enabled CHMm-OE (Figure 3K) and CHMp-OE (Figure 3L) cells to acquire stronger adhesion ability in a difficult adhesion environment, with significantly more cells successfully adhering at 6 h compared to CHMm/CHMm-Nt and CHMp/CHMp-Nt cells (*p* < 0.0001). Moreover, treatment with NP-G2-044 significantly reduced the adhesion ability of CHMm-OE and CHMp-OE cells (*p* < 0.0001). There was no significant difference in adhesion ability between the CHMm-OE/CHMp-OE + DMSO group and the CHMm-OE/CHMp-OE group (Figure 3K,L, *p* > 0.05), indicating that increased Fascin-1 expression enhanced the adhesion ability of CHMm and CHMp cells.

**Figure 4:**
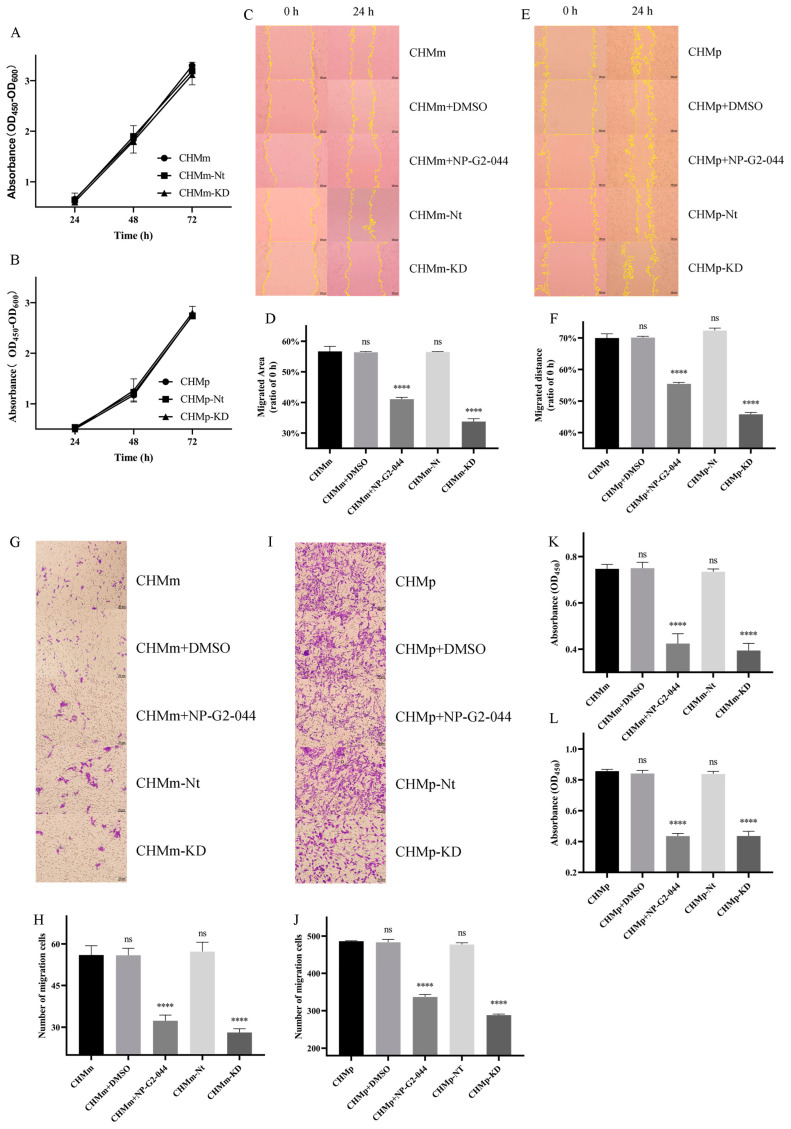
Cell viability, migration, adhesion, and invasion in CHMm-KD and CHMp-KD cells. (**A**,**B**) Detection of the proliferation ability of the Fascin-1 protein before and after overexpression and in negative control cell lines. The scale bars in the images represent 100 μm. (**C**,**E**) Under an objective lens of 10×, images in the scratch and healing areas are shown after 24 h. (**D**,**F**) Comparison of migrated area. (**G**,**I**) Photos of invasion assay under an objective lens of 20×. The scale bars in the images represent 50 μm. (**H**,**J**) Comparison of the number of migrating cells. (**K**,**L**) Light absorption value of successfully adhered cells after CCK-8 treatment for 1 h. Significant differences were identified at ns *p* > 0.05, and **** *p* < 0.0001.

From the proliferation assay (Figure 4A,B), the knockdown of the Fascin-1 protein did not affect the proliferation capacity of CHMm and CHMp cells (*p* > 0.05).

From the scratch assay (Figure 4C–F), comparing the scratch area at 24 h with 0 h (Figure 4C,E), the knockdown of Fascin-1 significantly reduced the migration ability of CHMm-KD and CHMp-KD cells (Figure 4D,F, *p* < 0.0001). In contrast, the migration ability of CHMm and CHMp cells was significantly inhibited in the NP-G2-044 group (*p* < 0.0001). There was no significant difference in migration ability between the CHMm, CHMm + DMSO, and CHMm-Nt groups, as well as between the CHMp, CHMp + DMSO, and CHMp-Nt groups (*p* > 0.05), indicating that the knockdown of Fascin-1 can weaken the migration ability of CHMm and CHMp cells.

From the invasion assay (Figure 4G–J), the CHMm-KD, CHMp-KD, and NP-G2-044 treatment groups showed a significant decrease in invasion ability compared to CHMm and CHMp cells (*p* < 0.0001). There was no significant difference in invasion abilities between the CHMm, CHMm + DMSO, and CHMm-Nt groups, as well as between the CHMp, CHMp + DMSO, and CHMp-Nt groups (*p* > 0.05), indicating that the knockdown of Fascin-1 can weaken the invasion ability of CHMm and CHMp cells.

From the adhesion assay (Figure 4K,L), the knockdown of the Fascin-1 protein led to a decrease in the adhesion ability of CHMm-KD and CHMp-KD cells, with significantly fewer cells successfully adhering at 6 h compared to CHMm and CHMp cells (*p* < 0.0001). Additionally, treatment with NP-G2-044 significantly reduced the adhesion ability of CHMm and CHMp cells (*p* < 0.0001). There was no significant difference in adhesion ability between the CHMm, CHMm + DMSO, and CHMm-Nt groups, as well as between the CHMp, CHMp + DMSO, and CHMp-Nt groups (Figure 4K,L, *p* > 0.05), indicating that the knockdown of Fascin-1 can weaken the adhesion ability of CHMm and CHMp cells.

In summary, the expression level of the Fascin-1 protein does not affect the proliferation of CMT cells. The migration, adhesion, and invasion ability of CHMm-OE and CHMp-OE were significantly enhanced by the overexpression of Fascin-1. Simultaneously, the knockdown of the Fascin-1 protein inhibited the migration, adhesion, and invasion of CHMm-KD and CHMp-KD.

### 3.4. Fascin-1 Overexpression Enhances the Formation of Filopodia and Lamellipodia on the Surface of CHMm Cells

Vasodilator-stimulated phosphoprotein (VASP), a significant protein in actin dynamics, protects the polymerization of actin at the front end by promoting the formation of stress fibers. Notably, the localization of VASP allows us to determine the range and extension of lamellipodia.

**Figure 5:**
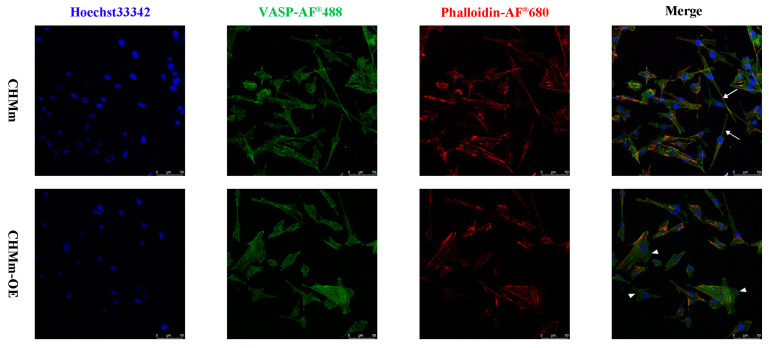
The overexpression of Fascin-1 increased the filopodia and lamellipodia in CHMm-OE cells. Immunocytochemical staining is used as follows: VASP (AF^®^448-green), Phalloidin (AF^®^680-red), and Hoechst 33342 (blue). The arrow indicates filopodia, and the triangle indicates plate lamellipodia. The scale of filopodia and lamellipodia in CHMm-OE is larger than that of the CHMm cell line.

Filopodia, thin and elongated protrusions on the extracellular membrane, were observed in CHMm cells (Figure 5). In CHMm-OE cells, the number of VASP spots at the leading edge of lamellipodia decreased compared to CHMm cells. These results suggest that Fascin-1 can promote the rearrangement of the cell skeleton, increasing the formation of filopodia and lamellipodia in CMT cells.

### 3.5. Differential Expression of the Proteins

**Figure 6:**
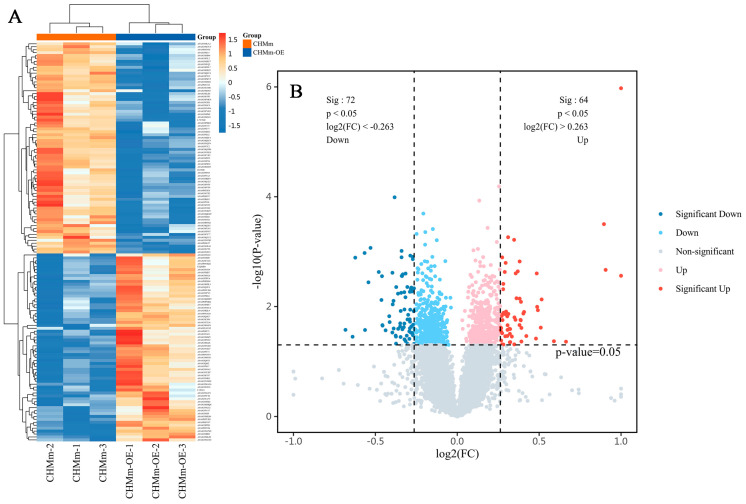
Proteomic analysis of CHMm and CHMm-OE cell lines. (**A**) In the thermogram, the abscissa is the comparison group, and the ordinate is the number of DEPs. Different colors display up and down. (**B**) In the volcano map, red represents significantly differentially upregulated proteins (FC > 1.2, *p* < 0.05), blue represents significantly downregulated proteins (FC < 1/1.2, *p* < 0.05), and the darker color means a more significant difference. Gray represents non-differentially or non-statistically significant proteins.

A total of 8720 proteins with unique peptide sequences were identified in CHMm-OE vs. CHMm. The significance threshold was set as FC ≥ 1.2 and FC < 1/1.2, *p* < 0.05. Additionally, 136 DEPs were identified in CHMm-OE when compared with CHMm, including 64 upregulated proteins and 72 downregulated proteins, which were drawn on the volcano map (Figure 6B). The top 15 up- or downregulated proteins in CHMm-OE vs. CHMm are shown in Table 1.

### 3.6. Cluster Analyses of Differentially Expressed Proteins

**Figure 7:**
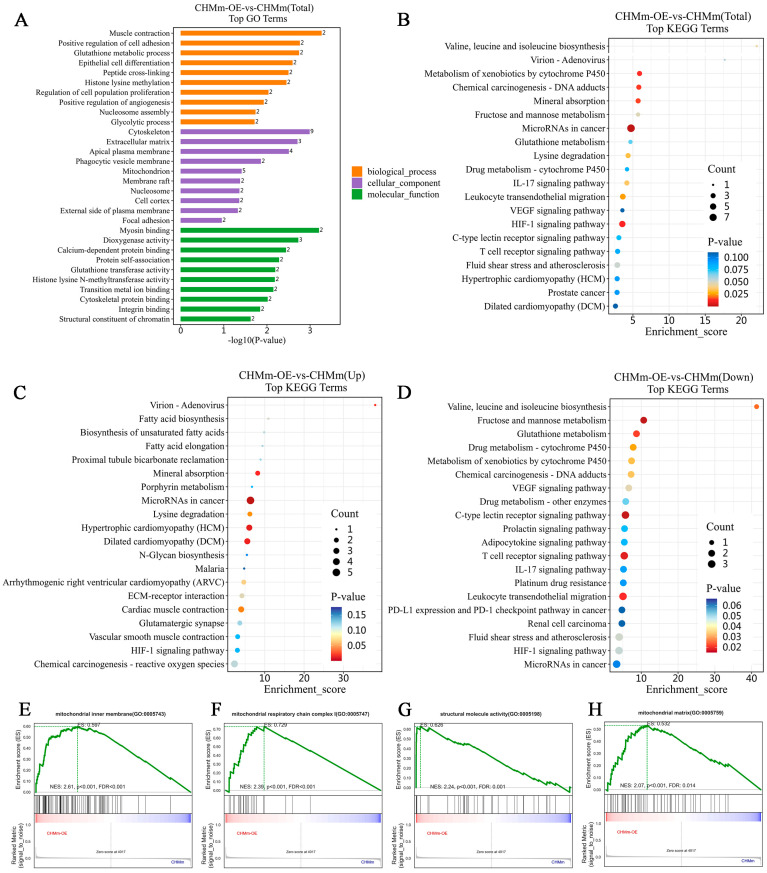
Proteomic analysis of DEPs in CHMm-OE vs. CHMm by GO, KEGG, and GSEA. (**A**) The top 10 of three ontologies in GO analysis. (**B**–**D**) The top 20 terms of total (**B**), upregulated (**C**), and downregulated (**D**). (**E**–**H**) GSEA results: a graphical view of the normalized enrichment score (NES) for a gene set between CHMm-OE and CHMm.

To understand the functions of the DEPs and the signaling pathways they are involved in, 136 proteins were annotated using GO, KEGG, and GSEA. The data patterns of the three biological replicate groups were highly similar (Figure 7A).

For GO analysis, predominantly enriched proteins in the biological process (BP) were associated with the following terms: muscle contraction, positive regulation of cell adhesion, glutathione metabolic process, and epithelial cell differentiation, among others. The predominantly enriched terms in the cellular component (CC) category were cytoskeleton, extracellular matrix, apical plasma membrane, mitochondrion, focal adhesion, etc. DEPs in the molecular function (MF) category were associated with the following terms: myosin binding, dioxygenase activity, calcium-dependent protein binding, glutathione transferase activity, etc. Furthermore, the top 10 significantly enriched terms in these three ontologies, biological processes, cellular components, and molecular functions, are shown in Figure 7A.

Further analysis of the KEGG signaling pathways for these DEPs demonstrated that they were involved in the IL-17 signaling pathway, the VEGF signaling pathway, the HIF-1 signaling pathway, reactive oxygen species, the platinum drug resistance, and the ECM–receptor interaction. The top 20 KEGG pathways in the list of DEPs are shown in Figure 7B–D.

The change in proteins following Fascin-1 overexpression intervention was comprehensively analyzed, which wielded GSEA (Figure 7). There were four regulated gene sets identified: mitochondrial inner membrane (GO:0005743), mitochondrial respiratory chain complex I assembly (GO:0032981), structural molecule activity (GO:0005198), and mitochondrial matrix (GO:0005759). These results indicated that the therapeutic effect of Fascin-1 on metastasis is likely closely implicated in mitochondria and cytoskeleton regulation.

### 3.7. Fascin-1 Overexpression Changes the Expression of Tumor Metastasis-Related Proteins

From the data mentioned above, it was found that the expressions of tissue inhibitor of matrix metalloproteinase 1 (TIMP1) and matrix metalloproteinase 3 (MMP3) in CHMm-OE cells were upregulated. These findings may likely contribute to the enhancement of cell metastasis.

**Figure 8:**
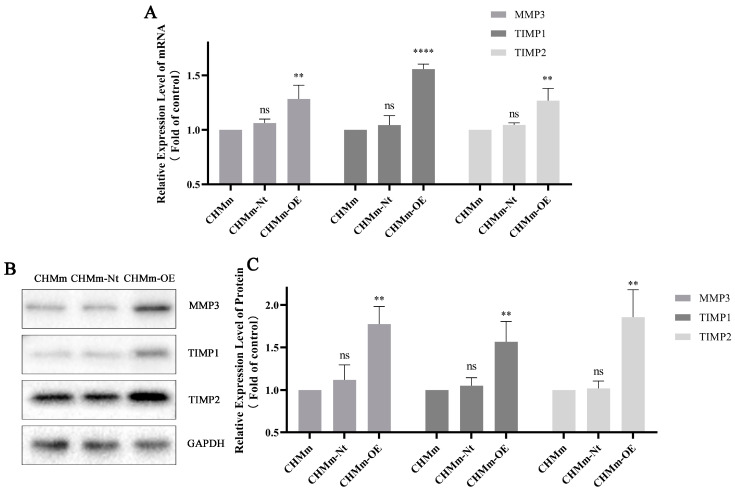
Changes in key protein expression during tumor invasion. (**A**) As assessed by RT-qPCR, the change in MMP3, TIMP1, and TIMP2 mRNA expression in CMT cells (**B**) Representative image of Western blot analysis. (**C**) The results of densitometric analysis of the Western blot showed the protein expression levels of MMP3, TIMP1, and TIMP2 in CHMm-OE versus CHMm/CHMm-Nt. GAPDH was blotted to ensure equal loading. The significant differences at the expression level in the CHMm-OE were observed at ns *p* > 0.05, ** *p* < 0.01, and **** *p* < 0.0001.

MMP3, TIMP1, and TIMP2 play critical roles in tumor invasion [55]. The overexpression of the Fascin-1 protein significantly increased their expression levels in CMT cells (Figure 8). It was also previously reported that Fascin-1 can enhance cell migration and invasion in vivo by affecting the expression of MMPs [56]. Interestingly, the expression of MMP2 and MMP9 was not detected in the CHMm-OE cells. However, their corresponding inhibitory proteins, TIMP1 and TIMP2, were upregulated as revealed by proteomic data and Western blotting.

### 3.8. Effect of Fascin-1 Overexpression on the EMT-Related Protein Expression

**Figure 9:**
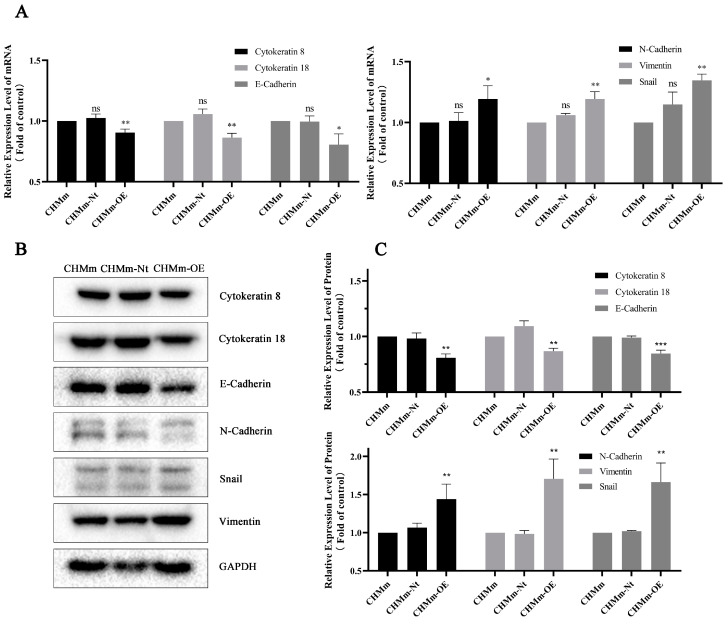
Fascin-1 promotes the EMT process in CMT cells. EMT marker mRNA expression changes in CMT cells assessed by RT-qPCR (**A**). (**B**) Western blot analysis shows Cytokeratin 8/18, E-cadherin, N-cadherin, Snail, and vimentin expression in CHMm/CHMm-Nt and CHMm-OE. (**C**) Quantification of the relative expression of band intensity in the experiment. Three independent replicates were determined using ImageJ (v. 1.53), and the results are shown in B. GAPDH was blotted to ensure equal loading. At the mRNA and protein levels, the expression of Cytokeratin 8/18 and E-cadherin is downregulated following the overexpression of Fascin-1. In contrast, the expression of N-cadherin, vimentin, and Snail is upregulated. The significant differences between CHMm-OE and CHMm/CHMm-Nt were indicated at ns *p* > 0.05, * *p* < 0.05, ** *p* < 0.01, and *** *p* < 0.001.

In HBC, EMT is a hallmark of tumor aggressiveness and is associated with cell migration, invasion, and metastasis. In canines, EMT is also an indicative sign of the invasiveness of breast tumors [57,58]. Among them, Fascin-1 can change the expression level of EMT marker proteins (including mesenchymal vimentin and the epithelial protein E-cadherin) [59,60,61,62]. Thus, EMT is initiated, and epithelial tumor cells’ migration and morphological changes are promoted. At the same time, in the proteomic data, the EMT-related protein N-cadherin (gene name CDH2) was upregulated in CHMm-OE. Therefore, to further study the metastasis enhancement effect of the overexpression of Fasicn-1 in CHMm-OE, we detected the expression changes in the EMT-related protein and epithelial cell-related protein. As shown in Figure 9, E-cadherin, Cytokeratin 8, and Cytokeratin 18 were downregulated, while vimentin, N-cadherin, and Snail were upregulated in CHMm-OE cells.

### 3.9. Effect of Fascin-1 Expression on the Formation of Migration

**Figure 10:**
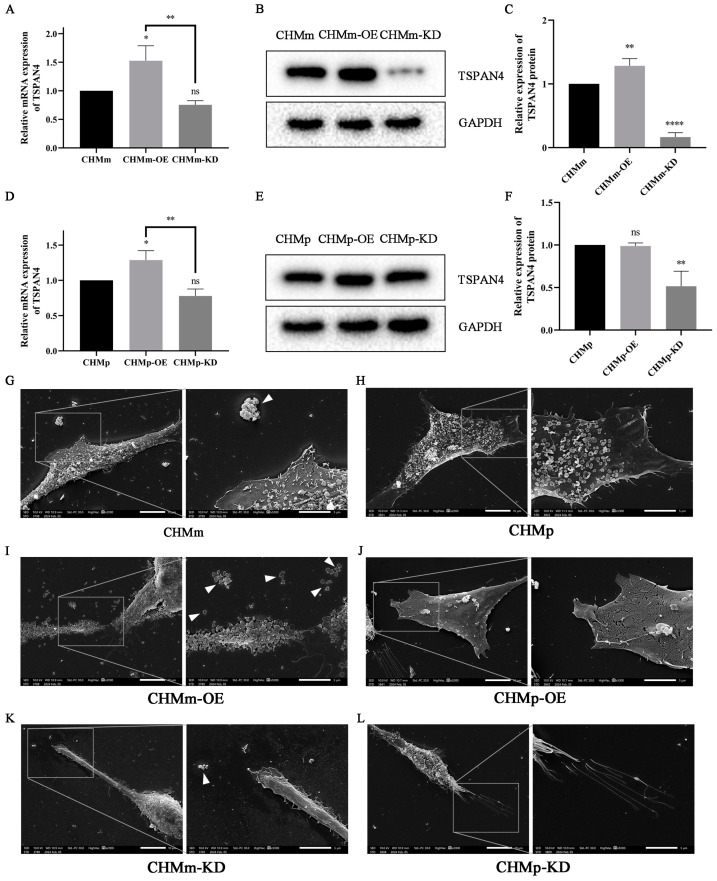
Effect of Fascin-1 on the formation of cell migration. (**A**) The mRNA expression level of TSPAN4 protein in CHMm, CHMm-OE, and CHMm-KD cells. (**B**,**C**) Western blot was used to detect the expression changes in TSPAN4 protein in CHMm, CHMm-OE, and CHMm-KD cells. (**D**) The expression level of TSPAN4 mRNA in CHMp, CHMp-OE, and CHMp-KD cells. (**E**,**F**) The expression of TSPAN4 protein in CHMp, CHMp-OE, and CHMp-KD cells was detected by Western blot. (**G**–**L**) SEM images of CHMm, CHMm-OE, CHMm-KD, CHMp, CHMp-OE, and CHMp-KD, respectively. Triangle refers to the migration formed by cells. The significant differences at the expression level in the CHMm-OE were observed at ns *p* > 0.05, * *p* < 0.05, ** *p* < 0.01, and **** *p* < 0.0001.

Proteomic analysis indicated that the migration marker protein Tetraspanin 4 (TSPAN4) expression level was upregulated when Fascin-1 was overexpressed. Therefore, the expression levels of TSPAN4 were examined in the remaining six cell lines in addition to the negative control cell lines. At the mRNA level (Figure 10A,D), there was no significant difference in TSPAN4 levels between CHMm and CHMp cells and their corresponding Fascin-1 knockdown cell lines (*p* > 0.05). In contrast, the mRNA levels of TSPAN4 in CHMm-OE and CHMp-OE cells were significantly increased (*p* < 0.001).

At the protein level, both the overexpression and knockdown of Fascin-1 could influence the expression level of the TSPAN4 protein (Figure 10B,E). Specifically, the overexpression of Fascin-1 led to an upregulation of TSPAN4 expression in CHMm-OE cells (Figure 10C, *p* > 0.01). In comparison, the knockdown of Fascin-1 resulted in a decrease in TSPAN4 expression in CHMm-KD (Figure 10C, *p* < 0.0001) and CHMp-KD (Figure 10F, *p* < 0.01) cells.

Under scanning electron microscopy (SEM), more migration bodies were observed around CHMm-OE cells than CHMm and CHMm-KD cells. In contrast, no migration bodies were detected in CHMp, CHMp-OE, and CHMp-KD cells.

## 4. Discussion

Cell migration is a complex process in which the cross-linking of F-actin with Fascin-1 provides support for filopodia and lamellipodia at the cell’s leading edge [32,63]. Moreover, cytoskeleton remodeling is one of the critical early events in cancer cells that acquire invasiveness [64].

In this study, we found that the Fascin-1 protein was rarely expressed in CHMm cells and we established a Fascin-1 overexpression CHMm-OE cell line. The CHMm, a metastatic mammary cancer cell line is derived from CMT abdominal metastases [52]. Notably, the low expression of Fascin-1 in CHMm may be related to its successful colonization after metastasis. Therefore, we overexpressed Fascin-1 in the CHMm cell line and studied its role in CMT. Compared with the CHMm and CHMp cell lines, the migration, adhesion, and invasion abilities of Fascin-1 overexpressed cell lines were enhanced, while those of knockdown cell lines were decreased. Moreover, the overexpression and knockdown of Fascin-1 did not affect the proliferation ability of CMTs. These findings are consistent with various studies on Fascin-1 in human cancers [24,25,26], revealing that Fascin-1 plays the same role in the migration, adhesion, and invasion of CMT as in human cancer.

In addition to cross-linking F-actin, Fascin-1 is also involved in other cell movement processes. The binding ability of the actin bundle to Ena/VASP is stronger than that of filamentous F-actin [65,66]. Moreover, the EVH1 domain of the VASP protein binds to Fascin-1, strengthening the binding between Ena/VASP and F-actin and promoting F-actin extension [42,67,68,69]. From the immunofluorescence assay, the localization of VASP protein on the pseudopodia of CHMm-OE cells increased, indicating that Fascin-1 may also exist in the interaction of VASP protein in CMT.

Meanwhile, Fascin-1 in the cytoplasm plays a significant role in stabilizing the tension of stressed fibers [31], promoting the turnover of cell focal adhesions together with Cofilin [46] and strengthening the cell adhesion ability.

When the Fascin-1 protein is knocked down or knocked out, the activity of Myosin II on stress fibers is no longer restricted by Fascin-1. Thus, this results in a stronger tension exerted on FAs by the stress fibers, enhancing cell traction force on the ECM. When the tension exceeds FAs’ strength, FAs undergo disassembly [31]. Simultaneously, the reduction in filopodia alters the number of cell protrusions, leading to decreased Rac-dependent migration mediated by layer adhesion protein and impairing the turnover of FAs [70]. Consequently, this slows down cell migration and invasion speed.

MMPs are proteases that help tumor cells to decompose basement membranes and ECM, and they are a common secreted protein in metastatic tumors [71]. According to recent studies, Fascin-1 enhances the invasive ability of pancreatic cancer and liver cancer cells by increasing the expression of MMP2 and MMP9, which are primarily responsible for the degradation of Type IV collagen [24,72]. However, in the process of ECM degradation, different MMPs primarily degrade different extracellular matrix components. The MMPs of the Stromelysin class (such as MMP3 and MMP-10) can degrade fibronectin, laminin, Type IV and V collagen fibers, and protein glycosaminoglycans and also play crucial roles in ECM degradation. In this study, MMP3 was upregulated in CHMm-OE cells but the expression of MMP2 and MMP9 was not detected at the transcriptional and protein levels and this expression difference may be influenced due to the tumor microenvironmental factor. Additionally, the lack of MMP10 detection is also a limitation of this study. The noncovalent complex formed by TIMPs and MMPs can maintain the integrity of cell connections, reduce tumor metastasis, and improve prognosis by reducing ECM degradation [73,74]. However, the imbalance of MMPs/TIMPs may affect the invasion and metastasis of tumors [75]. Likewise, this imbalance may cause the enhanced invasive ability of CHMm-OE and CHMp-OE.

EMT, as mentioned by the proteomic results, is one of the key mechanisms regulating cancer pathogenesis [76] and plays a role in tumor progression and metastasis [77]. The transcription factor Snail can cause the loss of E-cadherin and tight junction proteins but upregulate fibronectin and other biomarkers [78,79]. Notably, this study found that Fascin-1 overexpression significantly decreased E-cadherin and increased vimentin, Snail, and N-cadherin expression levels, consistent with previous studies [24,25]. At the same time, Snail enhances cell membrane fluidity [59] and plays a role in tumor progression and metastasis [77]. Moreover, its high expression is related to the poor prognosis of tumor patients [80]. In this study, both the epithelial cell marker E-cadherin and mesenchymal cell marker N-cadherin were present in CHMm-OE cells, indicating that CHMm-OE cells are in an intermediate epithelial/mesenchymal (E/M) transitional state between a more invasive epithelial and mesenchymal phenotype [81]. This may provide an elucidation for how CHMm-OE cells maintain adhesion while also possessing a stronger migratory ability. Collectively, Fascin-1 is involved in the EMT process of mammary tumors in CMT and promotes cell migration and invasion. The changes in this process are also consistent with previous reports in CMT [60].

Migrasomes are located at the end and bifurcation of retraction fibers in the tail of migrating cells [82]. As the cell metastasizes, an unequal number of small vesicles inside the migrasome are released into the ECM, and absorbed by neighboring cells to enhance the migration ability to receive cells [83,84,85]. At the same time, the overexpression of Fascin-1 can improve the ability and motility of migrating cells to sense the surrounding microenvironment at the leading edge.

However, migration bodies have not been reported in the CMT cell line, and the specific regulatory relationship between migration bodies and Fascin-1 remains unclear. Nevertheless, the results of proteomic analysis and validation experiments in this study demonstrate that the expression level of Fascin-1 can influence the TSPAN4 protein. Discrepancies in the number of migration bodies observed in SEM between CHMm and CHMp cells suggest that specific signaling pathways altered during tumor adaptation to the secondary tissue environment and proliferation processes may confer the ability to generate migration bodies in CHMm cells. Notably, TSPAN4, a migrasome-related protein upregulated by the overexpression of Fascin-1, may achieve the collective migration of cells through the migrasome. Furthermore, this may provide clues for the performance of CHMm-OE in the cell scratch assay.

Research has found that when cells undergo mild mitochondrial stress, the “mitochondrial autophagy” process is activated, resulting in the relocation of damaged mitochondria to the migration bodies, which are then cleared by migrating cells [86]. This suggests that the mitochondrial changes in the Fascin-1 proteomics of CHMm and CHMm-OE may be related to the increase in migration bodies. However, the changes in the number of migration bodies in CHMm and CHMp cells were different, as observed in scanning electron microscopy. This may be influenced due to changes in certain signaling pathways during the process of tumor adaptation and proliferation in the secondary microenvironment, allowing CHMm cells to acquire the ability to generate migration bodies.

KEGG analysis showed changes in HIF-1 signaling pathways. Additionally, the key protein Hypoxia-inducible factor-1 (HIF-1), a regulatory factor for cancer cells to overcome environmental stress [87], promoted the EMT process by binding with Snail [88,89].

Other enriched signal pathways, such as the IL-17 signaling pathway [90] and the VEGF signaling pathway [91,92], can promote HBC metastasis and the EMT process. Moreover, the circulating HBC cells can produce reactive oxygen species under hydrodynamic stress and promote cell migration and the EMT process via ERK/MAPK [93] and PI3K/Akt [94] signaling pathways [95,96].

The results of GSEA suggest that after the overexpression of Fascin-1, there are many mitochondrial gene sets in DEP enrichment analysis. A study by Lin et al. shows that Fascin-1 enhances mitochondrial DNA homeostasis by mediating mitochondrial F-actin cross-linking, thereby increasing the biogenesis of mitochondrial respiratory chain complex I [97]. Thus, this gives tumor cells the survival advantage in the tumor environment with nutritional deficiency. Meanwhile, the migrasome can maintain mitochondrial homeostasis by disposing of damaged mitochondria in migrating cells [86].

## 5. Conclusions

This study demonstrated that increased expression of Fascin-1 significantly enhances the in vitro transmigration ability of CMT cells, while the inhibition of Fascin-1 expression can prevent CMT cell transmigration in vitro. Importantly, Fascin-1 promotes the migration effect by promoting the formation of filopodia in CMT cells and the EMT process. Furthermore, it influences cell migration by upregulating the expression of TSPAN4.

## Figures and Tables

**Table 1:** The top 15 up- or downregulated proteins in CHMm-OE vs. CHMm cells.

Accession	Protein Name	Gene Name	Fold Change	*p*-Value
Upregulated proteins				
A0A8C0T2U6	Metalloproteinase inhibitor 1	TIMP1	2.1842	0.0028
A0A8P0T8T4	Ubiquitin carboxyl-terminal hydrolase isozyme L1	UCHL1	1.8738	0.0021
A0A8I3MRJ3	Actin like 8	ACTL8	1.8603	0.0003
A0A0H5AU05	Matrix metalloproteinase-3	MMP3	1.5845	0.0437
A0A8I3PYT6	Thymopoietin	TMPO	1.5054	0.0429
A0A8I3NNH3	Cadherin-2	CDH2	1.4293	0.0074
A0A8C0PBF7	SH3 domain-containing protein	SH3KBP1	1.4239	0.0242
A0A8C0TCB7	10 kDa heat shock protein, mitochondrial	LOC609748	1.4066	0.0117
A0A8P0TUR9	RRM domain-containing protein	-	1.4024	0.0101
A0A8C0S9A5	IF rod domain-containing protein	KRT13	1.3998	0.0025
A0A8C0TG32	Tetraspanin	TSPAN4	1.3953	0.0384
A0A8C0RGJ6	RBR-type E3 ubiquitin transferase	Pdr-1	1.3395	0.0345
E2QXB4	Large neutral amino acids transporter small subunit 3	SLC43A1	1.3269	0.0125
A0A8I3NPV3	Tetraspanin	UPK1B	1.3228	0.0134
A0A8C0N1I7	DNA helicase	DNA helicase	1.3120	0.0245
Downregulated proteins				
A0A8C0Q0V4	Importin 11	IPO11	0.8327	0.0047
A0A8C0PMC0	Poly(A) binding protein interacting protein 2B	PAIP2B	0.8326	0.0479
A0A8P0TJG8	Rap1 GTPase-GDP dissociation stimulator 1	RAP1GDS1	0.8319	0.0121
A0A8I3P5A5	1,4-alpha-glucan branching enzyme	GBE1	0.8296	0.0214
A0A8I3P398	GB1/RHD3-type G domain-containing protein	GBP6	0.8290	0.0333
A0A8C0PKV5	RNA pseudouridine synthase domain containing 2	RPUSD2	0.8286	0.0014
A0A8P0SE37	protein-tyrosine-phosphatase	PTPN11	0.8282	0.0012
A0A8C0SLE6	DNA repair protein SWI5 homolog	Swi5	0.8262	0.0304
A0A8C0QEC9	HECT domain and ankyrin repeat-containing E3 ubiquitin protein ligase 1	HACE1	0.8257	0.0053
A0A8I3PZ15	Transglutaminase 2	TGM2	0.8236	0.0230
A0A8C0Q3M0	6-phosphofructo-2-kinase/fructose-2,6-biphosphatase 3	PFKFB3	0.8235	0.0045
A0A8I3P7C2	TRAF-type zinc finger domain containing 1	TRAFD1	0.8215	0.0048
A0A8I3MJM1	Protein S100	S100A2	0.8215	0.0298
A0A8C0SW41	CKLF like MARVEL transmembrane domain containing 7	CMTM7	0.8198	0.0012
A0A8C0PU63	CobW C-terminal domain-containing protein	CBWD1	0.8195	0.0114

## Data Availability

Proteomics data used will be uploaded after the article is received.

## Supplementary Materials

The original image of Western blot, primers, antibodies and the list of differential proteins used in Figure 1 can be downloaded from https://www.mdpi.com/article/10.3390/vetsci11060238/s1; Supplementary Table S1: List of primers used by RT-qPCR. Supplementary Table S2: List of antibodies and dilution ratio used in Western blot. Supplementary Table S3: List of differentially expressed proteins in CHMp and CHMm proteomics.
