# Supplementary material for: Fascin-1 Promotes Cell Metastasis through Epithelial–Mesenchymal Transition in Canine Mammary Tumor Cell Lines
Source: Vet Sci. 2024 May 25;11(6):238. doi: 10.3390/vetsci11060238 (PMC11209228; doi:10.3390/vetsci11060238)
Supplement: Supplementary file 1 [file vetsci-11-00238-s001.zip › Supplementary material.pdf]

Fascin-1 re-expression promotes cell metastasis through epithelial-mesenchymal transition in canine mammary tumor CHMm cell

Table S1 RT-qPCR primer sequence

Name	Primer sequence (5'-3')
Fascin-1	Forward: GCGTCCAATGGCAAGTTTGT
（Gene ID: 489880）	Reverse: CTCCCCACGGAACACAATGA
E-Cadherin	Forward: ATGTGTTTGTGTGCGACTGC
（Gene ID: 442858）	Reverse: AATGCCCAAGATGGCAGGAA
N-Cadherin	Forward: TGCCATTATTGCCATCCTCCTGTG
（Gene ID: 480169）	Reverse: GCGTTCTTTATCTCGGCGTTTCATC
Vimentin	Forward: GACCGCTTCGCCAACTACATCG
（Gene ID: 477991）	Reverse: CTCCCGCATCTCCTCCTCGTAG
Cytokeratin 8	Forward: ACATCGAGATCGCCACCTACCG
（Gene ID: 486513）	Reverse: CGCTGGTCGTCTTCGTATGGATG
Cytokeratin 18	Forward: GTCTTACTGACGCCCTGGAC
（Gene ID: 477601）	Reverse: GGTCTCAGACACCACCTTGC
TIMP1	Forward: GCAGAAGTCAACCAGACCGA
（Gene ID: 403816）	Reverse: AGTATCCGCAGACGCTTTCC
TIMP2	Forward: TGATCCCGTGCTATATCTCGTCTCC
（Gene ID: 403633）	Reverse: CCTGATGCCCGTTGATGCTCTTC
Snail	Forward: TCGGACGAGGACAGTGGGAAAG
（Gene ID: 485924）	Reverse: AAGAGGCTGAGGTGGAAGAGAAGG
MMP3	Forward: ATGGACAAGCGATACATCAGGAACC
（Gene ID: 403445）	Reverse: CGAGTGAAAGAGACCCAGGGAATG
TSPAN4	Forward: GCCCAACGAGACCTGAAGAA
（Gene ID: 611422）	Reverse: GACCTCAAACCAGTCGGTGT
GAPDH	Forward: GATCCCGCCAACATCAAATG
（Gene ID: 403755）	Reverse: TCACGCCCATCACAAACATG

Table S2 Antibody use and dilution ratio

Name	Manufacturer	Dilution ratio
Fascin-1	Santa Cruz	1:1 000
E-Cadherin	Santa Cruz	1:1 000
N-Cadherin	Santa Cruz	1:1 000
Snail	Santa Cruz	1:1 000
TIMP1	Santa Cruz	1:1 000
TIMP2	CST	1:1 000
Vimentin	Abcam	1:20 000
Cytokeratin 8	Abcam	1:10 000
MMP3	Abcam	1:10 000
Cytokeratin 18	Proteintech	1:25 000
TSPAN4	NOVUS	1：500
GAPDH	Proteintech	1:40 000

Table S3 Differentially expressed proteins in CHMp and CHMm proteomics

Accession	Gene Name	Description	log2FoldChange	FoldChange	Regulation	p-value
A0A8P0T620	SEC31A	SEC31 homolog A, COPII coat complex component	-9.02177	0.001923874	Down	3.375E-08
A0A8C0PSC4	P4HA1	procollagen-proline 4-dioxygenase	-8.64195	0.002503305	Down	3.343E-11
A0A8C0P3R9	FLNB	Filamin B	-8.06511	0.003733877	Down	3.047E-08
A0A8C0PYQ2	HNRNPA1	Heterogeneous nuclear ribonucleoprotein A1	-7.562443333	0.005290269	Down	7.539E-09
A0A8C0PVK3	PPP2R1A	Protein phosphatase 2 scaffold subunit Aalpha	-7.56109	0.005295233	Down	1.657E-07
A0A8I3NY63	DNM1L	Dynamin-1-like protein	-7.030886667	0.00764702	Down	1.32E-08
A0A8P0NJW0	MTA1	Metastasis associated 1	-7.02976	0.007652994	Down	2.126E-07
A0A8I3MPP1	HNRNPK	Heterogeneous nuclear ribonucleoprotein K	-6.987116667	0.007882578	Down	5.196E-10
A0A8I3MV65	HNRNPM	Heterogeneous nuclear ribonucleoprotein M	-6.93467	0.008174408	Down	1.38E-07
A0A8I3P633	CCAR2	Cell cycle and apoptosis regulator 2	-6.87323	0.008530051	Down	2.097E-07
A0A8C0T4F5	-	Leucine-rich repeat flightless-interacting protein 1	-6.7691	0.009168491	Down	2.803E-09
A0A8I3MR06	ATXN2L	Ataxin 2 like	-6.721703333	0.009474705	Down	1.279E-08
A0A8C0P1V2	-	H1.2 linker histone, cluster member	-6.454246667	0.01140455	Down	3.965E-09
A0A8P0TN04	AHNAK	PDZ domain-containing protein	-6.26277	0.01302322	Down	2.668E-09
A0A8C0PMB6	-	Nucleolar and coiled-body phosphoprotein 1	-6.20287	0.01357532	Down	5.451E-08
A0A8P0S9S6	MRE11	Double-strand break repair protein	-5.996393333	0.015664111	Down	6.177E-09
A0A8C0M8S2	-	Eukaryotic translation initiation factor 4 gamma 3	-5.944273333	0.016240351	Down	1.77E-08
A0A8C0TT51	TSPAN9	Tetraspanin	-5.879506667	0.01698604	Down	5.015E-06
A0A8P0NAZ9	GMPS	GMP synthase (glutamine-hydrolyzing)	-5.742236667	0.018681619	Down	4.839E-08
A0A8I3P2H8	-	Gp_dh_N domain-containing protein	-5.703763333	0.019186517	Down	1.531E-06
A0A8I3N619	EIF2B4	Eukaryotic translation initiation factor 2B subunit delta	-5.700373333	0.019231654	Down	2.35E-07

A0A8P0SCX6	PDE12	Phosphodiesterase 12	-5.63504	0.020122593	Down	3.324E-08
A0A8I3S2F2	APP	Amyloid-beta A4 protein	-5.629623333	0.020198286	Down	5.449E-06
A0A8I3PBB6	KIF23	Kinesin-like protein	-5.568656667	0.021070134	Down	1.421E-07
A0A8C0P9K6	CLASP1	Cytoplasmic linker associated protein 1	-5.47702	0.022451879	Down	3.174E-08
A0A8C0TLC0	-	Laminin subunit alpha 5	-5.384843333	0.023933193	Down	1.362E-08
A0A8I3NC39	RBM4B	RNA binding motif protein 4B	-5.24108	0.02644099	Down	7.631E-08
A0A8C0RLN9	-	Ubiquitin carboxyl-terminal hydrolase	-5.207463333	0.027064333	Down	1.405E-09
A0A8C0SM51	CACNA1G	Acyl-CoA synthetase family member 2	-5.18174	0.02755122	Down	3.419E-08
A0A8I3S5F5	TNKS1BP1	Tankyrase 1 binding protein 1	-5.17595	0.027662014	Down	2.086E-08
A0A8C0PWD5	-	Serine-threonine kinase receptor-associated protein	-5.09168	0.029325916	Down	1.366E-06
A0A8C0RSJ7	-	DNA polymerase epsilon catalytic subunit	-5.077323333	0.029619204	Down	4.044E-07
A0A8I3N1K7	FHOD1	Formin homology 2 domain containing 1	-5.057763333	0.030023514	Down	1.973E-06
A0A8C0SYM7	-	IF rod domain-containing protein	-5.010923333	0.031014285	Down	2.166E-07
A0A8I3PQ61	ACOT7	Acyl-CoA thioesterase 7	-4.95141	0.032320425	Down	4.217E-08
A0A8C0TQ14	SEC23B	Protein transport protein SEC23	-4.94051	0.032565541	Down	2.558E-07
A0A8I3Q407	LOC477441	Glyceraldehyde-3-phosphate dehydrogenase	-4.92871	0.032832991	Down	1.357E-07
A0A8C0S7T2	TSPAN8	Tetraspanin	-4.899773333	0.033498183	Down	1.027E-08
A0A8I3NPL6	POLDIP3	DNA polymerase delta interacting protein 3	-4.873673333	0.034109719	Down	9.26E-07
A0A8C0PSB6	EHD2	EH domain containing 2	-4.86556	0.034302083	Down	2.239E-06
A0A8C0P373	-	MAC-inhibitory protein	-4.802586667	0.03583252	Down	6.507E-06
A0A8I3QQ41	CAMK2D	calcium/calmodulin-dependent protein kinase	-4.757266667	0.036976009	Down	1.094E-07
A0A8C0SR74	SYNJ2BP	PDZ domain-containing protein	-4.75308	0.037083469	Down	1.366E-05
A0A8I3PBM2	SNRPE	Small nuclear ribonucleoprotein E	-4.70278	0.038399198	Down	7.824E-08

A0A8C0REI4	FBLN1	Fibulin-1	-4.658143333	0.039605831	Down	1.888E-05
A0A8I3PU95	PDCD11	Programmed cell death 11	-4.65083	0.039807111	Down	3.551E-08
A0A8I3QAR9	PRDX4	Peroxiredoxin 4	-4.62996	0.040387146	Down	1.84E-06
A0A8C0SKH6	FLNC	Filamin C	-4.580006667	0.041810043	Down	2.229E-08
A0A8C0NDH6	-	Nucleoporin 214	-4.555206667	0.042534973	Down	4.86E-08
A0A8C0NL97	-	MAP7 domain containing 3	-4.482386667	0.044737031	Down	6.179E-06
A0A8P0TQW3	UBA1	E1 ubiquitin-activating enzyme	-4.47088	0.045095272	Down	1.295E-07
A0A8C0SL46	-	ATP binding cassette subfamily F member 1	-4.453396667	0.045645085	Down	4.161E-06
A0A8C0SGM7	RHOC	Ras homolog family member C	-4.429796667	0.0463979	Down	1.397E-06
A0A8C0TJ66	-	Solute carrier family 35 member G1	-4.380193333	0.048020914	Down	8.22E-06
A0A8I3P8Y7	MRPS23	Mitochondrial ribosomal protein S23	-4.359833333	0.048703412	Down	3.064E-07
A0A8P0SHD1	RBBP7	RB binding protein 7, chromatin remodeling factor	-4.320926667	0.050034719	Down	3.814E-09
A0A8I3NTV5	GTF2F1	Transcription initiation factor IIF subunit alpha	-4.299323333	0.050789591	Down	1.533E-06
A0A8I3QIS0	FYTTD1	UAP56-interacting factor	-4.2928	0.051019763	Down	4.602E-07
A0A8I3PUK3	ATAD3A	ATPase family AAA domain containing 3A	-4.28507	0.051293862	Down	9.143E-07
A0A8C0TF45	HCFC2	Host cell factor C2	-4.18088	0.055135296	Down	5.538E-07
A0A8P0SCT7	MAST4	non-specific serine/threonine protein kinase	-4.174333333	0.055386058	Down	1.076E-07
A0A8C0RG79	PARP3	Poly [ADP-ribose] polymerase	-4.145963333	0.056485981	Down	4.714E-07
A0A8I3MM14	S100A4	Protein S100	-4.137706667	0.056810182	Down	2.24E-07
A0A8C0YZ82	PKM	Multifunctional fusion protein	-4.115466667	0.05769273	Down	5.186E-07
A0A8I3MJ05	ZNF782	Zinc finger protein 782	-4.046923333	0.060499904	Down	3.665E-06

A0A8I3NGT5	ASF1A	Anti-silencing function 1A histone chaperone	-4.046433333	0.060520456	Down	8.476E-09
A0A8I3MEU9	CLCN6	Chloride channel protein	-4.02358	0.061486777	Down	3.803E-05
A0A8C0SYP8	-	PDZ binding kinase	-3.997746667	0.062597695	Down	2.497E-07
A0A8C0NH48	-	Arachidonate 15-lipoxygenase B	-3.986866667	0.063071556	Down	1.174E-06
A0A8I3MK84	TCP1	T-complex protein 1 subunit alpha	-3.979893333	0.063377153	Down	1.107E-06
A0A8I3RTV4	FBLIM1	Filamin binding LIM protein 1	-3.97837	0.063444108	Down	2.135E-06
A0A8C0TZ50	H1FX	H1 histone family, member X	-3.971023333	0.06376801	Down	4.663E-09
A0A8C0RCB6	SLC37A4	Solute carrier family 37 member 4	-3.905546667	0.066728798	Down	2.171E-07
A0A8C0MVL4	-	GTP_EFTU_D3 domain-containing protein	-3.90315	0.066839743	Down	4.654E-07
A0A8I3RTI9	POLR2L	DNA-directed RNA polymerases I, II, and III subunit RPABC5	-3.89036	0.067434935	Down	7.448E-07
A0A8I3N7M2	HYCC1	Family with sequence similarity 126 member A	-3.870936667	0.068348967	Down	4.136E-07
A0A8C0QGB2	-	Exportin 5	-3.843253333	0.069673153	Down	1.233E-06
A0A8C0M7D0	-	U3 small nucleolar ribonucleoprotein protein MPP10	-3.82685	0.070469852	Down	1.322E-08
A0A8I3MIJ1	PAIP1	Poly(A) binding protein interacting protein 1	-3.826296667	0.070496886	Down	4.483E-06
A0A8P0SWL1	USP15	Ubiquitin carboxyl-terminal hydrolase	-3.79036	0.072274974	Down	6.098E-07
A0A8I3S5X1	PRKAR2B	Protein kinase cAMP-dependent type II regulatory subunit beta	-3.782526667	0.07266847	Down	1.336E-07
A0A8I3MQ00	DDX19A	RNA helicase	-3.758236667	0.073902313	Down	2.17E-07
A0A8C0SF90	SLMAP	Sarcolemma associated protein	-3.720123333	0.075880693	Down	1.643E-07
A0A8C0NA31	KLC4	Kinesin light chain	-3.711426667	0.076339489	Down	1.166E-06
A0A8C0YYK6	-	Neutral cholesterol ester hydrolase 1	-3.637883333	0.080331892	Down	1.43E-07
A0A8I3M	KIF1B	Kinesin-like protein KIF1B	-	0.08387	Down	3.499

MD5			3.5756966 67	0271		E-08
A0A8C0 RFE4	CALU	Calumenin	- 3.5725866 67	0.08405 1264	Down	3.147 E-08
A0A8C0 MXK9	RASSF1	Ras association domain family member 1	- 3.5555633 33	0.08504 8916	Down	2.743 E-07
A0A8P0S NB9	BGN	Biglycan	- 3.5487133 33	0.08545 3694	Down	1.176 E-05
A0A8C0S 9V3	PRAF2	PRA1 family protein	- 3.5381633 33	0.08608 0882	Down	2.303 E-08
A0A8C0 RG40	LOC4787 01	Tubulin beta chain	-3.53615	0.08620 1095	Down	2.649 E-06
A0A8C0 Q2Y6	VWA1	von Willebrand factor A domain-containing protein 1	-3.49552	0.08866 3247	Down	5.177 E-06
A0A8P0S IT2	SAV1	Salvador family WW domain containing protein 1	-3.47293	0.09006 2479	Down	2.446 E-06
A0A8I3Q 414	ADD3	Adducin 3	- 3.4623033 33	0.09072 8314	Down	5.57E-07
A0A8C0Z 504	-	Metalloendopeptidase	- 3.4533433 33	0.09129 3545	Down	5.351 E-07
A0A8C0T Y64	CREB1	cAMP responsive element binding protein 1	- 3.4426833 33	0.09197 0607	Down	1.227 E-07
A0A8C0T 5H5	SH3BGR L	SH3 domain-binding glutamic acid-rich-like protein	- 3.4372933 33	0.09231 4857	Down	7.007 E-08
Q9GKL5	ACTC	Cardiac actin (Fragment)	- 3.4364433 33	0.09236 9263	Down	8.138 E-07
Q28294	GNAQ	Guanine nucleotide-binding protein G(q) subunit alpha	- 3.4262166 67	0.09302 6357	Down	3.751 E-08
A0A8C0P R31	HAPLN3	Hyaluronan and proteoglycan link protein 3	-3.42267	0.09325 533	Down	4.824 E-08
A0A8I3M SS4	CWF19L 2	CWF19 like cell cycle control factor 2	- 3.4041733 33	0.09445 8646	Down	5.774 E-09
A0A8C0R VU3	EPN2	Epsin 2	- 3.3623133 33	0.09723 9526	Down	4.925 E-06
A0A8I3M UK8	SYNE1	Spectrin repeat containing nuclear envelope protein 1	- 3.3137533 33	0.10056 824	Down	2.56E-09
A0A8I3P 614	STC1	Stanniocalcin 1	- 3.3135033 33	0.10058 5668	Down	1.254 E-06
A0A8C0 RCN8	VASN	Vasorin	- 3.3134566 67	0.10058 8922	Down	8.17E-07
A0A8C0T 3D0	-	SERPIN domain-containing protein	-3.30789	0.10097 7796	Down	1.549 E-07

A0A8P0P0X4	TAF1C	TATA-box binding protein associated factor, RNA polymerase I subunit C	-3.288383333	0.102352388	Down	1.007E-08
A0A8I3QL49	LOC610636	Tubulin alpha chain	-3.279203333	0.103005742	Down	2.754E-06
A0A8C0PVE4	RABIF	RAB interacting factor	-3.262903333	0.104176131	Down	7.881E-07
A0A8P0P367	ANO8	Anoctamin	-3.24203	0.105694338	Down	5.256E-07
A0A8C0Z2P1	GNB4	G protein subunit beta 4	-3.24194	0.105700932	Down	1.176E-06
A0A8C0TE86	-	Caspase 7	-3.231153333	0.106494193	Down	3.482E-06
A0A8C0NR02	-	Transmembrane protein 192	-3.216776667	0.107560728	Down	2.751E-08
A0A8C0MQL2	ANKRD50	Ankyrin repeat domain 50	-3.205613333	0.108396244	Down	1.339E-07
A0A8P0SEJ9	NXPE3	NXPE family member 3	-3.190936667	0.109504596	Down	6.316E-06
E2R6K5	H3-3A	Histone H3	-3.168843333	0.111194448	Down	2.678E-07
A0A8P0SVS6	-	ATP synthase subunit d, mitochondrial	-3.151166667	0.112565243	Down	3.404E-07
A0A8P0N4D2	LEMD2	LEM domain nuclear envelope protein 2	-3.124813333	0.114640337	Down	3.956E-07
A0A8C0Z1M4	LPP	LIM domain containing preferred translocation partner in lipoma	-3.120783333	0.11496102	Down	4.022E-07
A0A8I3PHI4	FN1	Fibronectin	-3.100883333	0.116557736	Down	4.103E-06
A0A8I3PDD8	POMT1	Dolichyl-phosphate-mannose--protein mannosyltransferase	-3.08265	0.118040186	Down	1.479E-06
A0A8C0Z4N4	HCK	Tyrosine-protein kinase	-3.078016667	0.118419891	Down	3.542E-08
A0A8I3MI21	AKAP7	A-kinase anchoring protein 7	-3.07584	0.118598692	Down	6.469E-06
A0A8C0M639	-	Sprouty RTK signaling antagonist 4	-3.066173333	0.119396021	Down	1.007E-05
A0A8C0RF81	TRIM3	Tripartite motif containing 3	-3.051303333	0.120633012	Down	1.121E-06
A0A8I3MC90	ZNF787	Zinc finger protein 787	-3.04644	0.121040353	Down	4.03E-08
A0A8C0S	IFRD1	Interferon related	-3.00319	0.12472	Down	3.276

R51		developmental regulator 1		3913		E-08
A0A8I3S915	IGFBP5	Insulin-like growth factor-binding protein 5	-2.99127	0.12575869	Down	5.197E-08
A0A8C0NP98	MCM10	Protein MCM10 homolog	-2.97454	0.127225521	Down	1.077E-06
A0A8I3PPH3	DTNBP1	Dystrobrevin binding protein 1	-2.972956667	0.127365225	Down	1.226E-07
A0A8C0QKW2	-	V-type proton ATPase subunit G	-2.96534	0.128039424	Down	1.298E-06
A0A8C0MC85	-	Inverted formin, FH2 and WH2 domain containing	-2.930023333	0.131212463	Down	1.979E-07
A0A8C0SP86	-	IQ motif and Sec7 domain ArfGEF 1	-2.905573333	0.13345513	Down	1.137E-06
A0A8C0MLZ0	-	Caldesmon 1	-2.903966667	0.133603836	Down	1.317E-06
A0A8C0TN66	-	Peptidyl-prolyl cis-trans isomerase	-2.894683333	0.13446631	Down	1.631E-07
A0A8C0MN92	CAPZB	F-actin-capping protein subunit beta	-2.89324	0.134600904	Down	2.578E-07
A0A8C0N9S3	-	Collagen type XII alpha 1 chain	-2.892573333	0.134663117	Down	5.496E-08
A0A8C0NEI1	-	Transmembrane protein 245	-2.88966	0.134935326	Down	5.08E-07
A0A8P0NPP3	COL15A1	Collagen type XV alpha 1 chain	-2.878196667	0.136011763	Down	1.727E-05
A0A8I3PJ74	WNT7A	Protein Wnt	-2.843416667	0.139330531	Down	2.923E-05
A0A8C0SRB4	FAM177A1	Family with sequence similarity 177 member A1	-2.8363	0.140019533	Down	5.005E-07
A0A8I3PUS2	GSPT2	G1 to S phase transition 2	-2.82132	0.141480978	Down	2.034E-07
A0A8C0Z2H0	-	CDC like kinase 3	-2.820026667	0.141607868	Down	3.925E-05
A0A8I3PEB2	SELENBP1	Methanethiol oxidase	-2.81361	0.142239099	Down	7.301E-08
A0A8I3QJC5	LEPROT	Leptin receptor overlapping transcript	-2.780633333	0.145527799	Down	3.059E-05
A0A8C0RTC0	-	Family with sequence similarity 210 member B	-2.766293333	0.146981518	Down	6.261E-06
A0A8C0MBD9	PLOD3	procollagen-lysine 5-dioxygenase	-2.757953333	0.147833657	Down	5.297E-07
A0A8C0TJ68	ID1	Inhibitor of DNA binding 1, HLH protein	-2.753056667	0.148336273	Down	5.095E-07
A0A8P0S	CENPV	Centromere protein V	-	0.14948	Down	1.114

LX2			2.741903333	7491		E-08
A0A8C0TLK4	PLA2G3	Phospholipase A2 group III	-2.736216667	0.150077888	Down	0.0001244
A0A8I3PFB1	TSR3	18S rRNA aminocarboxypropyltransferase	-2.726723333	0.151068698	Down	1.398E-07
A0A8I3QC34	RAD51AP1	RAD51 associated protein 1	-2.703043333	0.15356876	Down	5.429E-07
A0A8C0T6H9	-	NAD(P)H quinone dehydrogenase 1	-2.69687	0.154227293	Down	6.606E-06
A0A8C0N839	-	Transcription elongation factor 1 homolog	-2.690923333	0.154864317	Down	1.19E-05
A0A8C0MTI6	ARL8B	ADP ribosylation factor like GTPase 8B	-2.67829	0.156226381	Down	3.553E-09
A0A8I3MCN0	REEP3	Receptor expression-enhancing protein	-2.67504	0.156578713	Down	1.238E-05
A0A8I3NRN2	GCH1	GTP cyclohydrolase 1	-2.656626667	0.158589959	Down	1.097E-05
A0A8C0MLR6	-	Caldesmon 1	-2.65658	0.158595089	Down	2.553E-07
A0A8C0N8D2	-	MAF bZIP transcription factor F	-2.640823333	0.160336708	Down	3.841E-06
A0A8C0SN20	-	Decapping nuclease	-2.63619	0.160852471	Down	1.793E-06
A0A8I3MTN8	DERL2	Derlin	-2.61404	0.163341128	Down	5.863E-07
A0A8C0S876	PM20D2	Xaa-Arg dipeptidase	-2.610846667	0.163703076	Down	3.54E-06
A0A8C0N0M8	GABARAP	GABA type A receptor-associated protein	-2.608483333	0.163971464	Down	5.726E-06
A0A8C0SLW3	MAP3K20	Mitogen-activated protein kinase kinase kinase 20	-2.599166667	0.165033789	Down	3.488E-06
A0A8C0SB04	SERPINE1	SERPIN domain-containing protein	-2.589843333	0.166103763	Down	9.496E-06
A0A8C0QHW2	YIPF5	Yip1 domain family member 5	-2.579276667	0.167324816	Down	2.104E-06
A0A8C0Q7V6	SDHAF2	Succinate dehydrogenase assembly factor 2, mitochondrial	-2.567596667	0.168684969	Down	2.07E-05
A0A8C0P819	DCAF6	DDB1 and CUL4 associated factor 6	-2.56731	0.168718491	Down	3.984E-07
A0A8P0T8T4	UCHL1	Ubiquitin carboxyl-terminal hydrolase isozyme L1	-2.55262	0.170445215	Down	6.359E-06
A0A8I3NIE3	RAB6A	RAB6A, member RAS onco family	-2.53844	0.172128751	Down	1.484E-05
A0A8P0S	CCDC88	Coiled-coil domain	-2.5294	0.17321	Down	4.984

8P0	A	containing 88A		0705		E-09
A0A8C0QBK4	KDELR3	ER lumen protein-retaining receptor	-2.521226667	0.174194784	Down	5.651E-07
A0A8I3NH78	ARL6IP6	ADP-ribosylation factor-like protein 6-interacting protein 6	-2.505956667	0.176048316	Down	0.0001233
A0A8I3P8S6	-	Gp_dh_C domain-containing protein	-2.492486667	0.177699722	Down	9.507E-06
A0A8C0LZN7	RAMAC	RNA guanine-7 methyltransferase activating subunit	-2.49179	0.177785553	Down	5.521E-07
A0A8C0MIB2	-	Rho guanine nucleotide exchange factor 40	-2.489386667	0.178081966	Down	2.185E-08
A0A8P0TU84	CBFB	Core-binding factor subunit beta	-2.479213333	0.17934217	Down	1.493E-08
A0A8P0N8H7	P4HB	Protein disulfide-isomerase	-2.4726	0.180166164	Down	6.699E-08
A0A8C0Q8W5	-	Centrosomal protein 170	-2.4672	0.180841789	Down	8.025E-08
P61105	RAB2A	Ras-related protein Rab-2A	-2.46667	0.180908237	Down	1.563E-06
A0A8C0P9C8	DDR2	receptor protein-tyrosine kinase	-2.459066667	0.181864181	Down	0.0004919
A0A8I3QCP6	RAP2C	Ras-related protein Rap-2	-2.45798	0.182001217	Down	1.257E-06
A0A8P0TNU0	PGM5	Phosphoglucomutase 5	-2.44688	0.183406923	Down	4.251E-07
A0A8P0N5J2	TGFBR1	receptor protein serine/threonine kinase	-2.44514	0.183628259	Down	1.938E-07
A0A8P0SP38	LRRC8C	Leucine rich repeat containing 8 VRAC subunit C	-2.432263333	0.185274555	Down	5.104E-06
A0A8C0T575	FKBP7	peptidylprolyl isomerase	-2.416086667	0.187363694	Down	2.119E-07
A0A8I3PMT1	LOC478670	LIM domain containing preferred translocation partner in lipoma	-2.414583333	0.187559035	Down	1.709E-05
A0A8I3PX70	ATP5MK	ATP synthase membrane subunit DAPIT, mitochondrial	-2.393606667	0.19030605	Down	1.048E-05
A0A8I3PLN9	ADH4	Alcohol dehydrogenase 4 (class II), pi polypeptide	-2.392723333	0.190422606	Down	9.624E-07
A0A8C0LXR1	C7H1orf21	Chromosome 7 C1orf21 homolog	-2.391803333	0.190544077	Down	1.644E-05
A0A8C0SIR0	SLC30A6	Solute carrier family 30 member 6	-2.360296667	0.194751093	Down	1.269E-05
A0A8I3P7H7	FSTL1	Follistatin like 1	-2.3598066	0.19481725	Down	1.214E-06

			67			
A0A8I3N0K5	HSD17B6	Hydroxysteroid 17-beta dehydrogenase 6	-2.359516667	0.194856415	Down	1.222E-07
A0A8I3Q5U3	NEDD4	E3 ubiquitin-protein ligase	-2.356283333	0.195293612	Down	5.352E-07
A0A8C0STQ8	DYNLT3	Dynein light chain Tctex-type 3	-2.343416667	0.197043127	Down	1.291E-06
A0A8I3MV47	SIRT6	Sirtuin 6	-2.341013333	0.197371648	Down	1.7E-06
A0A8I3NX95	LCAT	Solute carrier family 12 member 4	-2.339866667	0.197528583	Down	9.924E-08
A0A8C0M169	-	RS14 protein	-2.33846	0.197721272	Down	3.271E-08
A0A8C0RDD6	TAGLN	Transgelin	-2.333516667	0.198399918	Down	2.487E-06
A0A8C0TFS1	GLO1	Lactoylglutathione lyase	-2.332596667	0.198526477	Down	4.901E-06
A0A8C0N7D3	SLC4A1AP	39S ribosomal protein L33, mitochondrial	-2.322503333	0.199920271	Down	2.269E-07
A0A8C0R9M3	-	Adipogenesis regulatory factor	-2.312643333	0.201291292	Down	2.201E-05
A0A8I3P8S4	RPS27L	40S ribosomal protein S27	-2.31047	0.201594754	Down	0.001289
A0A8P0TH65	ERO1B	Endoplasmic reticulum oxidoreductase 1 beta	-2.28688	0.204918196	Down	3.443E-06
A0A8I3PML6	STARD3NL	STARD3 N-terminal like	-2.276836667	0.206349712	Down	1.111E-06
A0A8I3QKT0	TCF12	Transcription factor 12	-2.27493	0.206622605	Down	9.017E-06
A0A8C0PJ67	-	DEAH-box helicase 32 (putative)	-2.26524	0.208015076	Down	3.768E-07
A0A8I3RTL0	UGDH	UDP-glucose 6-dehydrogenase	-2.25588	0.209369036	Down	1.702E-06
A0A8C0SA03	-	Transmembrane protein 189	-2.254206667	0.209612017	Down	2.043E-06
A0A8C0RQJ3	CMPK1	UMP-CMP kinase	-2.25232	0.209886314	Down	2.331E-05
A0A8I3N1F2	C1H19orf33	Collagen alpha-1(I) chain-like	-2.244586667	0.211014396	Down	2.922E-05
A0A8C0NCM2	FSCN1	Fascin actin-bundling protein 1	-2.232916667	0.21272822	Down	1.61E-06
A0A8C0M9E2	-	AE binding protein 1	-2.23018	0.21313213	Down	1.355E-05
A0A8I3N	ATP5F1D	ATP synthase F1 subunit	-2.23016	0.21313	Down	1.326

CZ3		delta		5084		E-06
A0A8I3P8Y6	TUBB2B	Tubulin beta chain	-2.226006667	0.213749557	Down	1.713E-05
A0A8C0MKE9	ATL2	Atlastin GTPase 2	-2.219246667	0.214753468	Down	1.133E-05
A0A8C0NDP7	-	Superoxide dismutase [Cu-Zn]	-2.20246	0.217266854	Down	1.016E-05
A0A8P0NF43	IGF2BP2	Insulin like growth factor 2 mRNA binding protein 2	-2.198593333	0.217849947	Down	2.119E-07
A0A8I3S1X1	ELMOD2	ELMO domain containing 2	-2.19705	0.218083118	Down	4.982E-06
A0A8C0NRU0	KDELR2	ER lumen protein-retaining receptor	-2.190063333	0.21914181	Down	8.74E-06
A0A8I3PCW6	LOC119865916	U6 snRNA-associated Sm-like protein LSm3	-2.186686667	0.219655318	Down	1.949E-07
A0A8C0T0G0	ABCD1	ATP binding cassette subfamily D member 1	-2.176183333	0.221260322	Down	4.038E-06
A0A8I3NN93	C13H8orf76	Chromosome 8 open reading frame 76	-2.172673333	0.221799292	Down	4.35E-05
A0A8P0TLV9	SERPINH1	Serpin H1	-2.170133333	0.222190135	Down	8.764E-07
A0A8I3NHI3	LEPROTL1	Leptin receptor overlapping transcript like 1	-2.167236667	0.2226367	Down	6.461E-05
A0A8P0SE84	GNAO1	G protein subunit alpha o1	-2.159913333	0.22376971	Down	3.492E-06
A0A8I3NN11	FUS	FUS RNA binding protein	-2.15636	0.22432153	Down	1.173E-07
A0A8P0T3P5	ALAS1	5-aminolevulinate synthase	-2.15485	0.22455644	Down	6.158E-07
A0A8I3MAV2	LMAN1	Lectin, mannose binding 1	-2.148473333	0.225551169	Down	1.932E-07
Q9XSB8	TPP1	Tripeptidyl-peptidase 1	-2.140113333	0.226861967	Down	3.394E-07
A0A8C0Q1G2	CRIP1	Cysteine rich protein 1	-2.136013333	0.227507603	Down	1.949E-05
A0A8I3PCD8	SEC62	Translocation protein SEC62	-2.13263	0.228041768	Down	2.235E-05
A0A8P0SL16	NES	Nestin	-2.130696667	0.228347569	Down	1.756E-09
A0A8C0N604	-	E3 ubiquitin-protein ligase	-2.129186667	0.228586694	Down	6.383E-05
A0A8C0	-	Receptor expression-	-2.1173	0.23047	Down	1.158

NRK5		enhancing protein		7848		E-06
A0A8C0TD88	FLNA	Filamin A	-2.11448	0.230928798	Down	8.464E-07
A0A8C0RT03	-	Erythrocyte membrane protein band 4.1 like 1	-2.107513333	0.232046633	Down	3.304E-06
A0A8I3MW60	VGLL4	Vestigial like family member 4	-2.10023	0.233221064	Down	8.241E-06
A0A8I3SAX9	ARL2	ADP-ribosylation factor-like protein 2	-2.08441	0.235792543	Down	2.118E-06
A0A8I3MV24	NDUFS4	NADH dehydrogenase [ubiquinone] iron-sulfur protein 4, mitochondrial	-2.081813333	0.236217322	Down	1.323E-05
A0A8C0P4H6	-	KN motif and ankyrin repeat domains 2	-2.075633333	0.237231364	Down	3.462E-08
A0A8I3S603	DPY19L1	Dpy-19 like C-mannosyltransferase 1	-2.07322	0.237628536	Down	1.173E-06
A0A8I3PV68	CLPTM1L	CLPTM1 like	-2.071333333	0.237939495	Down	4.577E-07
A0A8I3PJK3	CHM	Rab proteins geranylgeranyltransferase component A	-2.070976667	0.237998326	Down	1.622E-06
A0A8C0PLU5	YTHDC1	YTH domain containing 1	-2.06988	0.23817931	Down	8.673E-06
A0A8I3PAF7	PLOD2	procollagen-lysine 5-dioxygenase	-2.064403333	0.239085189	Down	9.194E-07
A0A8I3PW19	IKBIP	IKBKB interacting protein	-2.060946667	0.239658719	Down	1.443E-06
A0A8C0TBV2	SLC38A2	Solute carrier family 38 member 2	-2.054626667	0.240710893	Down	5.616E-07
A0A8C0PW39	SLC9A3R2	Na(+)/H(+) exchange regulatory cofactor NHE-RF	-2.050413333	0.241414907	Down	4.546E-07
A0A8I3S2J8	LOC119873296	START domain-containing protein	-2.048453333	0.241743108	Down	0.0004241
A0A8C0RRP5	TGFB2	Transforming growth factor beta	-2.040883333	0.243014898	Down	1.366E-07
A0A8C0T658	DYNC2H1	Cytoplasmic dynein 2 heavy chain 1	-2.04035	0.243104752	Down	4.515E-06
A0A8I3N737	APBB1IP	Amyloid beta precursor protein binding family B member 1 interacting protein	-2.03151	0.24459893	Down	4.37E-06
A0A8C0MPW7	GALNT10	Polypeptide N-acetylgalactosaminyltransferase	-2.019406667	0.246659598	Down	2.523E-05
A0A8C0NRI7	-	Acetoacetyl-CoA synthetase	-2.01534	0.247355863	Down	2.233E-05
A0A8C0Q0X5	RPS7	40S ribosomal protein S7	-2.0071266	0.248768088	Down	1.075E-06

			67			
A0A8I3N059	LOC102153215	Fatty acid hydroxylase domain-containing protein	-2.000956667	0.249834277	Down	2.266E-06
A0A8P0NDY9	C4H1orf198	Chromosome 4 C1orf198 homolog	-2.000786667	0.249863718	Down	1.774E-06
A0A8C0N9G5	UBQLN2	Ubiquilin 2	-2.000296667	0.249948597	Down	1.314E-05
A0A8C0SAG8	ACTN4	Actinin alpha 4	-2	0.25	Down	4.485E-07
A0A8I3Q3L7	FAM3A	ILEI domain-containing protein	-1.997803333	0.250380943	Down	3.17E-06
A0A8C0M833	ROGDI	Protein rogdi homolog	-1.992753333	0.251258911	Down	8.611E-07
A0A8C0SKF0	-	Chromosome 18 open reading frame 32	-1.98835	0.252026964	Down	1.075E-05
A0A8I3PG90	SPC24	Kinetochore protein Spc24	-1.982246667	0.253095425	Down	4.789E-06
A0A8I3Q238	DUSP12	protein-tyrosine-phosphatase	-1.981993333	0.253139872	Down	0.0001129
Q863Z4	MTPN	Myotrophin	-1.976066667	0.254181922	Down	2.46E-05
A0A8C0SL72	VMA21	Vacuolar ATPase assembly integral membrane protein VMA21	-1.970756667	0.255119191	Down	2.613E-06
A0A8I3NVB0	ZYX	Zyxin	-1.96879	0.255467204	Down	8.911E-06
A0A8I3SCI0	PCYT1A	choline-phosphate cytidylyltransferase	-1.968423333	0.25553214	Down	1.531E-06
A0A8I3MJP8	SEC23A	Protein transport protein SEC23	-1.968293333	0.255555167	Down	6.872E-07
A0A8I3S322	CCNYL1	Cyclin Y like 1	-1.96709	0.255768411	Down	1.177E-05
A0A8C0Z329	ALDOC	Fructose-bisphosphate aldolase	-1.96705	0.255775503	Down	3.969E-05
A0A8C0NZL5	CD70	CD70 molecule	-1.96316	0.256466092	Down	0.0010583
A0A8C0PH00	RPS6KA3	Ribosomal protein S6 kinase	-1.950636667	0.25870204	Down	4.301E-06
A0A8I3PQK0	KRAS	KRAS proto-onco, GTPase	-1.9504	0.258744482	Down	1.743E-06
A0A8C0S290	-	TLE family member 2, transcriptional corepressor	-1.947973333	0.259180066	Down	7.271E-07
A0A8C0TB79	-	Coatomer subunit zeta	-1.946266667	0.25948685	Down	3.902E-06

A0A8C0YYZ5	-	Protein phosphatase 1 regulatory subunit 18	-1.944716667	0.259765787	Down	1.142E-08
A0A8I3PEJ9	RRAS	RAS related	-1.942186667	0.260221728	Down	4.328E-07
A0A8I3NYQ2	P3H1	procollagen-proline 3-dioxygenase	-1.941406667	0.260362456	Down	6.203E-06
A0A8I3S7R6	SLC38A5	Solute carrier family 38 member 5	-1.940663333	0.26049664	Down	1.804E-06
A0A8I3PZA2	URM1	Ubiquitin-related modifier 1	-1.939313333	0.260740513	Down	3.666E-06
A0A8C0P9Y8	LACC1	Laccase domain containing 1	-1.93667	0.261218685	Down	3.057E-07
A0A8I3PAX0	FXR1	FMR1 autosomal homolog 1	-1.936633333	0.261225324	Down	8.873E-07
A0A8C0Q634	FN3KRP	protein-ribulosamine 3-kinase	-1.928886667	0.262631767	Down	6.019E-05
A0A8P0SLN2	LDLR	Low density lipoprotein receptor	-1.928706667	0.262664536	Down	8.132E-06
A0A8C0N8E3	VIM	Vimentin	-1.925333333	0.263279421	Down	2.013E-06
A0A8C0QFJ2	-	Cytoskeleton associated protein 4	-1.92498	0.26334391	Down	1.036E-06
A0A8C0SL00	FNDC3B	Fibronectin type III domain containing 3B	-1.919186667	0.264403528	Down	3.614E-06
A0A8C0PR28	FDPS	Farnesyl diphosphate synthase	-1.915696667	0.265043917	Down	4.045E-06
A0A8C0M9K4	AK4	Adenylate kinase 4, mitochondrial	-1.914076667	0.265341701	Down	1.913E-07
A0A8I3MSC7	PGM2	Phosphoglucomutase 2	-1.91307	0.265526913	Down	6.229E-06
A0A8C0SLX4	PHPT1	14 kDa phosphohistidine phosphatase	-1.910646667	0.2659733	Down	1.991E-05
A0A8I3PFK5	FAR2	Fatty acyl-CoA reductase	-1.907046667	0.26663782	Down	2.594E-05
A0A8C0N7I0	-	UBC core domain-containing protein	-1.90521	0.266977488	Down	6.336E-06
A0A8I3NHU6	ZFP90	ZFP90 zinc finger protein	-1.903813333	0.267236073	Down	9.274E-06
A0A8C0TAQ1	-	ADP-ribosylation factor-like protein 6	-1.902056667	0.267561665	Down	1.796E-06
A0A8I3Q	TPM4	RAB8A, member RAS	-1.8961	0.26866	Down	2.164

4E9		onco family		867		E-08
A0A8C0T5I9	RRAS2	RAS related 2	-1.895556667	0.268769872	Down	1.843E-06
A0A8C0M5C1	-	CSRP2 protein	-1.892443333	0.269350503	Down	6.985E-06
A0A8I3P398	GBP6	GB1/RHD3-type G domain-containing protein	-1.88888	0.270016598	Down	1.407E-05
A0A8P0NZ06	TMX2	Thioredoxin-related transmembrane protein 2	-1.879356667	0.271804893	Down	9.971E-06
Q711A6	HMGN2	Non-histone chromosomal protein HMG-17	-1.876166667	0.272406557	Down	4.372E-07
A0A8I3MRI3	MBOAT7	Membrane bound O-acyltransferase domain containing 7	-1.873643333	0.272883424	Down	6.911E-05
A0A8C0T8G5	-	CCDC50_N domain-containing protein	-1.87316	0.272974861	Down	2.371E-06
A0A8I3S8V0	CCN1	Cellular communication network factor 1	-1.865923333	0.274347563	Down	2.482E-05
A0A8C0PU47	UBAP2L	Ubiquitin associated protein 2 like	-1.865016667	0.274520032	Down	3.991E-06
A0A8I3PIU3	PXK	PX domain containing serine/threonine kinase like	-1.86053	0.275375096	Down	4.217E-05
A0A8C0N6A9	-	Mannosyltransferase	-1.855643333	0.276309423	Down	4.071E-06
A0A8C0LYM6	ARPC5	Actin-related protein 2/3 complex subunit 5	-1.852393333	0.276932574	Down	1.404E-06
A0A8I3MW03	-	ATP_bind_3 domain-containing protein	-1.848933333	0.277597536	Down	5.543E-06
A0A8C0QFA0	-	Chromatin accessibility complex subunit 1	-1.846596667	0.278047512	Down	3.702E-07
A0A8C0PAV5	-	39S ribosomal protein L21, mitochondrial	-1.843023333	0.278737047	Down	9.326E-07
A0A8P0NW03	ARHGAP19	Rho-GAP domain-containing protein	-1.83857	0.279598786	Down	1.175E-05
A0A8C0YXD9	-	Oligosaccharyltransferase complex subunit	-1.83736	0.279833386	Down	3.421E-07
A0A8C0NP94	PC	Pyruvate carboxylase	-1.836573333	0.279986014	Down	1.021E-06
A0A8I3N1J6	RPL36AL	Ribosomal protein L36a like	-1.83327	0.280627831	Down	8.308E-06
A0A8I3N4M9	-	Transcription initiation factor TFIID subunit 12	-1.830603333	0.281147021	Down	4.512E-05
A0A8I3PXS4	-	Methyl-CpG-binding protein 2	-1.8275033	0.281751787	Down	7.501E-07

			33			
A0A8C0NHL4	PROSC	Pyridoxal phosphate homeostasis protein	-1.823913333	0.282453771	Down	1.162E-06
A0A8C0MEN9	QSOX1	Sulfhydryl oxidase	-1.822573333	0.282716241	Down	4.685E-06
A0A8C0SQQ0	MYO9A	Myosin IXA	-1.817806667	0.28365188	Down	9.609E-06
A0A8I3NC31	DOHH	Deoxyhypusine hydroxylase	-1.816666667	0.283876107	Down	1.135E-05
A0A8C0Z1X3	DPYSL2	Dihydropyrimidinase-related protein 2	-1.813983333	0.284404593	Down	1.402E-05
A0A8I3P5J3	TFCP2	Transcription factor CP2	-1.811053333	0.284982783	Down	5.245E-06
A0A8I3P342	ISG15	ISG15 ubiquitin like modifier	-1.803873333	0.286404619	Down	1.188E-06
A0A8I3RU99	COMMD3	COMM domain-containing protein 3	-1.800853333	0.287004779	Down	9.148E-07
A0A8I3NME6	C5orf15	Chromosome 5 open reading frame 15	-1.797576667	0.287657369	Down	3.112E-06
A0A8P0SXZ1	CD276	CD276 molecule	-1.793163333	0.288538686	Down	2.251E-06
A0A8C0RIP9	BUD23	BUD23 rRNA methyltransferase and ribosome maturation factor	-1.792883333	0.288594691	Down	4.137E-07
A0A8C0RKE6	PDF	Peptide deformylase	-1.79158	0.288855526	Down	2.431E-06
A0A8C0SSL4	-	GLOBIN domain-containing protein	-1.79027	0.289117933	Down	6.366E-05
A0A8C0LZF5	-	PVR cell adhesion molecule	-1.784913333	0.290193411	Down	3.604E-06
A0A8I3P6R9	MTFP1	Mitochondrial fission process protein 1	-1.780523333	0.29107779	Down	1.873E-06
A0A8C0RRN5	PGAM1	Phosphoglycerate mutase	-1.77171	0.292861408	Down	6.04E-05
A0A8I3PLQ8	CDK17	Cyclin dependent kinase 17	-1.770576667	0.293091561	Down	4.592E-06
A0A8I3PJ87	TUBB4B	Tubulin beta chain	-1.76819	0.293576827	Down	1.655E-05
A0A8C0MH41	MAPRE3	Microtubule associated protein RP/EB family member 3	-1.764556667	0.294317113	Down	2.275E-05
A0A8C0R9T3	CHMP2A	Charged multivesicular body protein 2A	-1.76384	0.294463353	Down	5.004E-06
A0A8I3P	GEMIN6	Gem nuclear organelle	-	0.29465	Down	6.498

LN7		associated protein 6	1.762916667	1871		E-06
A0A8P0SK90	NT5C3B	5'-nucleotidase	-1.761796667	0.294880706	Down	3.109E-06
A0A8I3Q1R0	RNF10	RING finger protein 10	-1.760873333	0.295069491	Down	2.74E-06
A0A8C0N0T1	RPSA	40S ribosomal protein SA	-1.756683333	0.295927703	Down	5.242E-06
A0A8I3Q9E7	UNC119B	Unc-119 lipid binding chaperone B	-1.75475	0.296324537	Down	1.314E-06
A0A8I3Q4K9	YRDC	Threonylcarbamoyl-AMP synthase	-1.753416667	0.296598526	Down	1.636E-06
A0A8I3PFV2	RSAD2	Radical S-adenosyl methionine domain containing 2	-1.753063333	0.296671175	Down	4.682E-06
A0A8C0NJ48	NDUFA12	NADH dehydrogenase [ubiquinone] 1 alpha subcomplex subunit 12	-1.752846667	0.296715733	Down	3.413E-07
A0A8C0PVH8	-	Arrestin beta 2	-1.74523	0.298286378	Down	1.167E-06
A0A8C0N5E1	SRSF6	Serine and arginine rich splicing factor 6	-1.74429	0.298480792	Down	1.677E-06
A0A8C0T7U2	-	Semaphorin-3C	-1.74263	0.298824429	Down	6.79E-06
A0A8C0SS63	DCAF7	DDB1 and CUL4 associated factor 7	-1.74162	0.299033703	Down	1.539E-07
A0A8C0PU06	-	CRK proto-oncogene, adaptor protein	-1.738626667	0.299654788	Down	4.553E-06
A0A8I3RUW4	CA9	Carbonic anhydrase	-1.73808	0.299768355	Down	5.136E-06
A0A8C0MAD0	TUBB6	Tubulin beta 6 class V	-1.736103333	0.300179356	Down	1.258E-05
A0A8C0NYE6	-	procollagen-proline 3-dioxygenase	-1.72913	0.301633799	Down	3.523E-06
A0A8C0PRJ3	WBP2	WW domain binding protein 2	-1.726226667	0.302241429	Down	3.063E-06
A0A8C0TH39	MTMR9	Myotubularin related protein 9	-1.725653333	0.302361565	Down	7.808E-06
A0A8C0LSL6	-	Plasminogen activator, urokinase receptor	-1.7231	0.302897169	Down	7.806E-05
A0A8I3S0W1	FAM98A	Family with sequence similarity 98 member A	-1.72265	0.302991662	Down	4.974E-07
A0A8C0TSW1	PDCD6	Programmed cell death 6	-1.721636667	0.303204555	Down	1.611E-05
A0A8C0S5M2	PGM1	Phosphoglucomutase 1	-1.71988	0.303573971	Down	5.719E-06
J9NYK7	MYADM	Myeloid associated differentiation marker	-1.7177933	0.304013368	Down	9.367E-07

			33			
A0A8I3Q4Q6	ANO6	Anoctamin	-1.711436667	0.305355838	Down	3.923E-06
A0A8I3PXJ3	RPL10	Ribosomal_L16 domain-containing protein	-1.707713333	0.306144924	Down	6.619E-06
A0A8I3RYG1	NAE1	NEDD8-activating enzyme E1 regulatory subunit	-1.70581	0.306549084	Down	5.95E-06
A0A8C0YYZ8	PAAF1	Proteasomal ATPase associated factor 1	-1.702503333	0.307252503	Down	5.081E-06
A0A8I3NIT3	RHOG	Ras homolog family member G	-1.699676667	0.307855091	Down	4.361E-06
A0A8C0M8E8	-	Serine protease 33	-1.698653333	0.308073537	Down	1.246E-05
A0A8C0MV70	-	Profilin	-1.690863333	0.309741515	Down	4.163E-05
A0A8C0Z0J5	TAF9B	TATA-box binding protein associated factor 9b	-1.688463333	0.310257215	Down	2.39E-06
A0A8I3PIG4	-	60S acidic ribosomal protein P0	-1.68775	0.310410658	Down	1.29E-05
A0A8I3NBM4	MALSU1	Mitochondrial assembly of ribosomal large subunit 1	-1.681126667	0.311839013	Down	5.951E-06
A0A8I3N3W5	PSMG3	Proteasome assembly chaperone 3	-1.67886	0.312329339	Down	7.096E-05
A0A8C0RV37	-	Surfeit locus protein 4	-1.673023333	0.313595479	Down	8.865E-07
A0A8C0MWB3	SMPD1	Sphingomyelin phosphodiesterase	-1.671643333	0.31389559	Down	2.471E-06
A0A8I3P0Z8	BAG2	BAG cochaperone 2	-1.670646667	0.314112516	Down	4.402E-07
A0A8I3N6E4	CRABP2	FABP domain-containing protein	-1.66502	0.315339981	Down	2.943E-05
A0A8C0PW63	AKAP10	A-kinase anchoring protein 10	-1.663716667	0.315624988	Down	1.262E-06
A0A8I3PWU6	MSN	Moesin	-1.663326667	0.315710322	Down	1.256E-05
A0A8C0RL91	ARHGAP10	Rho GTPase activating protein 10	-1.662063333	0.315986903	Down	0.0007577
A0A8C0NN71	CASC4	Protein GOLM2	-1.656123333	0.317290596	Down	2.148E-05
A0A8C0SBV4	PRMT7	Protein arginine N-methyltransferase	-1.65127	0.318359782	Down	1.696E-06
A0A8C0	TRMT2A	tRNA (uracil(54)-C(5))-	-1.64724	0.31925	Down	3.669

RNG0		methyltransferase		0326		E-07
A0A8I3Q1R6	PPP1CC	Serine/threonine-protein phosphatase	-1.64198	0.320416423	Down	7.906E-05
A0A8C0MJ40	-	Dihydropyrimidinase like 4	-1.641056667	0.320621557	Down	1.368E-05
A0A8C0YRT6	FAM20C	FAM20C golgi associated secretory pathway kinase	-1.638306667	0.321233294	Down	2.221E-05
A0A8C0SG12	-	phosphopyruvate hydratase	-1.63751	0.32141073	Down	5.413E-05
A0A8C0SLE6	-	DNA repair protein SWI5 homolog	-1.63599	0.321749542	Down	3.414E-05
A0A8C0TUX1	-	arginyltransferase	-1.635736667	0.321806045	Down	4.296E-06
A0A8I3Q1D6	RAN	GTP-binding nuclear protein Ran	-1.63395	0.322204824	Down	4.913E-06
A0A8C0RGG3	FUBP1	Far upstream element binding protein 1	-1.63386	0.322224925	Down	1.983E-05
A0A8C0LSF6	CSRP1	Cysteine and glycine rich protein 1	-1.632306667	0.322572048	Down	2.65E-05
A0A8C0MEQ2	ETHE1	ETHE1 persulfide dioxygenase	-1.62857	0.323408612	Down	6.507E-06
I7KJS3	JPT1	HN1	-1.628343333	0.323459427	Down	2.19E-05
A0A8I3S3S8	BIN1	Bridging integrator 1	-1.626256667	0.323927607	Down	1.878E-05
A0A8I3Q739	ACTBL2	Actin beta like 2	-1.62477	0.32426158	Down	3.165E-06
A0A8C0MG62	-	Sequestosome 1	-1.622466667	0.324779693	Down	1.309E-06
A0A8C0SV74	-	Solute carrier family 35 member E1	-1.620553333	0.325210708	Down	6.509E-06
A0A8C0S0E9	-	Chromosome 1 open reading frame 174	-1.6197	0.325403123	Down	3.611E-06
A0A8C0PC72	-	Calcium uniporter protein	-1.61948	0.325452748	Down	6.444E-06
A0A8C0Q5K6	MDH1	Malate dehydrogenase 1	-1.616116667	0.326212356	Down	4.392E-06
A0A8C0QLU6	-	Chromatin modification-related protein MEAF6	-1.61587	0.326268135	Down	1.519E-07
A0A8C0QHX9	COPB2	Coatomer subunit beta'	-1.607636667	0.328135442	Down	5.074E-06
A0A8I3NGI4	CAMLG	Guided entry of tail-anchored proteins factor	-1.60558	0.328603557	Down	3.241E-06
A0A8C0NBD6	-	Forkhead box K1	-1.60346	0.329086785	Down	1.272E-06
A0A6S4Q833	DLA88	DLA class I alpha chain	-1.5994266	0.330008098	Down	1.45E-06

			67			
A0A8C0RTL9	-	SWI/SNF related, matrix associated, actin dependent regulator of chromatin, subfamily d, member 1	-1.59877	0.330158341	Down	4.857E-06
A0A8I3PP03	IDH3G	Isocitrate dehydrogenase [NAD] subunit, mitochondrial	-1.5978	0.330380399	Down	9.067E-07
A0A8C0PKH9	-	Family with sequence similarity 160 member B1	-1.595213333	0.330973282	Down	3.031E-08
A0A8C0T4H2	SERPINB6	Serpin family B member 6	-1.594636667	0.331105604	Down	3.814E-05
A0A8P0N6B7	ADD2	Adducin 2	-1.594573333	0.33112014	Down	1E-06
A0A8C0NB58	-	Family with sequence similarity 107 member B	-1.593373333	0.331395672	Down	4.521E-05
A0A8I3RWV5	FOXRED1	FAD dependent oxidoreductase domain containing 1	-1.5926	0.331573359	Down	6.558E-06
A0A8I3N6H0	NENF	Neudesin neurotrophic factor	-1.592326667	0.331636185	Down	4.939E-07
A0A8I3SAD7	COLGALT1	Collagen beta(1-O)galactosyltransferase 1	-1.587636667	0.332716042	Down	1.883E-06
A0A8C0MA89	PAIP2	Poly(A) binding protein interacting protein 2	-1.586173333	0.333053689	Down	0.0002711
A0A8C0P433	-	Charged multivesicular body protein 7	-1.580513333	0.334362896	Down	7.671E-06
A0A8I3NFH7	CRLF3	Cytokine receptor like factor 3	-1.579133333	0.334682882	Down	0.0011153
A0A140KFQ2	DLA-88	MHC class I antigen alpha chain (Fragment)	-1.578933333	0.334729282	Down	0.0003993
A0A8I3RRE8	MVD	Diphosphomevalonate decarboxylase	-1.57769	0.33501788	Down	8.239E-06
A0A8I3ML59	LNPEP	Leucyl and cystinyl aminopeptidase	-1.57113	0.33654469	Down	5.704E-07
A0A8C0S206	SLC35A1	Solute carrier family 35 member A1	-1.569543333	0.336915023	Down	5.511E-06
A0A8C0NVE7	-	Enoyl-CoA hydratase domain containing 3	-1.569526667	0.336918916	Down	1.065E-06
A0A8P0SNQ2	TERF2IP	Telomeric repeat-binding factor 2-interacting protein 1	-1.56802	0.337270958	Down	3.209E-06
A0A8I3P9H2	CBX5	Chromobox 5	-1.565843333	0.3377802	Down	2.195E-06

A0A8C0SGM8	ZFP36L1	mRNA decay activator protein ZFP36	-1.565706667	0.337812199	Down	8.284E-08
A0A8C0YWB0	DLGAP4	DLG associated protein 4	-1.562576667	0.338545895	Down	1.539E-05
A0A8I3PUM4	NAA10	N-terminal amino-acid N(alpha)-acetyltransferase NatA	-1.560536667	0.339024945	Down	2.858E-05
A0A8C0SZN8	PGK1	Phosphoglycerate kinase	-1.557736667	0.339683567	Down	0.0001614
A0A8C0SKJ5	GNE	Glucosamine (UDP-N-acetyl)-2-epimerase/N-acetylmannosamine kinase	-1.555386667	0.340237328	Down	1.318E-06
A0A8I3Q8W7	-	Prefoldin subunit 4	-1.554826667	0.340369421	Down	1.426E-05
A0A8P0S737	EPB41L2	Erythrocyte membrane protein band 4.1 like 2	-1.55434	0.340484257	Down	4.109E-05
A0A8I3PK60	NFKB2	Nuclear factor kappa B subunit 2	-1.55398	0.34056923	Down	2.597E-06
A0A8C0MWR0	ATP5IF1	ATP synthase F1 subunit epsilon	-1.550716667	0.341340459	Down	3.598E-06
A0A8C0M2X0	QDPR	Quinoid dihydropteridine reductase	-1.547646667	0.342067592	Down	3.318E-06
A0A8I3P624	JPT2	Jupiter microtubule associated homolog 2	-1.54729	0.342152169	Down	7.757E-06
A0A8I3NVQ8	B3GNT2	Hexosyltransferase	-1.545063333	0.342680657	Down	5.761E-07
A0A8I3N184	LGALS1	Galectin	-1.542206667	0.343359868	Down	1.172E-05
A0A8I3NX87	LOC102155886	Serum amyloid A protein	-1.539303333	0.344051554	Down	4.4E-06
A0A8C0SCR3	LOC607874	Cystatin-C-like	-1.538696667	0.344196262	Down	0.0019783
A0A8P0PP54	RPS11	40S ribosomal protein S11	-1.53805	0.344350577	Down	8.817E-06
A0A8I3Q6M8	FAM8A1	Family with sequence similarity 8 member A1	-1.537583333	0.344461982	Down	0.0001299
A0A8C0TSA1	RMDN2	Regulator of microtubule dynamics 2	-1.53703	0.344594123	Down	1.12E-06
A0A8P0SKX9	UBQLN4	Ubiquilin 4	-1.534136667	0.345285902	Down	1.062E-06
A0A8C0MB63	-	Zinc finger and BTB domain containing 7A	-1.533703333	0.345389628	Down	2.528E-05
A0A8I3MKW3	GAPDHS	Glyceraldehyde-3-phosphate dehydrogenase	-1.5327366	0.345621132	Down	2.032E-05

			67			
A0A8I3Q5A3	BID	BH3-interacting domain death agonist	-1.52827	0.346692853	Down	6.51E-06
A0A8C0YX59	KLC1	Kinesin light chain	-1.527656667	0.346840274	Down	4.87E-06
A0A8C0NEU9	CSNK1E	Casein kinase 1 epsilon	-1.52759	0.346856301	Down	9.011E-07
A0A8C0NW72	-	TWF1	-1.527096667	0.34697493	Down	2.188E-05
A0A8C0Q131	-	Secretion associated Ras related GTPase 1A	-1.526576667	0.347100015	Down	2.417E-06
A0A8P0N4Y2	RIGI	RNA helicase	-1.523213333	0.347910148	Down	6.287E-06
A0A8C0NRQ3	FMNL3	Formin like 3	-1.521656667	0.348285746	Down	4.179E-08
A0A8P0P7K3	ANXA8L1	Annexin	-1.521453333	0.348334836	Down	2.367E-05
A0A8C0SM23	-	ArfGAP with FG repeats 2	-1.52077	0.348499864	Down	7.335E-06
A0A8I3QNJ1	VPS29	Vacuolar protein sorting-associated protein 29	-1.520466667	0.348573146	Down	1.283E-05
A0A8C0T6M0	-	Caveolae associated protein 3	-1.51897	0.348934947	Down	6.217E-08
D0VWQ5	RPL19	60S ribosomal protein L19	-1.518643333	0.349013964	Down	4.18E-06
A0A8I3RQU0	STX6	Syntaxin 6	-1.51514	0.349862514	Down	2.202E-06
A0A8I3MM03	HMGCS1	Hydroxymethylglutaryl-CoA synthase	-1.51377	0.350194905	Down	3.323E-05
A0A8C0TD66	-	3'-phosphoadenosine 5'-phosphosulfate synthase 1	-1.51201	0.350622382	Down	9.146E-07
A0A8P0SB92	NAV3	Neuron navigator 3	-1.511833333	0.350665321	Down	5.244E-06
A0A8C0TFK2	RAB34	RAB34, member RAS oncogene family	-1.509566667	0.351216696	Down	0.0045412
A0A8I3MRD6	NDUFA2	NADH dehydrogenase [ubiquinone] 1 alpha subcomplex subunit 2	-1.507926667	0.351616172	Down	2.092E-05
A0A8C0PEX9	-	Tensin 1	-1.507286667	0.351772189	Down	4.678E-07
A0A8C0NRZ9	AP2B1	AP complex subunit beta	-1.506563333	0.351948603	Down	4.196E-07
A0A8I3MCN2	FER	Tyrosine-protein kinase	-1.50577	0.352142192	Down	2.814E-06
A0A8C0	SQOR	Sulfide quinone	-1.49986	0.35358	Down	2.933

RIL1		oxidoreductase		7701		E-05
A0A8P0N 9M7	CYP7B1	Cytochrome P450 family 7 subfamily B member 1	-1.49948	0.35368 0847	Down	2.105 E-06
A0A8I3N W81	PSMA7	Proteasome subunit alpha type	- 1.4957566 67	0.35459 4812	Down	1.07E-05
A0A8C0S Q47	SS18	SS18 subunit of BAF chromatin remodeling complex	-1.49179	0.35557 1106	Down	4.313 E-06
A0A8C0 YWP4	HSD3B7	Hydroxy-delta-5-steroid dehydrogenase, 3 beta- and steroid delta-isomerase 7	-1.49085	0.35580 2857	Down	7.429 E-05
A0A8C0Z 3I7	RPL37A	Ribosomal protein L37a	-1.49076	0.35582 5054	Down	8.179 E-06
A0A8C0P 467	SLC25A1	Solute carrier family 25 member 1	- 1.4888133 33	0.35630 5502	Down	1.011 E-05
A0A8I3P SR9	GIGYF2	GYF domain-containing protein	-1.48826	0.35644 2186	Down	1.956 E-06
A0A8I3M NS2	TTC1	Tetratricopeptide repeat domain 1	-1.48789	0.35653 3613	Down	4.771 E-06
A0A8I3N GY9	BAG6	Large proline-rich protein BAG6	- 1.4870366 67	0.35674 456	Down	5.718 E-06
A0A8I3R RG1	HSPB6	Heat shock protein family B (small) member 6	-1.4846	0.35734 7599	Down	7.634 E-06
A0A8I3N S93	OSBP	Oxysterol-binding protein	-1.48451	0.35736 9892	Down	3.42E-05
A0A8P0P 5Y9	NFKB1	Nuclear factor NF-kappa-B p105 subunit	-1.48289	0.35777 1408	Down	4.973 E-06
A0A8C0S 3W4	MGARP	Mitochondria localized glutamic acid rich protein	- 1.4824266 67	0.35788 6327	Down	2.038 E-05
A0A8I3M V88	INIP	SOSS complex subunit C	- 1.4799733 33	0.35849 5438	Down	4.509 E-07
A0A8I3P DP4	RAB3A	Ras-related protein Rab-3	- 1.4798966 67	0.35851 449	Down	0.000 1443
A0A8P0N BD5	LDHA	L-lactate dehydrogenase	- 1.4793666 67	0.35864 6221	Down	0.000 1055
A0A8I3P 7W1	RWDD4	RWD domain containing 4	-1.47917	0.35869 5114	Down	1.107 E-06
A0A8I3P 4H3	SDC1	Syndecan	-1.47863	0.35882 9399	Down	8.271 E-05
A0A8C0 QE40	-	Signal peptidase complex catalytic subunit SEC11	- 1.4785066 67	0.35886 0076	Down	1.237 E-06
A0A8I3N 3T0	S100P	S100 calcium binding protein P	- 1.4752966 67	0.35965 9429	Down	5.868 E-05
A0A8C0S EX8	-	Gelsolin	- 1.4735466 67	0.36009 5964	Down	2.531 E-05
A0A8C0P V95	GRAMD 1A	GRAM domain containing 1A	- 1.4732533	0.36016 9187	Down	2.124 E-05

			33			
A0A8C0ML45	-	RNA binding fox-1 homolog 2	-1.471013333	0.360728838	Down	4.566E-06
A0A8C0Z360	CCDC130	Probable splicing factor YJU2B	-1.470523333	0.360851377	Down	2.475E-05
A0A8C0MCL8	LUC7L2	RNA-binding protein Luc7-like 2	-1.470006667	0.360980631	Down	1.681E-05
A0A8I3Q235	GINS1	DNA replication complex GINS protein PSF1	-1.46939	0.361134962	Down	1.271E-05
A0A8C0P1C3	SLC39A1	Solute carrier family 39 member 1	-1.46792	0.361503119	Down	0.0008953
A0A8C0P344	UQCR10	Complex III subunit 9	-1.466393333	0.361885866	Down	2.946E-06
A0A8C0NFA0	RPS8	40S ribosomal protein S8	-1.46541	0.362132609	Down	7.517E-06
A0A8C0PFY4	SVBP	Small vasohibin-binding protein	-1.459703333	0.363567883	Down	9.168E-06
A0A8I3MCJ7	TMEM238	Transmembrane protein 238	-1.457046667	0.364237996	Down	4.379E-06
A0A8C0Z4G4	-	CDV3 homolog	-1.454456667	0.364892482	Down	0.0001196
A0A8C0MB33	-	Myristoylated alanine rich protein kinase C substrate	-1.452823333	0.365305826	Down	1.317E-07
A0A8C0MQ70	-	Charged multivesicular body protein 3	-1.45224	0.365453562	Down	5.695E-06
A0A8I3P7I0	CDH13	Cadherin-13	-1.451596667	0.365616563	Down	1.195E-05
A0A8I3PCV3	ETF1	Eukaryotic peptide chain release factor subunit 1	-1.448236667	0.366469067	Down	3.104E-05
A0A8C0P213	PPP3CA	Serine/threonine-protein phosphatase	-1.448176667	0.366484308	Down	9.746E-06
A0A8C0Q7C7	-	Immediate early response 3-interacting protein 1	-1.448146667	0.366491929	Down	1.636E-06
A0A8I3MMH0	WARS1	Tryptophan--tRNA ligase, cytoplasmic	-1.446383333	0.366940148	Down	0.0001519
A0A8C0TPJ0	-	Myeloid leukemia factor 2	-1.445383333	0.367194579	Down	4.219E-06
A0A8C0N440	-	PNP_UDP_1 domain-containing protein	-1.445186667	0.367244638	Down	5.184E-05
A0A8C0SY36	-	EH domain binding protein 1 like 1	-1.441903333	0.368081378	Down	1.682E-07

A0A8C0SLD4	ESD	S-formylglutathione hydrolase	-1.43915	0.368784519	Down	2.607E-05
P83362	SPCS1	Signal peptidase complex subunit 1	-1.438283333	0.369006125	Down	1.63E-06
Q5WR10	DDX39B	Spliceosome RNA helicase DDX39B	-1.437563333	0.369190329	Down	6.253E-05
A0A8C0NR56	-	Lectin, mannose binding 2 like	-1.434406667	0.369999015	Down	4.929E-06
A0A8I3NML2	CLTB	Clathrin light chain	-1.434133333	0.370069121	Down	9.336E-06
A0A8I3PI76	NDUFC1	NADH dehydrogenase [ubiquinone] 1 subunit C1, mitochondrial	-1.43346	0.37024188	Down	3.694E-07
A0A8I3NKQ4	NDUFA11	NADH dehydrogenase [ubiquinone] 1 alpha subcomplex subunit 11	-1.42965	0.37122094	Down	1.681E-05
A0A8I3NEJ5	PPIL1	Peptidyl-prolyl cis-trans isomerase	-1.4284	0.371542718	Down	6.144E-09
A0A8C0MK82	-	Ras-related GTP-binding protein	-1.4284	0.371542718	Down	2.988E-05
A0A8C0NLY1	-	KIF-binding protein	-1.428036667	0.371636301	Down	1.296E-05
A0A8P0NLR3	SNTB2	Syntrophin beta 2	-1.42686	0.371939532	Down	7.262E-06
A0A8P0NKR4	MRPL28	39S ribosomal protein L28, mitochondrial	-1.42606	0.372145836	Down	2.823E-06
J9NU25	HSPA13	Heat shock 70 kDa protein 13	-1.424006667	0.372675875	Down	4.302E-06
A0A8C0SZA4	-	Tumor necrosis factor receptor superfamily member 6	-1.423666667	0.372763713	Down	1.905E-05
A0A8C0SIR4	-	TLE family member 3, transcriptional corepressor	-1.421653333	0.373284282	Down	6.422E-06
A0A8I3Q010	UMPS	Uridine 5'-monophosphate synthase	-1.420986667	0.373456816	Down	3.67E-05
A0A8C0Q9T2	SAE1	SUMO1 activating enzyme subunit 1	-1.420843333	0.373493921	Down	2.001E-05
A0A8C0M2A5	PNPO	pyridoxal 5'-phosphate synthase	-1.419413333	0.373864312	Down	1.138E-05
H8ZYY3	TAP1	Transporter associated with antigen presentation 1 (Fragment)	-1.419163333	0.373929103	Down	3.805E-06
A0A8C0YXN0	-	Pseudouridine 5'-phosphatase	-1.4189	0.373997362	Down	2.561E-05
A0A8I3MT73	NAPG	NSF attachment protein gamma	-1.418496667	0.374101935	Down	1.073E-05

A0A8C0REK5	-	Vinculin	-1.417136667	0.37445476	Down	4.11E-05
A0A8C0MGX1	BLVRA	Biliverdin reductase A	-1.414613333	0.37511027	Down	4.708E-05
A0A8C0M689	TGFB1	Transforming growth factor beta	-1.412043333	0.375779082	Down	1.095E-06
A0A8I3PM31	MANSC1	MANSC domain containing 1	-1.411496667	0.3759215	Down	4.72E-05
A0A8C0PBF7	-	SH3 domain-containing protein	-1.40947	0.376449957	Down	0.00046
A0A8P0TNE8	CD99	CD99 molecule (Xg blood group)	-1.40834	0.376744929	Down	2.417E-05
A0A8C0Q0Y5	-	Pyr_redox_2 domain-containing protein	-1.408053333	0.376819797	Down	1.278E-05
A0A8C0TD80	-	Phospholipid-transporting ATPase	-1.407653333	0.376924288	Down	7.527E-05
A0A8C0N022	-	Annexin	-1.406566667	0.377208302	Down	4.921E-05
A0A8I3N6K0	TLN1	Talin 1	-1.405346667	0.377527419	Down	8.678E-06
A0A8C0YVW8	-	Histone acetyltransferase	-1.405206667	0.377564056	Down	3.95E-06
A0A8C0PGH0	-	Profilin	-1.40488	0.377649557	Down	1.451E-05
A0A8C0TJ18	PIN1	Peptidyl-prolyl cis-trans isomerase	-1.40364	0.377974287	Down	6.004E-05
A0A8I3NWQ3	PRDX6	Peroxiredoxin-6	-1.402083333	0.378382341	Down	0.0001183
A0A8C0NZR2	NDUFA4	NDUFA4 mitochondrial complex associated	-1.400886667	0.378696327	Down	2.477E-05
A0A8C0Q3H0	TAF10	Transcription initiation factor TFIID subunit 10	-1.399806667	0.378979925	Down	2.539E-06
A0A8I3PI25	RBM12	Copine 1	-1.399656667	0.37901933	Down	5.016E-06
A0A8P0PPA4	CARHSP1	CSD domain-containing protein	-1.39753	0.379578452	Down	4.718E-05
A0A8C0N759	-	Adenosine deaminase	-1.394996667	0.380245567	Down	5.304E-06
A0A8C0RBL2	-	Transcriptional adapter	-1.393856667	0.380546151	Down	0.0008421
A0A8C0RYJ6	PLA2G4A	Phospholipase A2	-1.3886833	0.381913194	Down	1.629E-05

			33			
A0A8I3PLN8	CHMP4B	Charged multivesicular body protein 4B	-1.38828	0.38201998	Down	1.055E-05
A0A8C0RHT7	-	Thioredoxin domain-containing protein 12	-1.387446667	0.382240707	Down	1.855E-07
A0A8C0NAC0	-	Telomeric repeat binding factor 2	-1.384413333	0.383045232	Down	3.756E-06
A0A8C0MNX1	MYH9	Myosin-9	-1.38125	0.383886039	Down	1.552E-05
A0A8C0TRK4	ALKBH7	AlkB homolog 7	-1.379036667	0.384475436	Down	4.463E-05
A0A8I3P3P4	ACSS2	propionate--CoA ligase	-1.378323333	0.384665585	Down	2.002E-05
A0A8C0MM41	AASDHPPT	L-aminoadipate-semialdehyde dehydrogenase-phosphopantetheinyl transferase	-1.37754	0.384874502	Down	0.0001463
A0A8C0SPS0	-	GST C-terminal domain-containing protein	-1.376523333	0.385145818	Down	6.574E-06
A0A8C0TZG8	-	Methyltransferase like 21A	-1.37546	0.385429793	Down	2.986E-05
A0A8C0MNU1	GABPB2	GA binding protein transcription factor subunit beta 2	-1.374746667	0.385620414	Down	0.0005555
A0A8I3PNF1	LDHD	Lactate dehydrogenase D	-1.374296667	0.385740714	Down	0.0008206
A0A8I3NDL3	CAPN2	Calpain-2 catalytic subunit	-1.37311	0.38605813	Down	2.681E-05
A0A8I3P5A5	GBE1	1,4-alpha-glucan branching enzyme	-1.371293333	0.386544567	Down	3.326E-05
A0A8I3N423	P4HA2	procollagen-proline 4-dioxygenase	-1.370586667	0.386733952	Down	1.468E-05
A0A8I3N734	NUDC	Nuclear migration protein nudC	-1.36907	0.387140729	Down	7.127E-05
A0A8C0PDY6	CD82	CD82 molecule	-1.369013333	0.387155936	Down	6.977E-08
A0A8C0TU87	RFC5	Replication factor C subunit 5	-1.368923333	0.387180089	Down	8.318E-07
A0A8C0QFX1	BHLHE40	Basic helix-loop-helix family member e40	-1.366083333	0.387943018	Down	1.1E-05
A0A8C0T144	FNTA	Farnesyltransferase, CAAX box, alpha	-1.364256667	0.388434523	Down	7.168E-06
A0A8C0PYV2	-	RNA 3'-terminal-phosphate cyclase (ATP)	-1.3616433	0.38913878	Down	6.56E-05

			33			
A0A8C0YZN5	LOC609365	Membrane cofactor protein	-1.35998	0.389587691	Down	8.887E-06
A0A8I3NY06	PTBP1	Polypyrimidine tract-binding protein 1	-1.357573333	0.390238133	Down	9.266E-07
A0A8C0LWB7	-	Mitochondrial ribosomal protein L27	-1.35681	0.390444664	Down	1.778E-05
A0A8C0TAE3	-	Cytochrome b5 heme-binding domain-containing protein	-1.351186667	0.391969508	Down	8.059E-06
A0A8C0RTF2	EIF2S3	protein-synthesizing GTPase	-1.349593333	0.392402644	Down	4.431E-06
A0A8C0SK18	-	Chromosome 5 open reading frame 22	-1.348443333	0.39271556	Down	1.53E-05
A0A8C0N4Z0	NDUFB4	NADH dehydrogenase [ubiquinone] 1 beta subcomplex subunit 4	-1.34744	0.392988772	Down	2.842E-05
A0A8C0NRK7	-	BRISC and BRCA1 A complex member 1	-1.344466667	0.393799541	Down	0.0001063
A0A8C0S1A3	HAT1	Histone acetyltransferase type B catalytic subunit	-1.34396	0.393937865	Down	1.163E-05
A0A8C0NBG6	-	ATP binding cassette subfamily B member 10	-1.34255	0.394323064	Down	3.996E-06
E2QXF3	RPL15	60S ribosomal protein L15	-1.342426667	0.394356775	Down	7.628E-06
A0A8I3NKD5	ABT1	Activator of basal transcription 1	-1.339233333	0.395230631	Down	2.113E-07
A0A8C0T8N5	B3GAT3	Galactosylgalactosylxylosyl protein 3-beta-glucuronosyltransferase	-1.335006667	0.396390237	Down	1.957E-07
A0A8I3RV50	EEF2	Eukaryotic translation elongation factor 2	-1.33465	0.396488245	Down	5.923E-05
A0A8C0SPG6	-	Ubiquitin carboxyl-terminal hydrolase	-1.33151	0.397352135	Down	5.012E-07
A0A8I3PKI7	PNP	Purine nucleoside phosphorylase	-1.329593333	0.397880381	Down	7.002E-05
A0A8I3RVD9	NAB2	NGFI-A binding protein 2	-1.32888	0.398077159	Down	2.014E-05
A0A8I3NXH4	NSFL1C	NSFL1 cofactor p47	-1.325443333	0.399026555	Down	6.277E-05
A0A8I3Q0V4	BCCIP	BRCA2 and CDKN1A-interacting protein	-1.325426667	0.399031165	Down	9.777E-06
A0A8C0SWD7	SS18L2	Natural killer cell triggering receptor	-1.323386667	0.399595802	Down	4.005E-06
A0A8I3MJ17	ABI1	Abl interactor 1	-1.322446667	0.399856247	Down	7.853E-07

A0A8I3NMC9	WIPI2	WD repeat domain phosphoinositide-interacting protein 2	-1.320573333	0.400375796	Down	0.0001938
P62822	RAB1A	Ras-related protein Rab-1A	-1.31972	0.400612683	Down	4.951E-06
A0A8C0Z0W2	RNF5	RING-type E3 ubiquitin transferase	-1.319193333	0.400758956	Down	0.0005924
A0A8I3PA53	POFUT2	GDP-fucose protein O-fucosyltransferase 2	-1.318716667	0.400891389	Down	3.221E-06
A0A8C0T3G3	-	Intraflagellar transport protein 57 homolog	-1.318433333	0.400970128	Down	7.814E-06
A0A8I3P6A1	FARS2	phenylalanine--tRNA ligase	-1.31833	0.400998849	Down	5.076E-06
A0A8I3P0L7	NAGPA	N-acetylglucosamine-1-phosphodiester alpha-N-acetylglucosaminidase	-1.31796	0.401101704	Down	0.00022
A0A8I3NME9	B4GAT1	Beta-1,4-glucuronyltransferase 1	-1.315306667	0.401840069	Down	0.000358
A0A8C0SDM7	VAMP3	Vesicle associated membrane protein 3	-1.314996667	0.401926424	Down	3.884E-06
A0A8C0TIJ2	MAPK1	Mitogen-activated protein kinase	-1.31482	0.401975645	Down	9.142E-05
A0A8C0TR41	EIF2AK2	Interferon-induced, double-stranded RNA-activated protein kinase	-1.31426	0.402131707	Down	2.155E-06
A0A8P0SDD0	POLE4	CBFD_NFYB_HMF domain-containing protein	-1.31322	0.402421698	Down	3.306E-06
A0A8C0Q7G1	-	Phosphoserine aminotransferase	-1.31316	0.402438434	Down	0.0001226
A0A8I3P1M9	WRNIP1	WRN helicase interacting protein 1	-1.313103333	0.402454242	Down	1.335E-05
A0A8P0NCV6	NCOA3	Nuclear receptor coactivator	-1.311626667	0.402866384	Down	1.014E-05
P61902	COX6A1	Cytochrome c oxidase subunit 6A1, mitochondrial (Fragment)	-1.30634	0.404345371	Down	0.0004689
A0A8C0RFH9	-	Hypoxia-inducible gene 2 protein	-1.30271	0.405364035	Down	0.000246
A0A8C0RP65	-	TAR DNA-binding protein 43	-1.3025	0.405423045	Down	3.735E-06
A0A8C0S4C7	ACLY	ATP-citrate synthase	-1.30225	0.405493305	Down	1.797E-05
A0A8C0SS15	EXT1	Exostosin-1	-1.301406667	0.405730407	Down	3.174E-05
A0A8C0SMA2	AARS1	Alanine--tRNA ligase	-1.299013333	0.406404045	Down	3.716E-05
A0A8I3PRW9	PRKACA	cAMP-dependent protein kinase	-1.29865	0.406506408	Down	3.853E-08

A0A8I3RWY9	VDAC1	Voltage-dependent anion-selective channel protein 1	-1.29837	0.406585311	Down	6.359E-05
A0A8I3N3L8	CPT1A	carnitine O-palmitoyltransferase	-1.29751	0.406827751	Down	8.864E-06
A0A8P0PMC4	SRRM1	PWI domain-containing protein	-1.296163333	0.407207677	Down	3.345E-06
A0A8C0M725	-	NUMB like endocytic adaptor protein	-1.295793333	0.407312125	Down	6.145E-06
A0A8I3MUD3	SCO1	Synthesis of cytochrome C oxidase 1	-1.29563	0.407358241	Down	1.154E-06
A0A8P0TLX4	ALG1	Glycos_transf_1 domain-containing protein	-1.294993333	0.407538049	Down	7.053E-06
A0A8C0M746	-	Serine/arginine-rich splicing factor 2	-1.293696667	0.407904501	Down	1.265E-05
A0A8I3PRB6	-	Cytochrome b5 heme-binding domain-containing protein	-1.292036667	0.408374116	Down	1.098E-06
A0A8I3RXB5	MPDZ	Multiple PDZ domain crumbs cell polarity complex component	-1.290493333	0.408811211	Down	5.851E-07
A0A8I3NFC4	GLRX	Glutaredoxin	-1.28959	0.409067266	Down	5.124E-05
A0A8I3N364	E2F4	E2F transcription factor 4	-1.28934	0.409138158	Down	2.975E-05
A0A8C0P9F1	-	Palladin, cytoskeletal associated protein	-1.286773333	0.409866694	Down	1.474E-05
A0A8C0QM52	MRPL53	Mitochondrial ribosomal protein L53	-1.28634	0.409989822	Down	3.88E-05
A0A8C0MPU7	CETN3	Centrin 3	-1.28539	0.410259885	Down	4.516E-06
A0A8I3PWF6	C27H12orf57	Protein C10	-1.284323333	0.410563326	Down	1.171E-05
A0A8C0Q5V9	-	Trans-Golgi network integral membrane protein 2	-1.284123333	0.410620246	Down	1.041E-05
A0A8I3Q2V8	-	Ribosomal_L23eN domain-containing protein	-1.28315	0.41089737	Down	1.708E-05
A0A8I3Q774	NDUFA7	NADH dehydrogenase [ubiquinone] 1 alpha subcomplex subunit 7	-1.281986667	0.411228835	Down	9.232E-05
A0A8I3N081	GNG10	Guanine nucleotide-binding protein subunit gamma	-1.2801	0.411766966	Down	3.347E-05
A0A8P0SF01	CTSA	Carboxypeptidase	-1.279286667	0.411999169	Down	5.107E-06
A0A8I3PG65	CD44	CD44 antigen	-1.279166667	0.41203344	Down	1.473E-05
A0A8I3RT39	PSMB6	Proteasome subunit beta	-1.27883	0.412129603	Down	2.58E-05
A0A8C0P	CEMIP2	hyaluronoglucosaminidase	-1.27793	0.41238	Down	1.377

AJ7				6783		E-07
A0A8I3NFS3	NUBP1	Cytosolic Fe-S cluster assembly factor NUBP1	-1.276273333	0.412860604	Down	4.136E-05
A0A8I3NGF8	MAPRE2	Microtubule-associated protein RP/EB family member 2	-1.27459	0.41334261	Down	0.0004911
A0A8C0M6X1	CIAPIN1	Anamorsin	-1.272866667	0.413836653	Down	0.0001324
A0A8I3NB10	DNAJC8	DnaJ heat shock protein family (Hsp40) member C8	-1.27254	0.413930368	Down	0.0001028
A0A8C0QAG4	ABCF2	ATP binding cassette subfamily F member 2	-1.267983333	0.415239809	Down	1.081E-05
A0A8C0MDV5	HSD17B12	Hydroxysteroid 17-beta dehydrogenase 12	-1.266653333	0.415622789	Down	8.494E-06
A0A8C0YZY6	PRMT1	Protein arginine methyltransferase 1	-1.266276667	0.415731316	Down	1.475E-05
A0A8I3NPQ9	WFS1	Wolframin ER transmembrane glycoprotein	-1.265683333	0.415902328	Down	1.906E-06
A0A8C0NG77	STMN1	Stathmin	-1.263383333	0.416565905	Down	0.0001209
A0A8P0T578	-	Mago homolog B, exon junction complex subunit	-1.262446667	0.416836447	Down	4.75E-05
A0A8I3N636	NDUFV1	NADH dehydrogenase [ubiquinone] flavoprotein 1, mitochondrial	-1.26207	0.416945291	Down	7.306E-06
A0A8C0MIT4	-	78 kDa glucose-regulated protein	-1.26175	0.417037783	Down	1.433E-05
P61162	ACTR1A	Alpha-centractin	-1.260526667	0.41739156	Down	3.546E-05
A0A8C0TCN1	WDR82	WD repeat domain 82	-1.260133333	0.417505372	Down	9.311E-06
A0A8I3MMA0	CPNE2	Copine 2	-1.259276667	0.417753359	Down	0.0001929
A0A8C0RYD6	IKBKB	IkappaB kinase	-1.258533333	0.417968657	Down	2.478E-06
A0A8I3MJF3	NARS1	asparagine--tRNA ligase	-1.257626667	0.418231414	Down	0.00011
A0A8P0SJI6	CD9	Tetraspanin	-1.257226667	0.418347388	Down	1.535E-05
A0A8I3N9H6	LOX	Lysyl oxidase homolog	-1.256966667	0.418422789	Down	2.708E-05
A0A8C0T	TIAL1	TIA1 cytotoxic granule	-	0.41893	Down	4.882

WW4		associated RNA binding protein like 1	1.2551866 67	9358		E-08
A0A8I3N S03	KIAA114 3	KIAA1143	- 1.2548933 33	0.41902 4547	Down	0.0001 163
A0A8P0N PG5	IGSF8	Immunoglobulin superfamily member 8	- 1.2546866 67	0.41908 4577	Down	3.224 E-05
A0A8C0P CV2	RPL34	60S ribosomal protein L34	- 1.2546466 67	0.41909 6196	Down	1.75E-05
A0A8C0 NM32	MRPS12	Mitochondrial ribosomal protein S12	- 1.2545033 33	0.41913 7836	Down	1.124 E-05
A0A8I3N PV3	UPK1B	Tetraspanin	-1.25399	0.41928 6998	Down	4.324 E-05
A0A8I3Q 9N1	ZFX	Zinc finger X-chromosomal protein	-1.25394	0.41930 153	Down	0.000 1095
A0A8C0S BB7	HNRNPA 0	Heterogeneous nuclear ribonucleoprotein A0	-1.25328	0.41949 3395	Down	4.699 E-06
A0A8I3P RJ5	CGGBP1	CGG triplet repeat binding protein 1	- 1.2532333 33	0.41950 6964	Down	2.697 E-05
A0A8C0 QR36	CENPS	Peroxisomal membrane protein PEX14	- 1.2526033 33	0.41969 0196	Down	2.236 E-06
A0A8C0S BA6	CTPS1	CTP synthase	- 1.2525233 33	0.41971 3469	Down	1.846 E-05
A0A8I3R XF5	YIPF4	Protein YIPF	- 1.2510733 33	0.42013 552	Down	9.437 E-06
A0A8I3N ED2	PALM2A KAP2	AKAP2_C domain-containing protein	-1.24924	0.42066 9755	Down	1.018 E-05
A0A8C0T US2	-	CAP10 domain-containing protein	- 1.2485566 67	0.42086 9052	Down	7.399 E-06
A0A8C0S BC0	-	Aryl hydrocarbon receptor nuclear translocator	- 1.2484066 67	0.42091 2813	Down	9.41E-07
A0A8C0 REM2	-	Urokinase-type plasminogen activator	- 1.2473633 33	0.42121 7321	Down	2.093 E-05
A0A8C0P X83	PIP4K2B	Phosphatidylinositol-5-phosphate 4-kinase type 2 beta	- 1.2398133 33	0.42342 7439	Down	3.99E-05
A0A8C0P FL2	MCMBP	Mini-chromosome maintenance complex-binding protein	-1.23931	0.42357 5192	Down	2.281 E-05
A0A8C0 M6E9	-	THUMP domain-containing protein	-1.23849	0.42381 6012	Down	4.456 E-05
A0A8C0T 7F9	-	60S ribosomal protein L14	- 1.2377033 33	0.42404 7172	Down	4.421 E-06
A0A8C0S GE8	-	Complex1_LYR_dom domain-containing protein	-1.23689	0.42428 63	Down	3.37E-06
A0A8C0	-	60S ribosomal protein L32	-	0.42442	Down	3.236

RQX7			1.236403333	9449		E-05
A0A8C0Z7L6	DYNLL2	Dynein light chain	-1.236206667	0.424487311	Down	1.816E-06
A0A8C0QJ80	-	vitamin-K-epoxide reductase (warfarin-sensitive)	-1.23602	0.424542238	Down	1.785E-05
A0A8C0S297	IPO7	Importin 7	-1.23523	0.424774775	Down	5.974E-06
A0A8C0LWH8	-	Chloride intracellular channel protein	-1.234623333	0.424953434	Down	1.349E-05
A0A8C0SZN9	LSM12	LSM12 homolog	-1.23413	0.425098773	Down	2.579E-06
A0A8P0N8I0	ARHGDIA	Rho GDP dissociation inhibitor alpha	-1.2338	0.425196021	Down	7.465E-05
A0A8C0RUL0	GNPNAT1	Glucosamine 6-phosphate N-acetyltransferase	-1.232023333	0.425719968	Down	8.123E-05
A0A8C0P5R7	-	Collagen type V alpha 1 chain	-1.23196	0.425738658	Down	8.993E-05
A0A8C0Z0Q7	UCK2	Uridine-cytidine kinase	-1.231906667	0.425754397	Down	8.344E-05
A0A8I3NNT6	BLMH	Bleomycin hydrolase	-1.231	0.426022048	Down	2.277E-05
A0A8I3N3L9	SEC61B	Protein transport protein Sec61 subunit beta	-1.230913333	0.426047641	Down	1.528E-05
A0A8C0S3P8	WWOX	WW domain-containing oxidoreductase	-1.230906667	0.426049609	Down	1.329E-05
A0A8C0TIF6	MCCC1	Methylcrotonoyl-CoA carboxylase 1	-1.23051	0.426166767	Down	2.378E-06
A0A8C0TI19	UFD1	Ubiquitin recognition factor in ER associated degradation 1	-1.230273333	0.426236683	Down	5.071E-05
A0A8I3NNQ3	MAP1LC3A	Dynein light chain roadblock-type 1	-1.228353333	0.426804315	Down	6.925E-05
A0A8I3S2Y1	RBM12	Copine 1	-1.22662	0.427317409	Down	7.734E-06
A0A8P0NKA3	SGTA	Small glutamine rich tetratricopeptide repeat co-chaperone alpha	-1.225996667	0.427502077	Down	2.15E-05
A0A8C0MZI9	-	Tumor necrosis factor receptor type 1-associated DEATH domain protein	-1.224823333	0.427849902	Down	5.377E-05
A0A8I3PF76	PSMD10	Proteasome 26S subunit, non-ATPase 10	-1.224573333	0.42792405	Down	4.736E-05
A0A8C0PCX6	DPM3	Dolichol-phosphate mannosyltransferase subunit 3	-1.224226667	0.428026888	Down	6.98E-05
A0A8I3MKN8	TOR1AIP1	Torsin 1A interacting protein 1	-1.22387	0.428132719	Down	5.809E-06

A0A8I3NA73	WASF2	Wiskott-Aldrich syndrome protein family member	-1.222166667	0.428638497	Down	7.748E-07
A0A8C0T7F4	NOB1	RNA-binding protein NOB1	-1.221346667	0.428882196	Down	1.815E-05
A0A8I3PEA3	CCT5	T-complex protein 1 subunit epsilon	-1.220656667	0.429087368	Down	3.522E-05
A0A8C0MVQ9	NAPA	NSF attachment protein alpha	-1.220396667	0.429164704	Down	2.59E-06
A0A8P0NLY2	ALDOA	Fructose-bisphosphate aldolase	-1.21744	0.430045138	Down	0.0001399
A0A8C0NWL5	RPS20	40S ribosomal protein S20	-1.21647	0.430334377	Down	2.439E-05
A0A8C0MEI8	-	YKT6 v-SNARE homolog	-1.216006667	0.430472605	Down	1.334E-05
A0A8C0SIN3	-	Nonsense-mediated mRNA decay factor SMG9	-1.215596667	0.430594959	Down	3.199E-05
A0A8I3NJF2	REL	REL proto-oncogene, NF-kB subunit	-1.21403	0.431062809	Down	2.152E-05
A0A8I3NQ19	MYO1E	Myosin IE	-1.213656667	0.431174371	Down	1.616E-05
A0A8C0RLL1	-	Filamin A	-1.210593333	0.432090874	Down	0.0002854
A0A8I3RRR7	LOC100687259	AAA domain-containing protein	-1.21018	0.432214686	Down	4.758E-06
A0A8C0MPI6	UBXN1	UBX domain protein 1	-1.20935	0.432463416	Down	2.514E-05
A0A8C0RXI8	-	Myosin phosphatase Rho interacting protein	-1.207936667	0.432887286	Down	3.242E-06
A0A8I3S614	PDLIM1	PDZ and LIM domain 1	-1.207863333	0.43290929	Down	3.772E-05
A0A8C0P0R4	KPNA6	Importin subunit alpha	-1.207273333	0.433086368	Down	1.582E-06
A0A8I3PE87	-	60S ribosomal protein L30	-1.20545	0.433634065	Down	0.0001137
A0A8C0SD11	VARS1	Valine--tRNA ligase	-1.204206667	0.434007938	Down	1.357E-06
A0A8C0T1I1	-	RAB35, member RAS oncogene family	-1.20184	0.434720489	Down	0.0001029
A0A8I3NPK0	RSU1	Ras suppressor protein 1	-1.200793333	0.435035991	Down	4.602E-05
A0A8I3PMI1	ARF4	ADP-ribosylation factor	-1.200606667	0.435092283	Down	2.329E-05
A0A8I3Q	GSTO1	Glutathione S-transferase	-	0.43590	Down	9.738

SL3		omega	1.1979033 33	8326		E-05
A0A8C0 M7Z8	CHCHD5	Coiled-coil-helix-coiled-coil-helix domain containing 5	-1.19657	0.43631 1378	Down	0.000 1034
A0A8I3P FN0	MYO1B	Myosin IB	- 1.1956033 33	0.43660 3823	Down	0.000 234
A0A8I3M ME5	TTC13	Tetratricopeptide repeat domain 13	- 1.1927633 33	0.43746 414	Down	1.652 E-05
B2G3R6	b4GalT4	Beta-1,4-galactosyltransferase	-1.19245	0.43755 9162	Down	4.289 E-06
A0A8C0 YXX5	CAP1	Adenylyl cyclase-associated protein	-1.191	0.43799 9158	Down	0.000 1025
A0A8I3P LJ3	COPB1	Coatomer subunit beta	- 1.1897566 67	0.43837 6794	Down	4.207 E-06
A0A8C0 MDD5	RPL22	Ribosomal protein L22	- 1.1897466 67	0.43837 9832	Down	9.129 E-06
A0A8C0S XV3	SPR	Sepiapterin reductase	- 1.1895866 67	0.43842 8453	Down	2.628 E-05
A0A8C0 RKC2	KPNA4	Importin subunit alpha	-1.18937	0.43849 4302	Down	2.102 E-06
A0A8I3N IJ2	PAFAH1 B1	Platelet-activating factor acetylhydrolase IB subunit alpha	- 1.1891433 33	0.43856 32	Down	9.024 E-06
A0A8C0T FQ6	NDUFB3	NADH dehydrogenase [ubiquinone] 1 beta subcomplex subunit 3	-1.18754	0.43905 0867	Down	6.987 E-06
A0A8I3P HS5	PFN2	Profilin	- 1.1872133 33	0.43915 0292	Down	4.351 E-05
A0A8P0N VV9	TTC38	Tetratricopeptide repeat protein 38	- 1.1870533 33	0.43919 8998	Down	2.186 E-05
A0A8C0 MW41	CORO6	Coronin	- 1.1862333 33	0.43944 8701	Down	5.66E-05
A0A8C0 M9N5	-	Trafficking protein particle complex subunit	- 1.1861433 33	0.43947 6116	Down	1.68E-05
A0A8C0S ED0	TBP	TATA-box binding protein	- 1.1851133 33	0.43978 9988	Down	7.075 E-06
A0A8C0S XC4	ELOC	Elongin-C	- 1.1842833 33	0.44004 3077	Down	3.776 E-06
A0A8I3N Y33	AHCY	Adenosylhomocysteinase	- 1.1835966 67	0.44025 2571	Down	7.345 E-05
A0A8C0R T67	-	Cortactin	- 1.1811033 33	0.44101 4094	Down	1.165 E-06
A0A8I3P	SFXN3	Sidoreflexin	-	0.44105	Down	6.403

BS0			1.180976667	2816		E-06
A0A8C0NCN5	DYNLT1	Dynein light chain Tctex-type 1	-1.18078	0.441112944	Down	0.0001254
A0A8P0TGJ6	FAT1	FAT atypical cadherin 1	-1.179936667	0.441370874	Down	4.829E-06
A0A8C0PSJ2	-	Thioredoxin domain-containing protein 17	-1.17903	0.441648342	Down	0.0002142
A0A8P0PIJ3	GNAI2	Guanine nucleotide-binding protein G(i) subunit alpha-2	-1.178863333	0.441699366	Down	8.454E-06
A0A8C0RM88	ELOVL5	Elongation of very long chain fatty acids protein	-1.178786667	0.441722839	Down	1.659E-05
A0A8C0MWY5	TAX1BP3	Tax1-binding protein 3	-1.178586667	0.441784079	Down	0.0001618
A0A8C0MAM9	API5	Apoptosis inhibitor 5	-1.178223333	0.441895354	Down	6.071E-06
A0A8I3RRH5	ITIH2	Inter-alpha-trypsin inhibitor heavy chain 2	-1.178103333	0.441932111	Down	9.984E-05
A0A8I3RZQ6	CDKN2AIPNL	CDKN2A interacting protein N-terminal like	-1.17636	0.442466459	Down	0.0001644
A0A8P0TJA1	N4BP1	NEDD4 binding protein 1	-1.17461	0.443003499	Down	0.0001522
A0A8P0N517	CYP51A1	Cytochrome P450 family 51 subfamily A member 1	-1.17344	0.443362913	Down	3.744E-05
A0A8I3PF51	METAP2	Methionine aminopeptidase 2	-1.173383333	0.443380328	Down	1.763E-05
A0A8I3MMT3	CDR2	Cerebellar deration related protein 2	-1.17281	0.443556564	Down	1.359E-05
A0A8I3MEC8	SUB1	Activated RNA polymerase II transcriptional coactivator p15	-1.170816667	0.444169838	Down	9.137E-05
A0A8C0NC57	VPS35	Vacuolar protein sorting-associated protein 35	-1.17055	0.444251946	Down	6.727E-06
A0A8I3S301	LOC100856782	T-complex protein 1 subunit eta	-1.16995	0.444436743	Down	9.321E-06
A0A8C0PY00	ILF2	Interleukin enhancer binding factor 2	-1.16258	0.446712955	Down	3.269E-07
A0A8I3N041	ACAT2	Acetyl-CoA acetyltransferase 2	-1.162413333	0.446764564	Down	9.255E-06
A0A8I3S7P0	DDX17	RNA helicase	-1.16207	0.446870898	Down	2.559E-07
A0A8I3NMT8	-	Cytochrome c oxidase subunit 7C, mitochondrial	-1.162046667	0.446878126	Down	5.126E-05
A0A8C0PU63	CBWD1	CobW C-terminal domain-containing protein	-1.161833333	0.446944211	Down	7.723E-06
L7V3M2	NDRG1	Protein NDRG1	-1.15871	0.447912862	Down	0.0003209

A0A8P0SH90	PSMD2	26S proteasome non-ATPase regulatory subunit 2	-1.15855	0.44796254	Down	5.085E-06
A0A8C0QID1	COMMD3	RING-type domain-containing protein	-1.157876667	0.448171662	Down	4.367E-05
A0A8C0S0Y4	CFAP20	Cilia and flagella associated protein 20	-1.157203333	0.448380881	Down	1.997E-05
A0A8P0PP94	GPC1	Glypican-1	-1.156626667	0.448560141	Down	2.4E-05
A0A8C0S9Y1	PTPN1	Tyrosine-protein phosphatase non-receptor type	-1.155943333	0.448772652	Down	1.289E-06
A0A8C0YZD9	PKM	Pyruvate kinase	-1.154403333	0.449251949	Down	9.591E-05
A0A8I3N399	COPS6	COP9 signalosome complex subunit 6	-1.153563333	0.449513599	Down	7.196E-06
A0A8I3S6Q5	PSMD13	26S proteasome non-ATPase regulatory subunit 13	-1.152853333	0.449734875	Down	2.963E-05
A0A8C0YWD7	-	Dolichol-phosphate mannosyltransferase subunit 1	-1.152506667	0.449842955	Down	6.311E-06
A0A8C0MXL9	GORASP2	Golgi reassembly stacking protein 2	-1.15049	0.450472206	Down	1.044E-05
A0A8I3Q4Z9	PEA15	Astrocytic phosphoprotein PEA-15	-1.14926	0.450856429	Down	0.0002002
A0A8C0SD66	-	F-actin monooxygenase	-1.14841	0.451122141	Down	3.587E-06
A0A8I3PZN2	BRWD1	Proteasome assembly chaperone 1	-1.148396667	0.45112631	Down	2.39E-05
A0A8C0RJE5	GNAI3	G protein subunit alpha i3	-1.14835	0.451140903	Down	8.928E-06
A0A8C0PDX1	-	Transmembrane and ubiquitin like domain containing 1	-1.14792	0.451275387	Down	2.919E-05
A0A8I3N729	MCRIP1	Mapk-regulated corepressor-interacting protein 1	-1.147136667	0.45152048	Down	1.037E-05
A0A8I3PE10	TOR1B	Torsin	-1.14636	0.451763619	Down	1.681E-06
A0A8C0MKN4	ACAD11	Acyl-CoA dehydrogenase family member 11	-1.146173333	0.451822076	Down	5.498E-06
A0A8C0N6T0	-	Enoyl-CoA delta isomerase 2	-1.144906667	0.452218944	Down	1.234E-05
A0A8C0P4E9	CPSF7	Cleavage and polyadenylation specific factor 7	-1.144226667	0.452432143	Down	2.552E-07
A0A8I3PAU2	IPO5	Importin 5	-1.140893333	0.453478691	Down	2.018E-05

A0A8I3PAL6	PRUNE1	Prune exopolyphosphatase 1	-1.13928	0.45398609	Down	0.0001349
A0A8C0PJL4	DPP9	Dipeptidyl peptidase 9	-1.138753333	0.454151851	Down	3.535E-05
A0A8C0PGW8	-	La-related protein 7	-1.1384	0.454263092	Down	3.55E-06
A0A8I3Q3A8	ICAM1	Intercellular adhesion molecule 1	-1.137403333	0.454577022	Down	4.376E-06
A0A8C0Z002	RELA	RELA proto-oncogene, NF-kB subunit	-1.137333333	0.454599079	Down	4.496E-06
A0A8C0TDT4	RPS13	40S ribosomal protein S13	-1.136753333	0.454781876	Down	2.799E-05
A0A8C0MF73	TMEM263	Transmembrane protein 263	-1.134026667	0.455642218	Down	6.119E-06
A0A8C0S1Q7	-	Importin subunit alpha	-1.132813333	0.456025583	Down	8.544E-08
O02812	MAPK14	Mitogen-activated protein kinase 14	-1.13239	0.456159415	Down	1.158E-05
A0A8C0RBA8	SREK1	Splicing regulatory glutamic acid and lysine rich protein 1	-1.13189	0.456317535	Down	6.901E-06
A0A8C0NCZ1	-	NudC domain containing 2	-1.131613333	0.456405052	Down	0.0002324
A0A8C0MEE7	-	40S ribosomal protein S12	-1.130066667	0.456894612	Down	5.441E-06
A0A8I3Q1P7	YWHAZ	14_3_3 domain-containing protein	-1.12786	0.457593987	Down	0.0001057
A0A8I3NIZ9	STX12	Syntaxin 12	-1.12622	0.458114458	Down	9.831E-06
A0A8C0SCD5	-	Ceramide synthase 2	-1.125826667	0.458239374	Down	1.096E-05
A0A8I3PCR7	RPL24	Ribosomal protein L24	-1.122266667	0.459371524	Down	7.609E-05
A0A8C0S1K7	-	Isocitrate dehydrogenase [NADP]	-1.12189	0.459491475	Down	8.135E-05
A0A8I3PST5	GPX8	Glutathione peroxidase	-1.121753333	0.459535005	Down	2.682E-05
A0A8I3N9V3	HSP90AB1	Heat shock protein 90 alpha family class B member 1	-1.120746667	0.459855765	Down	0.0022046
A0A8C0T901	-	Pseudouridine synthase 1	-1.11748	0.460898188	Down	0.0002134
A0A8P0P6M6	GANAB	Glucosidase II alpha subunit	-1.11539	0.461566364	Down	1.39E-05
A0A8P0TUJ9	FASN	Fatty acid synthase	-1.1153833	0.461568497	Down	6.964E-05

			33			
A0A8I3RZ83	ACTL6A	Actin like 6A	-1.114556667	0.461833053	Down	5.425E-06
A0A8C0SXK5	-	Neuroplastin	-1.114463333	0.461862931	Down	1.55E-05
A0A8C0M6F0	-	Drebrin 1	-1.1132	0.462267551	Down	1.7E-06
A0A8C0LYN6	-	Calcyclin-binding protein	-1.113083333	0.462304935	Down	0.000116
A0A8C0SEN2	SRP9	Signal recognition particle 9 kDa protein	-1.113076667	0.462307071	Down	2.543E-05
A0A8I3RQT1	NMNAT1	Nicotinamide-nucleotide adenylyltransferase	-1.11297	0.462341253	Down	1.475E-05
A0A8I3PTC7	ZFP36L2	mRNA decay activator protein ZFP36	-1.111546667	0.462797615	Down	1.863E-05
A0A8C0RNU1	COX15	Cytochrome c oxidase assembly homolog COX15	-1.110446667	0.463150615	Down	1.005E-07
A0A8C0PAJ8	-	Kinesin family member 21A	-1.110083333	0.463267271	Down	0.0005443
A0A8C0SB92	PLD3	Phospholipase D family member 3	-1.109836667	0.463346485	Down	3.119E-06
A0A8I3PVV6	RHOA	Transforming protein RhoA	-1.10938	0.463493175	Down	5.658E-07
Q6TEQ7	ANXA2	Annexin A2	-1.109176667	0.463558504	Down	3.331E-06
A0A8C0TEQ5	EIF3G	Eukaryotic translation initiation factor 3 subunit G	-1.10862	0.463737404	Down	1.384E-05
A0A8I3RUA8	H1-0	H1.0 linker histone	-1.107486667	0.464101844	Down	6.964E-07
A0A8C0PQD7	-	Torsin 1A interacting protein 2	-1.107213333	0.464189781	Down	4.328E-05
A0A8C0TF14	MAPK8	Stress-activated protein kinase JNK	-1.107143333	0.464212304	Down	8.257E-06
A0A8I3P949	CCT8	T-complex protein 1 subunit theta	-1.107003333	0.464257353	Down	5.372E-05
A0A8I3NX27	FARSA	Phenylalanine--tRNA ligase alpha subunit	-1.105796667	0.46464582	Down	3.835E-06
A0A8I3NJC6	UQCRQ	Cytochrome b-c1 complex subunit 8	-1.105696667	0.464678028	Down	2.073E-05
A0A8I3NSN3	MTUS1	Microtubule associated scaffold protein 1	-1.105403333	0.464772517	Down	0.0006626

A0A8C0YWQ4	DDX3X	RNA helicase	-1.104383333	0.465101232	Down	2.077E-05
A0A8C0MN34	PARK7	protein deglycase	-1.104196667	0.465161414	Down	7.312E-05
A0A8C0Z2C9	-	Macrophage migration inhibitory factor	-1.10403	0.465215155	Down	0.0002086
A0A8C0T941	-	60S acidic ribosomal protein P2	-1.10273	0.465634545	Down	1.553E-05
A0A8P0T0V8	-	Malate dehydrogenase	-1.102656667	0.465658214	Down	6.764E-05
A0A8I3NR49	HSBP1	Heat shock factor binding protein 1	-1.102046667	0.465855145	Down	9.623E-06
A0A8C0TI21	LIN37	Lin-37 DREAM MuvB core complex component	-1.101453333	0.466046776	Down	0.0002243
A0A8C0RCZ1	FNBP4	Formin binding protein 4	-1.101256667	0.466110311	Down	1.137E-05
A0A8C0N192	-	Cingulin like 1	-1.100693333	0.46629235	Down	5.147E-05
A0A8C0MT28	HM13	Histocompatibility minor 13	-1.100053333	0.46649925	Down	6.677E-06
A0A8I3PS55	FKBP11	peptidylprolyl isomerase	-1.099553333	0.466660954	Down	2.886E-06
A0A8C0YTA3	-	EF-hand domain-containing protein	-1.09949	0.466681441	Down	2.786E-07
A0A8I3PP34	HPRT1	Hypoxanthine phosphoribosyltransferase	-1.096966667	0.4674984	Down	0.0002338
A0A8I3QRT1	BBIP1	BBSome interacting protein 1	-1.096163333	0.467758789	Down	1.418E-05
A0A8C0TKT1	PINX1	XK-related protein	-1.094633333	0.468255117	Down	4.136E-05
A0A8C0NTH2	PRDX2	Peroxiredoxin 2	-1.094303333	0.468362238	Down	0.0001526
A0A8I3PKJ2	SLC39A14	Solute carrier family 39 member 14	-1.09356	0.468603619	Down	1.143E-05
A0A8P0NED7	RECQL	ATP-dependent DNA helicase	-1.092266667	0.469023896	Down	2.841E-06
A0A8I3PZK0	UAP1	UDP-N-acetylglucosamine pyrophosphorylase 1	-1.09218	0.469052073	Down	0.0001552
A0A8C0MVG1	SRPRB	Signal recognition particle receptor subunit beta	-1.091976667	0.469118185	Down	3.126E-05
A0A8C0MFP5	TAF8	Transcription initiation factor TFIID subunit 8	-1.09074	0.469520482	Down	5.343E-06

A0A8P0N621	CCT4	T-complex protein 1 subunit delta	-1.090053333	0.469744009	Down	3.501E-05
A0A8C0R9H0	-	Annexin	-1.08904	0.470074068	Down	0.0003225
A0A8I3P477	MOGS	Mannosyl-oligosaccharide glucosidase	-1.088676667	0.470192468	Down	4.523E-06
A0A8C0RPX2	-	ADP ribosylation factor like GTPase 4C	-1.088423333	0.47027504	Down	2.02E-05
A0A8I3N0K1	PRKACB	cAMP-dependent protein kinase	-1.088376667	0.470290252	Down	2.367E-05
A0A8C0MCR3	-	TNF receptor-associated factor	-1.08791	0.470442401	Down	0.0001439
A0A8C0QKN2	SYNCRIP	Synaptotagmin binding cytoplasmic RNA interacting protein	-1.08588	0.471104821	Down	2.684E-06
A0A8I3MUG5	RPS3A	40S ribosomal protein S3a	-1.084386667	0.471592714	Down	6.984E-05
A0A8C0Q9H7	-	Ras-related protein Rab	-1.08218	0.472314588	Down	2.186E-05
A0A8C0Q9N2	TMEM109	Transmembrane protein 109	-1.079163333	0.473303228	Down	2.266E-06
A0A8I3MT06	YWHAG	Tyrosine 3-monooxygenase/tryptophan 5-monooxygenase activation protein gamma	-1.077986667	0.473689413	Down	3.711E-05
A0A8I3RWX4	RPL8	60S ribosomal protein L8	-1.07792	0.473711303	Down	3.716E-05
A0A8I3PCX9	DRG1	Developmentally regulated GTP binding protein 1	-1.0771	0.473980628	Down	1.96E-06
A0A8C0SMC3	-	Polynucleotide kinase 3'-phosphatase	-1.076586667	0.474149307	Down	4.823E-05
A0A8P0NDP7	EGLN1	Egl-9 family hypoxia inducible factor 1	-1.075833333	0.474396959	Down	4.428E-05
A0A8I3N227	NIP7	60S ribosome subunit biogenesis protein NIP7 homolog	-1.07502	0.47466448	Down	1.58E-05
A0A8C0Q7N4	-	Aminopeptidase	-1.073933333	0.475022142	Down	3.788E-05
A0A8C0T4H7	ALCAM	CD166 antigen	-1.073383333	0.475203269	Down	1.482E-05
A0A8C0Q5W7	ACTR2	Actin related protein 2	-1.07198	0.475665732	Down	4.978E-06
A0A8C0NPL7	-	E3 ubiquitin-protein ligase ZFP91	-1.071386667	0.475861398	Down	0.0006958
A0A8C0MQU2	TMEM126A	Transmembrane protein 126A	-1.07074	0.476074744	Down	1.875E-05

A0A8C0SP22	-	Transmembrane protein 65	-1.07067	0.476097844	Down	6.782E-06
A0A8C0Q740	-	Proteasome subunit alpha type	-1.06995	0.476335507	Down	2.675E-05
A0A8I3NFG8	DCK	Deoxycytidine kinase	-1.06682	0.477370063	Down	0.0002946
P99504	ATP5F1B	ATP synthase subunit beta, mitochondrial (Fragment)	-1.062966667	0.478646788	Down	0.0060235
A0A8C0QI80	-	Ribosome binding protein 1	-1.061133333	0.479262069	Down	1.616E-05
A0A8I3NUR2	RNPEP	Arginyl aminopeptidase	-1.060576667	0.479440382	Down	4.277E-05
A0A8C0MB66	SNRPD2	Small nuclear ribonucleoprotein Sm D2	-1.058206667	0.480228634	Down	8.69E-07
A0A8C0QDJ0	HS2ST1	Heparan sulfate 2-O-sulfotransferase 1	-1.05687	0.480673775	Down	7.912E-06
A0A8I3MQ98	AP2S1	AP complex subunit sigma	-1.056736667	0.480718201	Down	1.787E-05
E2QWF5	FKBP4	peptidylprolyl isomerase	-1.055016667	0.481291661	Down	8.206E-05
A0A8C0PC91	ILVBL	2-hydroxyacyl-CoA lyase 2	-1.05454	0.481450707	Down	1.321E-05
A0A8I3PSN7	KPNA1	Importin subunit alpha	-1.05384	0.481684365	Down	2.603E-05
A0A8C0LY80	KARS1	Lysine--tRNA ligase	-1.053453333	0.481813481	Down	3.314E-05
A0A8C0Z2B3	RPS6	40S ribosomal protein S6	-1.053333333	0.481853559	Down	6.08E-05
A0A8C0Q1M8	HSD17B4	Hydroxysteroid 17-beta dehydrogenase 4	-1.053326667	0.481855786	Down	4.845E-05
A0A8C0S5E2	UBA3	NEDD8-activating enzyme E1 catalytic subunit	-1.053273333	0.481873599	Down	4.795E-05
P42929	HSPB1	Heat shock protein beta-1	-1.052956667	0.481979381	Down	0.0001307
A0A8I3N9F5	CTSD	Cathepsin D	-1.052393333	0.482167617	Down	1.16E-05
A0A8P0NGN3	MAP1S	Microtubule associated protein 1S	-1.051573333	0.48244175	Down	2.334E-05
A0A8C0QMN1	EML2	EMAP like 2	-1.050623333	0.482759537	Down	9.282E-05
A0A8C0RDL4	HARS1	Histidine--tRNA ligase, cytoplasmic	-1.04993	0.482991599	Down	0.0001362
A0A8I3N	RPUSD3	RNA pseudouridine	-	0.48304	Down	0.000

783		synthase D3	1.049766667	6283		3241
A0A8I3PU64	TSN	Translin	-1.04856	0.483450471	Down	4.413E-05
A0A8I3MGN1	MVP	Major vault protein	-1.048383333	0.483509676	Down	4.482E-05
A0A8I3QH08	TBCK	TBC1 domain containing kinase	-1.04708	0.483946677	Down	1.334E-06
A0A8C0MLQ1	SBF2	SET binding factor 2	-1.046616667	0.484102125	Down	5.895E-05
A0A8I3NM68	EVI5	Ecotropic viral integration site 5	-1.045996667	0.484310214	Down	0.0001154
A0A8I3RTV7	MAP4K1	Mitogen-activated protein kinase kinase kinase kinase 1	-1.04566	0.484423245	Down	6.282E-06
A0A8I3N078	ITM2B	Integral membrane protein 2	-1.04566	0.484423245	Down	0.0003589
A0A8C0SQQ7	CELF1	CUGBP Elav-like family member 1	-1.045316667	0.484538542	Down	6.424E-09
A0A8C0T0V7	-	Solute carrier family 3 member 2	-1.044153333	0.484929413	Down	2.411E-05
A0A8I3NSR9	ATXN7	Ataxin 7	-1.043923333	0.485006728	Down	0.000567
A0A8I3NPS8	YBX1	CSD domain-containing protein	-1.04261	0.485448447	Down	1.741E-05
A0A8I3N074	RIPK3	Protein kinase domain-containing protein	-1.041823333	0.485713223	Down	4.592E-05
A0A8I3RV66	STAT3	Signal transducer and activator of transcription	-1.04144	0.485842297	Down	3.271E-05
A0A8I3MPC8	GRWD1	Glutamate rich WD repeat containing 1	-1.04106	0.485970283	Down	1.01E-05
A0A8C0TLT6	TRIP10	Thyroid hormone receptor interactor 10	-1.040773333	0.486066856	Down	2.632E-05
A0A8C0PLW8	RPL7A	60S ribosomal protein L7a	-1.039176667	0.486605096	Down	1.665E-05
A0A8I3P9S2	GTF2H3	General transcription factor IIH subunit 3	-1.038256667	0.486915501	Down	0.0013928
A0A8I3NPY6	SNX12	PX domain-containing protein	-1.037613333	0.487132677	Down	7.642E-05
A0A8P0SBA9	NPLOC4	NPL4 homolog, ubiquitin recognition factor	-1.035313333	0.487909902	Down	2.369E-05
A0A8C0MWJ7	MRPL49	39S ribosomal protein L49, mitochondrial	-1.033893333	0.488390373	Down	9.242E-05
A0A8I3P	CIAO2A	Cytosolic iron-sulfur	-	0.48846	Down	2.884

E98		assembly component 2A	1.0336733 33	4854		E-08
A0A8C0PDB6	-	Thioredoxin related transmembrane protein 4	-1.03314	0.48864 5462	Down	0.0001 167
A0A8C0SWL5	NDUFB7	NADH dehydrogenase [ubiquinone] 1 beta subcomplex subunit 7	-1.03253	0.48885 2115	Down	3.871 E-06
A0A8C0M6H8	CSNK2A2	Casein kinase 2 alpha 2	- 1.0318933 33	0.48906 7895	Down	1.27E-05
A0A8C0RR12	RALB	small monomeric GTPase	- 1.0289433 33	0.49006 8956	Down	2.963 E-05
A0A8P0PMH4	HACD2	Very-long-chain (3R)-3-hydroxyacyl-CoA dehydratase	- 1.0269633 33	0.49074 2004	Down	8.217 E-05
A0A8C0P2Q4	RPS27	40S ribosomal protein S27	- 1.0265966 67	0.49086 6744	Down	1.442 E-05
A0A8C0TV67	-	ADP-ribosylarginine hydrolase	-1.02634	0.49095 4081	Down	8.113 E-05
A0A8I3MXV6	TEAD3	Transcriptional enhancer factor TEF-5	- 1.0253033 33	0.49130 6989	Down	9.521 E-07
A0A8I3P3D2	FDFT1	Squalene synthase	- 1.0251833 33	0.49134 7856	Down	8.486 E-06
A0A8C0NY77	-	Glucoside xylosyltransferase 1	-1.02225	0.49234 7897	Down	0.000 1307
A0A8C0SUA9	DGUOK	STAM binding protein	- 1.0217633 33	0.49251 4009	Down	0.000 1474
A0A8C0NWM5	-	G protein-coupled receptor 108	- 1.0185266 67	0.49362 0198	Down	3.408 E-05
A0A8I3PIA7	NFS1	Cysteine desulfurase, mitochondrial	- 1.0184366 67	0.49365 0993	Down	1.827 E-06
A0A8I3PTG9	ARHGAP1	Rho GTPase activating protein 1	- 1.0182033 33	0.49373 0839	Down	5.146 E-05
A0A8C0QG89	CNPY3	Canopy FGF signaling regulator 3	- 1.0172233 33	0.49406 6337	Down	0.000 2673
A0A8C0LYN2	BLVRB	Biliverdin reductase B	- 1.0171733 33	0.49408 346	Down	0.000 1726
A0A8C0RJU9	TSR1	TSR1 ribosome maturation factor	- 1.0169466 67	0.49416 1093	Down	1.394 E-05
A0A8I3MHS0	CLINT1	Clathrin interactor 1	-1.016	0.49448 5458	Down	1.789 E-05
A0A8C0MDT7	TSFM	Elongation factor Ts, mitochondrial	- 1.0158233 33	0.49454 6015	Down	0.000 8655
A0A8C0TUW0	PITPNB	Phosphatidylinositol transfer protein beta	- 1.0153133	0.49472 087	Down	7.982 E-05

			33			
A0A8I3SCI1	CASP10	Caspase 10	-1.014756667	0.494911796	Down	4.408E-05
A0A8C0TN70	-	Very-long-chain (3R)-3-hydroxyacyl-CoA dehydratase	-1.011933333	0.495881279	Down	2.692E-05
A0A8C0TZ32	-	Nucleoside diphosphate kinase	-1.01186	0.495906486	Down	0.0002028
A0A8C0P3X3	-	Cytochrome P450 family 20 subfamily A member 1	-1.011343333	0.496084115	Down	0.0001376
A0A8C0RS42	PPP2R2A	Serine/threonine-protein phosphatase 2A 55 kDa regulatory subunit B	-1.01123	0.496123087	Down	0.0001403
A0A8C0RH82	CSK	Tyrosine-protein kinase	-1.01097	0.496212506	Down	0.000134
A0A8C0LW98	-	Phosphoglycolate phosphatase	-1.010446667	0.496392538	Down	2.516E-05
A0A8I3N3B7	MYL6	Myosin light chain 6B	-1.009303333	0.496786084	Down	5.87E-05
A0A8I3Q374	SCARB2	Scavenger receptor class B member 2	-1.008413333	0.497092646	Down	7.004E-05
A0A8C0T125	PTPN12	Tyrosine-protein phosphatase non-receptor type 12	-1.008313333	0.497127104	Down	5.104E-05
A0A8C0NQQ3	-	Angiotensin II receptor associated protein	-1.007376667	0.497449967	Down	0.0003436
A0A8I3Q1W2	CNOT9	CCR4-NOT transcription complex subunit 9	-1.007073333	0.497554569	Down	7.279E-06
A0A8C0RK12	AP3B1	AP-3 complex subunit beta	-1.0057	0.498028428	Down	1.14E-05
A0A8C0LYF7	-	TNF alpha induced protein 2	-1.00462	0.498401391	Down	4.946E-06
A0A8C0TCN4	GDI1	Rab GDP dissociation inhibitor	-1.00434	0.498498131	Down	6.569E-05
A0A8I3NJ50	FEN1	Flap endonuclease 1	-1.004103333	0.498579914	Down	0.0001093
A0A8C0PR08	SUPT4H1	Transcription elongation factor SPT4	-1.00339	0.498826495	Down	2.253E-06
A0A8C0S4J3	ITGB3	Integrin beta	-1.003216667	0.49888643	Down	1.296E-08
A0A8I3PH25	EMC7	ER membrane protein complex subunit 7	-1.003033333	0.498949831	Down	1.284E-05
A0A8I3RSS1	SLC1A5	Amino acid transporter	-1.002053333	0.499288875	Down	2.394E-05
A0A8C0NKP8	MOB1B	MOB kinase activator 1B	-1.0020466	0.499291182	Down	0.0002062

			67			
A0A8I3Q IQ3	EIF1AX	Eukaryotic translation initiation factor 4C	- 1.0019633 33	0.49932 0023	Down	2.886 E-05
A0A8I3P V70	TOR1A	Torsin	-1.00191	0.49933 8482	Down	3.431 E-05
A0A8C0R W07	YTHDF2	YTH N6-methyladenosine RNA binding protein 2	- 1.0011033 33	0.49961 776	Down	7.557 E-05
A0A8C0 MFZ6	CLTC	Clathrin heavy chain	- 1.0009866 67	0.49965 8164	Down	1.477 E-07
A0A8I3S 6C2	BRD9	Bromodomain containing 9	- 1.0008433 33	0.49970 7808	Down	0.000 3439
A0A8C0 Q1W3	RPS23	40S ribosomal protein S23	- 1.0005566 67	0.49980 7111	Down	4.049 E-05
A0A8C0 NVL8	NBEAL1	Neurobeachin like 1	1.0009133 33	2.00126 655	Up	9.769 E-06
A0A8I3P 4G8	PIGU	Phosphatidylinositol glycan anchor biosynthesis class U	1.00234	2.00324 6561	Up	2.53E-05
A0A8I3Q J81	PEX16	Peroxisomal membrane protein PEX16	1.0027433 33	2.00380 6686	Up	4.142 E-06
A0A8I3M IP9	FXN	Frataxin, mitochondrial	1.00316	2.00438 5491	Up	3.03E-05
A0A8C0 NPJ3	ANAPC1	Anaphase promoting complex subunit 1	1.00325	2.00451 0535	Up	3.504 E-07
Q8WMU 5	FZD6	Frizzled-6	1.00338	2.00469 1168	Up	0.000 4408
A0A8I3N Q80	TDRD7	Tudor domain-containing protein 7	1.0074433 33	2.01034 5316	Up	2.202 E-05
A0A8C0 N9A0	RARS2	arginine--tRNA ligase	1.0118666 67	2.01651 8535	Up	1.263 E-05
A0A8C0 RJM3	WTAP	Pre-mRNA-splicing regulator WTAP	1.0120166 67	2.01672 8208	Up	1.041 E-05
A0A8C0T Q92	CDCA3	Cell division cycle associated 3	1.0142033 33	2.01978 7244	Up	0.000 4164
A0A8I3P 075	RHOB	Ras homolog family member B	1.0146033 33	2.02034 7325	Up	8.296 E-05
A0A8I3P LM1	EMG1	EMG1 N1-specific pseudouridine methyltransferase	1.0152066 67	2.02119 2409	Up	6.142 E-06
A0A8I3N GN3	FBXL8	F-box and leucine rich repeat protein 8	1.0153766 67	2.02143 059	Up	0.000 1455
A0A8C0 MPC4	CCNH	Cyclin-H	1.0158833 33	2.02214 063	Up	1.125 E-05
A0A8C0P CQ3	-	Arf-GAP with coiled-coil, ANK repeat and PH domain-containing protein	1.0178533 33	2.02490 3749	Up	1.173 E-05
A0A8C0S 5G7	-	Inositol 1,4,5-trisphosphate receptor	1.01792	2.02499 7322	Up	4.617 E-05
Q005W6	BNIP3	BNIP3 (Fragment)	1.0192066 67	2.02680 412	Up	0.000 1461
A0A8C0T 2E2	NOA1	Nitric oxide associated 1	1.0192666 67	2.02688 8414	Up	1.59E-05
A0A8I3S	FASTKD	FAST kinase domains 5	1.0192733	2.02689	Up	3.243

1E6	5		33	778		E-05
A0A8C0QCH6	-	Chromosome 9 open reading frame 78	1.020823333	2.029076605	Up	0.000101
A0A8I3PNJ7	HERC1	HECT and RLD domain containing E3 ubiquitin protein ligase family member 1	1.02089	2.029170371	Up	1.98E-05
A0A8C0LSB4	-	Mitochondrial import inner membrane translocase subunit TIM50	1.02162	2.030197386	Up	1.396E-05
A0A8C0MNQ7	HAUS6	HAUS augmin like complex subunit 6	1.023566667	2.032938634	Up	1.083E-05
A0A8C0PW95	VTA1	Vesicle trafficking 1	1.024046667	2.033615127	Up	8.32E-05
A0A8C0RN40	DUS1L	Dihydrouridine synthase 1 like	1.024313333	2.033991053	Up	4.625E-05
A0A8C0YW64	FAM192A	Family with sequence similarity 192 member A	1.02593	2.036271597	Up	9.846E-05
A0A8I3NGM4	DERPC	DERPC proline and glycine rich nuclear protein	1.027776667	2.038879717	Up	0.0001153
A0A8C0M4F3	-	Tumor protein p63 regulated 1 like	1.02819	2.039463942	Up	2.472E-05
A0A8C0M9S6	COMMD9	COMM domain containing 9	1.029843333	2.041802514	Up	8.523E-05
A0A8C0PN06	-	Activating signal cointegrator 1 complex subunit 1	1.030153333	2.042241295	Up	1.58E-05
A0A8C0MH86	-	TBC1 domain family member 9B	1.032946667	2.046199294	Up	1.639E-05
A0A8I3PMB7	APEH	Acylamino-acid-releasing enzyme	1.033226667	2.046596462	Up	4.836E-05
A0A8C0TXN1	-	Non-structural maintenance of chromosomes element 4	1.033586667	2.047107219	Up	2.649E-05
A0A8C0NHG6	-	Protein prenyltransferase alpha subunit repeat containing 1	1.039813333	2.05596162	Up	0.000172
A0A8C0NEP8	-	HMG box domain-containing protein	1.044893333	2.063213808	Up	2.671E-06
A0A8I3PMB1	RBM5	RNA binding motif protein 5	1.046323333	2.06525988	Up	3.381E-07
A0A8C0RQ09	USP3	Ubiquitin carboxyl-terminal hydrolase	1.04638	2.065341002	Up	0.0001303
A0A8C0MZK8	ASAP1	ArfGAP with SH3 domain, ankyrin repeat and PH domain 1	1.04646	2.065455532	Up	2.718E-07
A0A8I3N8F0	QRSL1	Glutamyl-tRNA(Gln) amidotransferase subunit A, mitochondrial	1.047793333	2.0673653	Up	1.721E-05
A0A8I3PLY1	FRMD8	FERM domain containing 8	1.047873333	2.067479943	Up	3.32E-05
A0A8I3P1H5	FLAD1	FAD synthase	1.04985	2.070314582	Up	2.028E-05
A0A8C0STJ7	CTNNA1	Catenin alpha 1	1.0509	2.071821914	Up	1.54E-05
A0A8I3NRF0	TMED10	Transmembrane p24 trafficking protein 10	1.051483333	2.072659796	Up	1.626E-06
A0A8C0T	POLR2G	DNA-directed RNA	1.05223	2.07373	Up	3.254

141		polymerase II subunit RPB7		2778		E-06
A0A8I3RXL2	PTCD3	Pentatricopeptide repeat domain 3	1.053793333	2.075981135	Up	4.965E-05
A0A8I3SCV8	PCBP4	Poly(rC) binding protein 4	1.05556	2.078524855	Up	6.298E-05
A0A8C0LZ52	ISOC2	Isochorismatase domain containing 2	1.05906	2.08357351	Up	3.664E-05
A0A8I3PYH9	UQCC2	Ubiquinol-cytochrome-c reductase complex assembly factor 2	1.060006667	2.084941156	Up	4.851E-06
A0A8C0NBR4	OGA	O-GlcNAcase	1.060986667	2.086357905	Up	7.818E-05
A0A8C0RE82	FLOT1	Flotillin	1.061486667	2.087081107	Up	1.064E-05
A0A8C0LW15	APOH	Beta-2-glycoprotein 1	1.062133333	2.08801682	Up	0.0073724
A0A8I3NG01	LRRC14	Leucine-rich repeat-containing protein 14	1.062173333	2.088074713	Up	2.91E-05
A0A8C0T6R7	POLR2D	RNA polymerase II subunit D	1.06351	2.090010224	Up	2.912E-05
A0A8C0MJZ2	-	Thioredoxin domain-containing protein	1.065106667	2.092324571	Up	5.412E-05
A0A8C0TUE0	-	Diacylglycerol kinase	1.065786667	2.093311	Up	0.0474403
E2RGI3	SNRPC	U1 small nuclear ribonucleoprotein C	1.065903333	2.093480287	Up	8.188E-07
A0A8C0RXN6	RABGGTA	Geranylgeranyl transferase type-2 subunit alpha	1.06781	2.096248861	Up	6.226E-05
A0A8I3QRD1	SLC27A4	Solute carrier family 27 member 4	1.069016667	2.098002892	Up	7.468E-06
A0A8C0QM77	ASPSCR1	ASPSCR1 tether for SLC2A4, UBX domain containing	1.07086	2.100685226	Up	2.483E-05
A0A8P0TCD4	SLC44A2	Choline transporter-like protein	1.072493333	2.103064844	Up	7.986E-05
A0A8C0MMP3	OSGEP	Phosphatidylinositol-4,5-bisphosphate 4-phosphatase	1.07305	2.103876472	Up	0.0005001
A0A8I3PQV0	CFDP1	Craniofacial development protein 1	1.074006667	2.105272038	Up	0.0002278
A0A8C0QMD7	OGFOD3	2-oxoglutarate and iron dependent oxygenase domain containing 3	1.077123333	2.109824991	Up	4.207E-05
A0A8I3S5W3	SH3RF1	E3 ubiquitin-protein ligase SH3RF1	1.07799	2.111092802	Up	2.005E-06
A0A8C0S0U1	-	Pyroglutamyl-peptidase I	1.078	2.111107435	Up	0.0003886
A0A8P0TLX6	BRK1	BRICK1 subunit of SCAR/WAVE actin nucleating complex	1.079246667	2.112932481	Up	1.942E-05
A0A8C0Z6R0	EXOC3	Exocyst complex component 3	1.07945	2.113230299	Up	1.034E-05
A0A8I3P843	ARNTL	Aryl hydrocarbon receptor nuclear translocator like	1.07997	2.113992121	Up	0.0001032
A0A8C0TQJ2	SBNO1	Strawberry notch homolog 1	1.08206	2.117056834	Up	9.673E-06
A0A8C0P8F1	-	HCLS1 associated protein X-1	1.08217	2.117218258	Up	5.566E-06

A0A8P0S762	ADSL	Adenylosuccinate lyase	1.082506667	2.117712388	Up	7.769E-05
A0A8C0YWU7	MED4	Mediator of RNA polymerase II transcription subunit 4	1.08493	2.121272555	Up	6.786E-06
A0A8C0Q1K8	BCAS3	BCAS3 microtubule associated cell migration factor	1.085963333	2.122792466	Up	0.00011
A0A8C0T506	COL6A5	Collagen type VI alpha 5 chain	1.089573333	2.128110898	Up	4.851E-06
A0A8I3PJA6	EPS15	Epidermal growth factor receptor pathway substrate 15	1.090596667	2.129620947	Up	1.098E-06
A0A8I3NBP1	STK38	Serine/threonine kinase 38	1.093036667	2.133225778	Up	8.513E-05
A0A8C0NM97	CEP85	Centrosomal protein 85	1.096106667	2.137770034	Up	1.248E-07
A0A8I3PUK2	TRIM59	Tripartite motif containing 59	1.096866667	2.138896491	Up	7.68E-06
A0A8P0T476	OXCT1	Succinyl-CoA:3-ketoacid-coenzyme A transferase	1.099676667	2.143066572	Up	3.764E-05
A0A8P0TNU5	MRPS35	Mitochondrial ribosomal protein S35	1.09991	2.143413208	Up	0.0001054
A0A8I3RR04	DMAC2L	ATP synthase subunit s, mitochondrial	1.10014	2.143754946	Up	1.352E-05
A0A8P0SMW7	VAPA	VAMP associated protein A	1.102696667	2.147557362	Up	0.000111
A0A8C0TBI7	-	Lymphocyte antigen 75	1.103233333	2.148356378	Up	2.268E-05
A0A8C0QLV7	KDM4A	[histone H3]-trimethyl-L-lysine(9) demethylase	1.105896667	2.152326083	Up	1.017E-05
A0A8C0MLJ8	GALK2	Galactokinase 2	1.106473333	2.153186572	Up	6.775E-05
A0A8C0QJG4	APAF1	Apoptotic protease-activating factor 1	1.109796667	2.158152282	Up	6.61E-05
A0A8I3RRL3	THG1L	tRNA(His) guanylyltransferase	1.11185	2.161226085	Up	0.0001296
A0A8I3PEV9	PNPLA8	Patatin like phospholipase domain containing 8	1.112243333	2.161815398	Up	5.552E-05
A0A8I3MKT2	RABEP1	Rabaptin, RAB GTPase binding effector protein 1	1.114933333	2.165850005	Up	2.657E-06
A0A8I3RSW2	CPSF4	Cleavage and polyadenylation specificity factor subunit 4	1.117186667	2.169235471	Up	5.73E-06
A0A8I3NI40	ADGRG6	Adhesion G protein-coupled receptor G6	1.119056667	2.172049025	Up	2.106E-05
A0A8I3NQV6	LRRC40	Leucine rich repeat containing 40	1.120393333	2.174062376	Up	5.323E-05
Q00M95	RHBDF2	Inactive rhomboid protein 2	1.12059	2.174358762	Up	3.589E-05
A0A8C0TVZ5	WDR4	tRNA (guanine-N(7)-)-methyltransferase non-catalytic subunit WDR4	1.120813333	2.174695385	Up	2.08E-05
A0A8I3Q3R4	ARAF	non-specific serine/threonine protein kinase	1.130006667	2.188597516	Up	9.423E-06
A0A8I3S	UBL7	Ubiquitin like 7	1.1309966	2.19009	Up	4.409

759			67	9881		E-05
A0A8C0THC4	NR2C2AP	Nuclear receptor 2C2 associated protein	1.135656667	2.197185486	Up	0.000411
A0A8P0PMJ0	USF2	Upstream transcription factor 2, c-fos interacting	1.137216667	2.199562608	Up	1.486E-05
C1IPW3	SLC30A1	SLC30A1 (Fragment)	1.137406667	2.199852305	Up	0.0001055
A0A8I3NHK4	TMEM63B	Transmembrane protein 63B	1.137776667	2.200416562	Up	1.361E-05
A0A8C0RQK2	-	Heparan-alpha-glucosaminide N-acetyltransferase	1.139386667	2.202873524	Up	9.014E-05
A0A8P0SIX9	LOC480510	AA_TRNA_LIGASE_II_ALA domain-containing protein	1.13969	2.203336738	Up	0.0001121
A0A8C0PT27	POC5	Centrosomal protein POC5	1.140146667	2.204034286	Up	0.000283
A0A8I3PTL3	MED13L	Mediator of RNA polymerase II transcription subunit 13	1.141343333	2.205863216	Up	1.183E-05
A0A8I3PCX3	PUM2	Pumilio RNA binding family member 2	1.14193	2.206760405	Up	1.061E-05
A0A8C0TFP8	MRPL4	Mitochondrial ribosomal protein L4	1.14231	2.207341733	Up	2.382E-06
A0A8C0RYB2	KIDINS220	Kinase D interacting substrate 220	1.142933333	2.208295647	Up	8.604E-05
A0A8I3NVZ1	NECTIN1	Nectin cell adhesion molecule 1	1.144583333	2.210822704	Up	1.772E-05
A0A8I3NZG1	HMGN4	High mobility group nucleosomal binding domain 4	1.14655	2.213838529	Up	8.409E-06
A0A8C0T6T3	C12H6orf89	Chromosome 12 C6orf89 homolog	1.14768	2.215573211	Up	9.2E-06
A0A8C0PAT1	VRK2	VRK serine/threonine kinase 2	1.14776	2.215696072	Up	6.597E-06
A0A8I3NRJ1	OSBPL3	Oxysterol-binding protein	1.148546667	2.216904567	Up	1.825E-05
A0A8C0TA12	-	Cyclin-L1	1.149236667	2.217965103	Up	4.825E-05
A0A8I3PZN6	GNA13	G protein subunit alpha 13	1.149696667	2.218672409	Up	2.859E-05
A0A8C0PCW0	DECR1	2,4-dienoyl-CoA reductase 1	1.159853333	2.234347117	Up	5.201E-06
A0A8P0SJW4	GPKOW	G-patch domain and KOW motifs	1.164073333	2.240892332	Up	1.804E-05
A0A8I3Q4Y5	PPP1R14B	Protein phosphatase 1 regulatory subunit 14	1.16927	2.248978704	Up	0.0004554
A0A8C0SUL4	WASHC5	WASH complex subunit 5	1.172246667	2.25362374	Up	3.923E-06
Q1HKN0	COX2	Cytochrome c oxidase subunit 2	1.17351	2.255598049	Up	1.247E-06
A0A8I3PLL0	TAB1	TGF-beta activated kinase 1 (MAP3K7) binding protein 1	1.177553333	2.261928511	Up	9.689E-05
A0A8I3PLP4	CCDC124	Coiled-coil domain containing 124	1.179393333	2.264815194	Up	1.607E-05
A0A8C0T	-	Tripartite motif containing	1.1797866	2.26543	Up	2.526

YQ7		25	67	2753		E-05
A0A8I3NFZ1	UBE3C	HECT-type E3 ubiquitin transferase	1.18093	2.267228816	Up	4.694E-05
A0A8C0NFG8	-	Transmembrane protein 70	1.182526667	2.269739404	Up	1.324E-05
A0A8I3Q0J1	VPS18	Vacuolar protein sorting-associated protein 18 homolog	1.182683333	2.269985895	Up	1.386E-06
A0A8I3NLE2	RHOBTB3	Rho related BTB domain containing 3	1.182953333	2.270410762	Up	1.383E-06
A0A8I3MF38	SGF29	SAGA complex associated factor 29	1.18358	2.27139718	Up	2.227E-06
A0A8C0NCH1	NSD3	Nuclear receptor binding SET domain protein 3	1.184646667	2.273077174	Up	1.317E-05
A0A8I3PC29	EPS8	Epidermal growth factor receptor pathway substrate 8	1.186593333	2.276146368	Up	1.88E-06
A0A8I3S2E2	NTHL1	Endonuclease III-like protein 1	1.187406667	2.277429929	Up	0.0002938
A0A8I3PWI9	WDR13	WD repeat domain 13	1.18777	2.278003557	Up	1.612E-05
A0A8C0MHA3	-	SH3 domain-binding glutamic acid-rich-like protein	1.18917	2.280215219	Up	1.31E-05
A0A8I3MZJ5	PHIP	Pleckstrin homology domain interacting protein	1.18927	2.280373277	Up	1.532E-05
A0A8C0RGZ3	-	Core-binding factor subunit beta	1.189836667	2.281269145	Up	7.352E-05
A0A8I3RYP5	GTPBP10	GTP binding protein 10	1.189863333	2.281311313	Up	5.213E-07
A0A8C0P6B2	SMARCC1	SWI/SNF related, matrix associated, actin dependent regulator of chromatin subfamily c member 1	1.190146667	2.281759387	Up	3.302E-06
A0A8I3N150	TAF2	Transcription initiation factor TFIID subunit 2	1.19271	2.285817146	Up	1.029E-06
A0A8C0MEK6	NFYA	Nuclear transcription factor Y subunit	1.193506667	2.28707974	Up	2.622E-05
A0A8I3SBM5	MAP2K7	Mitogen-activated protein kinase kinase 7	1.194156667	2.288110406	Up	4.308E-05
A0A8I3P4Z6	PANX1	Pannexin	1.19521	2.2897816	Up	0.0002152
A0A8C0ND78	HAS3	Hyaluronan synthase 3	1.199196667	2.296117811	Up	1.551E-05
A0A8C0T6N7	L1CAM	L1 cell adhesion molecule	1.1998	2.297078245	Up	2.301E-05
A0A8C0SDZ3	-	NOP2/Sun RNA methyltransferase 5	1.20378	2.303424003	Up	0.0001027
A0A8C0NPH0	EXTL2	Exostosin like glycosyltransferase 2	1.204833333	2.305106382	Up	9.872E-05
A0A8I3MVX8	PDSS1	Decaprenyl diphosphate synthase subunit 1	1.204993333	2.305362041	Up	3.596E-05
A0A8I3MXM8	CCNC	Cyclin-C	1.205856667	2.306742021	Up	8.294E-07
A0A8P0TCS8	MAP4K2	non-specific serine/threonine protein kinase	1.206466667	2.307717564	Up	0.0003543

A0A8C0P5N0	MRPL34	39S ribosomal protein L34, mitochondrial	1.209116667	2.311960367	Up	0.0001062
A0A8I3MF76	RUFY2	RUN and FYVE domain containing 2	1.21139	2.315606321	Up	6.062E-06
A0A8I3MQX8	MED29	Mediator of RNA polymerase II transcription subunit 29	1.211446667	2.315697276	Up	1.786E-05
A0A8P0S9A2	NOSIP	Nitric oxide synthase-interacting protein	1.211523333	2.315820339	Up	2.69E-06
A0A8I3SCQ8	PDK3	Protein-serine/threonine kinase	1.211873333	2.316382228	Up	1.07E-05
A0A8I3RQ15	UBE2G1	Ubiquitin conjugating enzyme E2 G1	1.212836667	2.317929467	Up	0.0002026
A0A8I3ND46	TUT1	Speckle targeted PIP5K1A-regulated poly(A) polymerase	1.2134	2.318834732	Up	6.939E-06
A0A8C0SAT5	TMEM87B	Transmembrane protein 87B	1.215776667	2.322657882	Up	9.925E-06
A0A8I3NUQ1	ZNF462	Zinc finger protein 462	1.216226667	2.32338247	Up	0.0004024
A0A8C0SX35	N4BP2L2	NEDD4 binding protein 2 like 2	1.217813333	2.325939116	Up	3.391E-05
A0A8I3SCH9	DNAJB2	DnaJ heat shock protein family (Hsp40) member B2	1.218373333	2.326842134	Up	9.851E-07
Q5TJE7	WDR46	WD repeat-containing protein 46	1.218876667	2.327654073	Up	1.166E-05
A0A8I3RWT5	STRBP	Spermatid perinuclear RNA-binding protein	1.219493333	2.32864922	Up	2.898E-05
A0A8I3Q2X8	PTMS	Parathymosin	1.220926667	2.330963909	Up	4.824E-05
A0A8C0RE48	-	Inhibitor of Bruton tyrosine kinase	1.222476667	2.333469591	Up	3.802E-06
A0A8I3QB13	CDCA8	Cell division cycle associated 8	1.223096667	2.334472618	Up	1.985E-05
A0A8C0Z5W1	SERINC3	Serine incorporator 3	1.22327	2.334753111	Up	2.4E-05
A0A8C0TH98	MFSD5	Molybdate-anion transporter	1.224313333	2.336442177	Up	0.0001039
A0A8C0M1A6	GINS3	DNA replication complex GINS protein PSF3	1.226316667	2.339688826	Up	2.785E-05
A0A8C0TEI8	WBP4	WW domain binding protein 4	1.229086667	2.344185385	Up	2.107E-05
A0A8I3RTS5	KLHL5	Kelch like family member 5	1.229433333	2.344748739	Up	1.013E-05
A0A8I3Q294	BCS1L	Mitochondrial chaperone BCS1	1.230186667	2.345973418	Up	7.603E-06
A0A8C0T8P6	-	ATP synthase subunit epsilon, mitochondrial	1.230423333	2.346358295	Up	0.0001037
A0A8C0MDV4	ZNF131	Zinc finger protein 131	1.230846667	2.347046893	Up	4.566E-06
A0A8C0MGX7	-	EPM2A interacting protein 1	1.233316667	2.351068652	Up	9.61E-06
A0A8C0PN02	KRT8	IF rod domain-containing protein	1.233933333	2.352073809	Up	8.437E-06
A0A8C0P5Y9	GPN3	GPN-loop GTPase 3	1.234046667	2.352258588	Up	5.152E-07
A0A8I3M	NOL8	Nucleolar protein 8	1.23492	2.35368	Up	4.872

JN1				2955		E-06
A0A8C0RLU9	-	Low density lipoprotein receptor adaptor protein 1	1.235243333	2.354210516	Up	4.099E-06
A0A8I3PLR3	ABRAXAS2	Abraxas 2, BRISC complex subunit	1.235606667	2.354803483	Up	9.986E-06
A0A8P0TM81	SLC25A25	Solute carrier family 25 member 25	1.238043333	2.358784033	Up	4.971E-05
E2RK33	GATC	Glutamyl-tRNA(Gln) amidotransferase subunit C, mitochondrial	1.239203333	2.360681377	Up	2.03E-06
A0A8C0SM89	TMEM159	Ankyrin repeat and sterile alpha motif domain containing 4B	1.24377	2.368165652	Up	1.205E-05
A0A8C0T0Y3	-	Torsin	1.245723333	2.371374195	Up	0.0002988
A0A8C0SZX4	SLC7A1	AA_permease_C domain-containing protein	1.247513333	2.374318264	Up	0.0004715
E2RP94	DNMBP	Dynamin-binding protein	1.248983333	2.376738753	Up	3.129E-05
A0A8I3S5R5	NR2C2	Nuclear receptor subfamily 2 group C member 2	1.249053333	2.376854076	Up	2.034E-05
A0A8I3QCG5	SLC9A6	Sodium/hydrogen exchanger	1.249223333	2.377134169	Up	0.0002025
A0A8I3N9C7	HAS2	Hyaluronan synthase 2	1.249483333	2.37756261	Up	3.778E-05
A0A8C0TEM8	-	Claudin domain containing 1	1.2506	2.37940359	Up	7.233E-06
A0A8P0N892	PAK4	non-specific serine/threonine protein kinase	1.25241	2.382390655	Up	2.064E-05
A0A8C0TJE0	IKBKG	Inhibitor of nuclear factor kappa B kinase regulatory subunit gamma	1.25248	2.382506252	Up	2.511E-05
A0A8C0QBW2	GFM2	Ribosome-releasing factor 2, mitochondrial	1.253256667	2.383789206	Up	3.142E-05
A0A8C0RQS5	TCP11L1	T-complex 11 like 1	1.25377	2.384637546	Up	6.886E-05
A0A8I3PPA1	ESF1	ESF1 nucleolar pre-rRNA processing protein homolog	1.254616667	2.386037416	Up	8.125E-06
A0A8I3MNW7	RBM23	RNA binding motif protein 23	1.25629	2.388806506	Up	2.5E-05
A0A8C0T445	EXOSC7	Exosome component 7	1.261413333	2.397304774	Up	4.503E-05
A0A8C0LXZ1	HMGCL	Hydroxymethylglutaryl-CoA lyase, mitochondrial	1.262763333	2.399549099	Up	4.39E-05
A0A8I3RQQ2	ARL15	ADP ribosylation factor like GTPase 15	1.265663333	2.404377348	Up	3.28E-06
A0A8C0NQR2	-	Nucleolar protein with MIF4G domain 1	1.2688	2.409610564	Up	3.535E-06
A0A8C0N792	-	Semaphorin 7A (John Milton Hagen blood group)	1.272093333	2.415117421	Up	2.796E-05
A0A8C0NPR2	-	MICOS complex subunit MIC10	1.274246667	2.418724861	Up	0.0002246
A0A8C0R9Z7	PLEKHH3	Pleckstrin homology, MyTH4 and FERM domain containing H3	1.2752	2.420323683	Up	7.57E-05
A0A8C0P	ENPP1	Ectonucleotide	1.27622	2.42203	Up	1.716

JE1		pyrophosphatase/phosphodiesterase 1		5482		E-05
A0A8C0PS52	ADO	2-aminoethanethiol dioxygenase	1.2779	2.424857554	Up	9.923E-06
A0A8C0MFY0	NEK6	NIMA related kinase 6	1.279726667	2.427929729	Up	4.5E-05
A0A8I3Q7G3	SRSF9	Serine and arginine rich splicing factor 9	1.283433333	2.434175746	Up	3.72E-06
A0A8C0TE06	DNAJC19	DnaJ heat shock protein family (Hsp40) member C19	1.284403333	2.435812921	Up	1.217E-05
A0A8C0QE41	-	Protein kinase domain-containing protein	1.28626	2.438949692	Up	5.186E-05
A0A8I3P0F1	ORC5	Origin recognition complex subunit 5	1.28755	2.441131478	Up	7.726E-07
A0A8I3NWT5	GK	glycerol kinase	1.288	2.441893025	Up	9.303E-07
Q5TJG6	BRD2	Bromodomain-containing protein 2	1.28912	2.443789464	Up	8.214E-07
A0A8C0MKF3	ENPP4	bis(5'-adenosyl)-triphosphatase	1.289666667	2.444715641	Up	9.136E-05
A0A8C0SDE6	SPRY1	Protein sprouty homolog 1	1.291553333	2.447914779	Up	7.322E-05
A0A8I3PUZ4	STXBP2	Syntaxin-binding protein 2	1.292733333	2.449917781	Up	1.423E-05
A0A8I3RVK4	MGAT4B	Alpha-1,3-mannosyl-glycoprotein 4-beta-N-acetylglucosaminyltransferase B	1.294966667	2.453713261	Up	2.721E-06
A0A8C0LTQ3	ARID4A	AT-rich interaction domain 4A	1.296456667	2.456248739	Up	1.441E-05
A0A8C0LUQ6	NEMF	Nuclear export mediator factor	1.298133333	2.459104994	Up	4.253E-06
A0A8P0P9W5	MACROD1	Mono-ADP ribosylhydrolase 1	1.300236667	2.462692786	Up	3.908E-06
A0A8C0REP0	-	protein-tyrosine-phosphatase	1.30043	2.463022829	Up	2.274E-05
A0A8P0NYY7	KIAA0825	KIAA0825	1.304206667	2.469478942	Up	1.217E-05
A0A8C0LZL9	-	DNA-directed RNA polymerase subunit	1.306536667	2.473470454	Up	9.487E-07
A0A8I3PPV6	LOC119867035	Intersectin-1	1.308173333	2.476278077	Up	5.306E-06
A0A8P0SEA7	GMDS	GDP-mannose 4,6-dehydratase	1.308896667	2.477519936	Up	5.482E-05
A0A8C0NHZ0	-	Pleckstrin 2	1.311446667	2.481902888	Up	5.101E-06
A0A8C0M3S3	EMC10	ER membrane protein complex subunit 10	1.312883333	2.484375651	Up	2.055E-05
A0A8I3S4G7	ELP5	Elongator complex protein 5	1.31306	2.484679896	Up	0.0004896
A0A8C0PFR9	-	Phosphorylated adapter RNA export protein	1.313666667	2.485724947	Up	8.647E-06
A0A8C0PCR2	ANKRD28	Ankyrin repeat domain 28	1.315843333	2.489478116	Up	5.912E-05
A0A8C0NXZ7	-	Cytochrome b-245 light chain	1.316596667	2.490778388	Up	1.816E-05

A0A8P0TA39	HSPE1	MOB family member 4, phocein	1.316896667	2.491296385	Up	6.021E-06
A0A8C0MZT7	-	GRAM domain containing 4	1.318493333	2.494055091	Up	0.0001976
A0A8I3NPA0	GUSB	Beta-glucuronidase	1.321073333	2.49851925	Up	2.598E-05
A0A8C0RTW9	-	ASH1 like histone lysine methyltransferase	1.321883333	2.499922435	Up	3.698E-05
A0A8C0RIW5	RPS6KA4	Ribosomal protein S6 kinase	1.32264	2.501233942	Up	4.529E-05
A0A8C0RXC9	MRPL20	Mitochondrial ribosomal protein L20	1.3246	2.504634349	Up	9.364E-06
A0A8I3P0Y1	ATP7A	P-type Cu(+) transporter	1.325113333	2.505525695	Up	0.0027829
A0A8C0PAE6	-	Mitochondrial translational initiation factor 3	1.3263	2.507587425	Up	6.635E-05
A0A8I3QJR6	IFT122	Intraflagellar transport protein 122 homolog	1.32714	2.509047877	Up	2.739E-05
A0A8I3MXU7	SMYD3	SET and MYND domain containing 3	1.328973333	2.512238326	Up	0.0004904
A0A8I3Q3L9	GLE1	mRNA export factor GLE1	1.331306667	2.516304765	Up	3.112E-06
A0A8C0Q4T5	VPS33B	VPS33B late endosome and lysosome associated	1.334723333	2.522271074	Up	2.261E-06
A0A8P0SYC3	FARP2	FERM, ARH/RhoGEF and pleckstrin domain protein 2	1.336626667	2.525600877	Up	8.991E-07
A0A8I3MME1	DDX50	RNA helicase	1.337053333	2.526347916	Up	4.662E-06
A0A8I3PM85	GLTP	Glycolipid transfer protein	1.337246667	2.52668649	Up	2.333E-05
A0A8I3MX99	DNAJC21	DnaJ heat shock protein family (Hsp40) member C21	1.337846667	2.527737528	Up	2.173E-05
A0A8I3PSP7	NRAS	NRAS proto-onco, GTPase	1.340453333	2.532308782	Up	1.739E-06
A0A8C0MXI9	-	GIT ArfGAP 1	1.340683333	2.532712525	Up	5.651E-07
A0A8I3NAV1	PPP4R3B	Protein phosphatase 4 regulatory subunit 3B	1.34152	2.534181755	Up	1.774E-06
A0A8I3N0E3	STARD7	StAR related lipid transfer domain containing 7	1.34365	2.537925993	Up	1.129E-06
A0A8C0PDE9	-	Heat shock protein beta-8	1.347863333	2.545348738	Up	5.18E-05
A0A8C0S6E6	PPCS	Phosphopantothenoylcysteine synthetase	1.349426667	2.548108424	Up	9.935E-05
A0A8I3MVP1	HDAC1	Histone deacetylase 1	1.35233	2.553241496	Up	3.032E-07
A0A8C0THK6	-	SIN3 transcription regulator family member A	1.35234	2.553259193	Up	1.881E-06
A0A8C0NB96	ORC2	Origin recognition complex subunit 2	1.353516667	2.555342489	Up	9.037E-06
A0A8P0PBG6	TENM4	Teneurin transmembrane protein 4	1.353943333	2.556098325	Up	0.0001219
A0A8C0M8C1	-	Caspase recruitment domain family member 6	1.355223333	2.558367174	Up	1.467E-05
A0A8C0N0C8	NT5C3A	5'-nucleotidase, cytosolic IIIA	1.355836667	2.559455045	Up	2.202E-05

A0A8C0PA43	GTF3C1	General transcription factor IIIC subunit 1	1.361096667	2.568803733	Up	1.39E-06
A0A8I3RQ44	CREBBP	histone acetyltransferase	1.364166667	2.574275869	Up	5.045E-08
A0A8I3PJP2	SPRYD3	SPRY domain containing 3	1.364896667	2.575578776	Up	5.343E-05
A0A8C0YUK2	MPP5	Protein PALS1	1.365836667	2.577257463	Up	5.775E-06
A0A8C0MLX5	CEP152	Centrosomal protein 152	1.376706667	2.596749172	Up	4.744E-05
A0A8I3PGQ9	ITM2C	Integral membrane protein 2	1.37731	2.597835357	Up	1.333E-05
A0A8C0TJ29	-	Lysophosphatidylcholine acyltransferase 4	1.377526667	2.598225534	Up	3.391E-05
A0A8C0QN38	LOC102151856	ATP synthase subunit f, mitochondrial	1.380006667	2.602695738	Up	3.681E-06
A0A8I3S7L0	TMEM168	Transmembrane protein 168	1.382276667	2.606794158	Up	4.082E-05
A0A8C0MHY6	-	Sterol carrier protein 2	1.38375	2.609457672	Up	5.696E-05
A0A8C0PRI8	-	Cysteine protease	1.385653333	2.612902575	Up	2.96E-05
A0A8P0SN39	INPP5K	inositol-polyphosphate 5-phosphatase	1.38889	2.618771167	Up	9.592E-05
A0A8C0SU29	-	Nedd4 family interacting protein 2	1.390166667	2.62108959	Up	3.198E-05
A0A8C0MZB8	-	GPI ethanolamine phosphate transferase 1	1.390686667	2.622034497	Up	0.0001341
A0A8C0PSU5	POU2F1	POU domain protein	1.393146667	2.626509252	Up	2.909E-06
A0A8C0MDD9	GOLGA1	Golgin A1	1.3939	2.627881097	Up	6.045E-07
A0A8I3PYL7	DDX51	DEAD-box helicase 51	1.394293333	2.628597655	Up	3.721E-06
A0A8I3Q1R9	RINT1	RAD50 interactor 1	1.395323333	2.63047499	Up	3.804E-06
A0A8P0SR90	TASOR	Transcription activation suppressor	1.397983333	2.635329459	Up	3.798E-06
A0A8C0NUY1	TELO2	Telomere length regulation protein TEL2 homolog	1.402103333	2.6428661	Up	1.143E-05
A0A8I3N7P3	CHD1L	Chromodomain helicase DNA binding protein 1 like	1.403056667	2.644613084	Up	1.998E-05
A0A8C0RAL2	SC5D	Sterol-C5-desaturase	1.40336	2.645169185	Up	3.728E-06
A0A8C0SRB0	UPF3B	UPF3B regulator of nonsense mediated mRNA decay	1.404773333	2.647761789	Up	1.369E-06
A0A8C0N9H8	RPF1	Ribosome production factor 1 homolog	1.404893333	2.647982033	Up	1.195E-05
A0A8C0S9K8	KRT13	IF rod domain-containing protein	1.408773333	2.65511313	Up	3.082E-05
A0A8I3PKL0	YBX3	Y-box binding protein 3	1.408826667	2.655211286	Up	9.301E-07
A0A8C0M2H6	COG1	Conserved oligomeric Golgi complex subunit 1	1.4093	2.656082576	Up	9.662E-07
A0A8I3RV90	NFXL1	Nuclear transcription factor, X-box binding like 1	1.41107	2.659343245	Up	0.0001244

A0A8I3Q4C6	INTS12	Integrator complex subunit 12	1.411696667	2.660498641	Up	2.396E-06
A0A8I3NFL6	MRPS24	28S ribosomal protein S24, mitochondrial	1.4119	2.660873638	Up	3.23E-05
A0A8I3N558	SUPV3L1	ATP-dependent RNA helicase SUPV3L1, mitochondrial	1.412733333	2.662411063	Up	5.972E-06
A0A8P0T7D1	NARS2	asparagine--tRNA ligase	1.413433333	2.663703186	Up	4.153E-06
A0A8C0SWE1	EXOC1	Exocyst complex component 1	1.416743333	2.669821582	Up	1.331E-05
A0A8C0RLU4	GPRC5A	G protein-coupled receptor class C group 5 member A	1.417916667	2.671993812	Up	1.101E-05
A0A8C0T7Q4	ZDHHC3	Palmitoyltransferase	1.419966667	2.675793285	Up	1.259E-05
A0A8C0MKD5	-	Serine/threonine-protein phosphatase 1 regulatory subunit 10	1.420366667	2.676535275	Up	5.212E-07
A0A8I3P1M1	VEZF1	Vascular endothelial zinc finger 1	1.421113333	2.677920874	Up	6.368E-06
A0A8I3N6U5	COP1	COP1 E3 ubiquitin ligase	1.42431	2.683861084	Up	4.728E-05
A0A8I3MXF1	MRPL24	Mitochondrial ribosomal protein L24	1.427996667	2.6907282	Up	5.244E-08
A0A8C0REJ6	TAF1	Transcription initiation factor TFIID subunit	1.430336667	2.695096007	Up	6.011E-06
A0A8C0M2F1	ACOX1	Acyl-coenzyme A oxidase	1.431783333	2.697799878	Up	1.849E-06
A0A8C0M733	DCTN4	Dynactin subunit 4	1.432393333	2.698940802	Up	3.505E-06
A0A8C0MD43	MCL1	Induced myeloid leukemia cell differentiation protein Mcl-1 homolog	1.433406667	2.700837175	Up	2.674E-05
A0A8C0RZM6	RRP36	rRNA biogenesis protein RRP36	1.43595	2.705602691	Up	1.848E-05
A0A8C0RPE5	EIF4A2	RNA helicase	1.4384	2.710201278	Up	4.682E-06
A0A8I3NBD9	BFAR	Bifunctional apoptosis regulator	1.441703333	2.716413926	Up	1.013E-06
A0A8P0P5V0	SFXN2	Sidoreflexin	1.441883333	2.716752864	Up	1.916E-05
A0A8I3S0L9	PPAN	Peter pan homolog	1.44339	2.719591565	Up	1.22E-05
A0A8I3RWL2	RECQL4	DNA helicase	1.443943333	2.720634841	Up	7.123E-08
A0A8C0P3F8	-	Transforming growth factor beta receptor associated protein 1	1.444626667	2.721923776	Up	5.645E-06
A0A8C0SB88	-	Protein S100	1.446226667	2.724944161	Up	7.876E-06
Q29476	SULT1A1	Sulfotransferase 1A1	1.4471	2.726594201	Up	6.057E-05
A0A8I3NL10	JUP	Junction plakoglobin	1.448086667	2.728459571	Up	1.515E-05
A0A8C0M0D6	CENPP	Centromere protein P	1.453483333	2.73868499	Up	3.239E-06
A0A8C0	-	4-hydroxyphenylpyruvate	1.4536266	2.73895	Up	3.738

RLM5		dioxygenase	67	7095		E-06
A0A8C0N2P3	-	Round spermatid basic protein 1 like	1.45692	2.745216631	Up	4.838E-06
A0A8I3S5G6	TNIP3	TNFAIP3 interacting protein 3	1.458576667	2.748370812	Up	2.653E-05
A0A8I3RWZ5	LSM7	LSM7 homolog, U6 small nuclear RNA and mRNA degradation associated	1.459406667	2.749952438	Up	2.106E-06
A0A8I3NXV8	SPATA5	Spermatosis associated 5	1.460003333	2.751089993	Up	2.601E-05
A0A8I3NAB3	LOC119877163	GPN-loop GTPase	1.462096667	2.755084689	Up	6.43E-06
A0A8P0SW10	SMARCAD1	SWI/SNF-related matrix-associated actin-dependent regulator of chromatin subfamily A containing DEAD/H box 1	1.46559	2.761763918	Up	2.244E-05
A0A8P0P6R7	POT1	Protection of telomeres protein 1	1.46578	2.762127661	Up	4.393E-07
A0A8I3S8E8	MAP2	Microtubule-associated protein	1.46895	2.768203492	Up	3.306E-06
A0A8C0RZP2	AFF4	AF4/FMR2 family member 4	1.47229	2.774619616	Up	4.707E-05
A0A8P0NJK0	ATPAF2	ATP synthase mitochondrial F1 complex assembly factor 2	1.478323333	2.786247338	Up	2.077E-05
A0A8C0S4I9	HSD17B7	3beta-hydroxysteroid 3-dehydrogenase	1.47932	2.788172845	Up	3.747E-06
A0A8I3NB95	PAK1	non-specific serine/threonine protein kinase	1.479643333	2.788797794	Up	1.331E-05
A0A8C0RKE4	SMYD4	SET and MYND domain-containing protein 4	1.481516667	2.792421388	Up	3.944E-06
A0A8P0TK54	ATP9A	Phospholipid-transporting ATPase	1.482746667	2.794803141	Up	2.181E-05
A0A8P0SCA8	DHX37	DEAH-box helicase 37	1.484726667	2.79864145	Up	4.772E-07
A0A8P0PRX8	STX3	Syntaxin 3	1.488766667	2.80648951	Up	9.358E-06
A0A8C0Z6E1	-	Helicase with zinc finger 2	1.488843333	2.806638654	Up	4.485E-06
A0A8C0SSJ3	TAF1A	TATA-box binding protein associated factor, RNA polymerase I subunit A	1.4937	2.816102801	Up	3.242E-06
A0A8C0PD23	MKRN2	E3 ubiquitin-protein ligase makorin-2	1.49418	2.817039904	Up	6.662E-06
A0A8C0N8V6	RPL7L1	Ribosomal protein L7 like 1	1.497106667	2.822760382	Up	2.098E-06
A0A8P0TQ05	RFX1	Regulatory factor X1	1.499193333	2.826846084	Up	9.54E-06
A0A8I3NY28	CDK13	Cyclin dependent kinase 13	1.500373333	2.829159146	Up	2.769E-06
A0A8P0NHE2	FTH1	Ferritin	1.50123	2.830839588	Up	6.864E-05
A0A8C0PFH2	-	Zinc finger protein 281	1.504196667	2.836666736	Up	1.732E-05
A0A8C0	-	Eukaryotic translation	1.50742	2.84301	Up	5.291

Q598		initiation factor 4E binding protein 2		1629		E-05
A0A8I3PNU5	IP6K1	Kinase	1.509366667	2.846850369	Up	7.382E-05
A0A8C0S082	-	Phospholipid-transporting ATPase	1.510183333	2.848462342	Up	2.585E-05
A0A8C0QB65	-	H/ACA ribonucleoprotein complex non-core subunit NAF1	1.510466667	2.849021811	Up	2.157E-06
A0A8C0Z794	RMDN3	Regulator of microtubule dynamics 3	1.5127	2.853435594	Up	1.758E-06
A0A8C0NX39	CDK5RAP1	CDK5 regulatory subunit associated protein 1	1.514363333	2.856727317	Up	0.0001001
A0A8I3N9C2	MLLT1	MLLT1 super elongation complex subunit	1.515146667	2.858278842	Up	2.505E-05
A0A8C0PRS9	-	KH homology domain-containing protein 4	1.515493333	2.858965743	Up	5.996E-05
A0A8C0NTC2	POLR3D	RNA polymerase III subunit D	1.516253333	2.86047222	Up	3.364E-05
A0A8C0Q891	ABCB8	ATP binding cassette subfamily B member 8	1.51691	2.861774508	Up	1.682E-06
A0A8C0S441	-	CDK5 regulatory subunit associated protein 2	1.51721	2.862369659	Up	6.011E-09
A0A8C0MMH5	MIGA1	Mitoguardin 1	1.519163333	2.866247782	Up	6.609E-05
A0A8I3MXA7	KIAA1522	KIAA1522	1.520916667	2.869733302	Up	1.847E-06
A0A8C0PLI1	-	Centromere protein E	1.521796667	2.871484286	Up	6.575E-05
A0A8I3RZ79	ATF1	Activating transcription factor 1	1.523436667	2.874750334	Up	2.605E-07
A0A8C0PJR6	AP1B1	AP complex subunit beta	1.525153333	2.878173043	Up	1.141E-07
A0A8I3S1K3	PHF23	PHD finger protein 23	1.525553333	2.878971153	Up	1.081E-06
A0A8I3Q4R8	ADA2	Haloacid dehalogenase like hydrolase domain containing 5	1.526196667	2.880255244	Up	1.114E-06
A0A8I3NBW4	OSBPL5	Oxysterol-binding protein	1.527766667	2.883391362	Up	8.095E-06
A0A654IEC4	GJA1	Gap junction protein	1.527826667	2.883511281	Up	7.503E-06
A0A8I3PW24	ATP2C1	Calcium-transporting ATPase	1.531593333	2.891049546	Up	1.687E-05
A0A8C0MBM4	-	Exosome component 5	1.534696667	2.89727508	Up	2.411E-06
A0A8I3PS84	MPV17L2	MPV17 mitochondrial inner membrane protein like 2	1.537113333	2.902132389	Up	2.181E-05
A0A8C0MKN1	-	Serine and arginine rich splicing factor 4	1.537643333	2.903198735	Up	2.592E-06
A0A8C0M6S0	VPS41	Vacuolar protein sorting-associated protein 41 homolog	1.539383333	2.906702326	Up	5.383E-06
A0A8C0N0N9	DBR1	Debranching RNA lariats 1	1.539623333	2.907185912	Up	6.042E-06
A0A8I3PXE6	H1-3	H1.3 linker histone, cluster member	1.54174	2.911454353	Up	4.818E-06

A0A8C0YYY4	-	Protein phosphatase 1 regulatory subunit 14	1.54343	2.914866884	Up	3.519E-05
A0A8I3ND47	ASRGL1	Isoaspartyl peptidase/L-asparaginase	1.54393	2.915877275	Up	1.166E-05
A0A8I3RXI5	MYO18A	Myosin XVIIIA	1.546613333	2.921305693	Up	1.95E-07
A0A8I3P6S8	DUSP11	Dual specificity phosphatase 11	1.548163333	2.924445966	Up	2.835E-05
A0A8I3N0E4	ANKMY2	Ankyrin repeat and MYND domain containing 2	1.553646667	2.935582225	Up	3.405E-06
A0A8C0TIR5	ESPL1	separase	1.55623	2.940843476	Up	2.504E-06
A0A8I3PAK3	RALA	small monomeric GTPase	1.556933333	2.942277526	Up	1.209E-06
A0A8C0TAR3	LOC489372	Histone H2A	1.557996667	2.944446921	Up	2.403E-05
A0A8C0Q6A6	RAB4A	Ras-related protein Rab-4	1.558493333	2.94546076	Up	7.034E-06
A0A8C0NNT3	FIP1L1	Pre-mRNA 3'-end-processing factor FIP1	1.561863333	2.952349122	Up	9.019E-06
Q8WMD0	COMMD1	COMM domain-containing protein 1	1.56683	2.962530486	Up	1.676E-05
A0A8P0NGT1	ATP5J2	Pentatricopeptide repeat domain 1	1.566896667	2.962667387	Up	1.714E-07
A0A8C0RMB3	MAFG	Transcription factor MafG	1.5673	2.963495774	Up	5.238E-07
A0A8I3QQ94	HDAC6	Histone deacetylase 6	1.570846667	2.970790081	Up	3.23E-05
A0A8I3PNP6	PPM1A	protein-serine/threonine phosphatase	1.571426667	2.971984654	Up	3.985E-05
A0A8C0TU86	-	tRNA methyltransferase 10 homolog C	1.57242	2.974031648	Up	1.396E-05
A0A8P0TUV0	RAB33B	RAB33B, member RAS oncogene family	1.576826667	2.983129622	Up	5.394E-06
A0A8C0SHG4	-	Family with sequence similarity 210 member A	1.576926667	2.983336404	Up	1.672E-05
A0A8C0S4Z5	ELF2	E74 like ETS transcription factor 2	1.58014	2.989988633	Up	4.736E-06
A0A8C0SNF6	UGCG	ceramide glucosyltransferase	1.593243333	3.017269021	Up	2.883E-06
A0A8C0S3N1	-	General transcription and DNA repair factor IIH helicase subunit XPD	1.59337	3.017533944	Up	1.442E-05
A0A8C0S2H0	-	UHRF1 binding protein 1 like	1.59523	3.02142682	Up	2.255E-06
A0A8C0SQT1	-	Zinc finger CCCH-type containing 13	1.595266667	3.021503612	Up	3.294E-07
A0A8P0NX16	LOC100856786	Transient receptor potential cation channel subfamily V member 4	1.595836667	3.022697625	Up	1.05E-05
A0A8I3N3C5	TENT2	Terminal nucleotidyltransferase 2	1.596553333	3.02419954	Up	7.983E-05
A0A8P0SN60	PKMYT1	Protein kinase, membrane associated tyrosine/threonine 1	1.599763333	3.030935883	Up	2.963E-06
A0A8P0PQ17	USP33	ubiquitinyl hydrolase 1	1.601533333	3.034656731	Up	6.716E-06

A0A8C0PIU6	GPNMB	Glycoprotein nmb	1.60478	3.041493667	Up	1.196E-05
A0A8C0MB62	-	Scm like with four mbt domains 1	1.60479	3.041514749	Up	0.0003948
A0A8I3MA26	ABHD17B	Abhydrolase domain containing 17B, depalmitoylase	1.60511	3.042189453	Up	8.958E-07
A0A8I3NXF5	ZC3H7A	Zinc finger CCCH-type containing 7A	1.605966667	3.04399643	Up	5.91E-06
A0A8C0PZV1	-	MutS homolog 3	1.610093333	3.052715903	Up	6.411E-08
A0A8C0RZS9	ATP8B1	Phospholipid-transporting ATPase	1.613123333	3.059134064	Up	7.679E-06
A0A8I3RTH6	SEPTIN10	Septin	1.613243333	3.059388527	Up	7.66E-07
A0A8P0NRK2	COG3	Conserved oligomeric Golgi complex subunit 3	1.613873333	3.0607248	Up	0.0003062
A0A8C0TVB3	HJURP	Holliday junction recognition protein	1.61484	3.062776303	Up	0.0132192
A0A8C0NAW1	AFMID	Kynurenine formamidase	1.616253333	3.065778215	Up	3.553E-06
A0A8I3S988	CHTF18	Chromosome transmission fidelity factor 18	1.61653	3.066366198	Up	7.688E-07
A0A8I3N884	FANCD2	FA complementation group D2	1.623103333	3.080369321	Up	3.345E-07
A0A8C0PNI8	OTUD6B	ubiquitinyl hydrolase 1	1.623526667	3.081273334	Up	2.147E-05
A0A8C0Z3E3	ILKAP	ILK associated serine/threonine phosphatase	1.625173333	3.084792252	Up	9.899E-06
A0A8C0NUH2	DNAJC22	DnaJ homolog subfamily C member 22	1.625446667	3.085376753	Up	5.074E-06
A0A8I3P8K1	GLCE	heparosan-N-sulfate-glucuronate 5-epimerase	1.626203333	3.0869954	Up	1.037E-05
A0A8I3NGX1	ZNF48	Zinc finger protein 48	1.629036667	3.09306396	Up	1.553E-06
A0A8I3Q0U0	CCPG1	Cell cycle progression 1	1.630256667	3.095680684	Up	4.669E-06
A0A8I3S6K6	TAOK3	TAO kinase 3	1.630963333	3.097197394	Up	5.654E-06
A0A8I3P2Z5	MED27	Mediator of RNA polymerase II transcription subunit 27	1.630983333	3.09724033	Up	1.435E-06
A0A8P0NQP9	KMT2D	[histone H3]-lysine(4) N-methyltransferase	1.631603333	3.098571659	Up	7.219E-07
A0A8C0QG53	RABGEF1	RAB guanine nucleotide exchange factor 1	1.636923333	3.110018869	Up	2.519E-06
A0A8I3NB06	LOC102151295	Transcription and mRNA export factor ENY2	1.6378	3.111909274	Up	4.187E-06
A0A8C0PMH9	-	RNA helicase	1.637986667	3.112311942	Up	3.135E-06
A0A8P0NM07	CSF1	Colony stimulating factor 1	1.64181	3.120570921	Up	0.0002928
A0A8C0MAH7	-	BCL9 like	1.642293333	3.121616553	Up	4.431E-06
A0A8C0N1M4	PRPF38A	Pre-mRNA-splicing factor 38A	1.643113333	3.123391324	Up	2.746E-07

A0A8C0RK65	DDX49	RNA helicase	1.64442	3.1262215	Up	1.03E-06
A0A8C0RWM1	-	Mitochondrial import inner membrane translocase subunit TIM23	1.644906667	3.127276251	Up	1.568E-05
A0A8C0SK63	CLP1	Polyribonucleotide 5'-hydroxyl-kinase Clp1	1.65033	3.139054332	Up	1.815E-05
A0A8I3MHQ2	ME2	Malic enzyme	1.65496	3.149144592	Up	3.8E-06
A0A8I3PRW4	MID1IP1	MID1 interacting protein 1	1.654993333	3.149217354	Up	0.0001047
A0A8C0Z3R1	PI4KA	1-phosphatidylinositol 4-kinase	1.659476667	3.159019115	Up	4.385E-06
A0A8C0NYS3	KRT79	IF rod domain-containing protein	1.663063333	3.166882484	Up	0.0307642
A0A8C0MJA1	-	Jumonji domain containing 1C	1.666983333	3.175499039	Up	2.878E-06
A0A8I3RXW1	ZNF771	Zinc finger protein 771	1.668676667	3.179228403	Up	2.449E-06
A0A8I3NLA6	FOXP4	Forkhead box P4	1.66895	3.179830797	Up	2.118E-06
A0A8C0TCX2	-	Pyruvate dehyrogenase phosphatase catalytic subunit 1	1.66912	3.180205515	Up	4.087E-06
A0A8C0M498	-	PHD finger protein 2	1.67534	3.193946174	Up	0.0010843
A0A8I3MBN5	EPHB4	receptor protein-tyrosine kinase	1.68125	3.20705701	Up	1.2E-05
A0A8C0SD09	GOSR2	Golgi SNAP receptor complex member 2	1.68372	3.21255243	Up	7.119E-06
Q5TJE5	RGL2	Ral guanine nucleotide dissociation stimulator-like 2	1.68535	3.216184119	Up	9.091E-06
A0A8I3P0D7	DIDO1	Death inducer-obliterator 1	1.693866667	3.235226381	Up	1.879E-06
A0A8I3PJ84	KRT18	Keratin 18	1.6947	3.237095661	Up	3.471E-05
A0A8I3NS56	OSBPL7	Oxysterol-binding protein	1.694736667	3.237177934	Up	2.913E-06
A0A8C0RR88	YTHDF1	YTH N6-methyladenosine RNA binding protein 1	1.695243333	3.238315013	Up	3.592E-06
A0A8C0RCU5	-	Phosphate transporter	1.695516667	3.238928603	Up	1.23E-06
A0A8I3PM35	TNS2	Tensin 2	1.695533333	3.238966021	Up	5.688E-07
A0A8I3N5B1	TTL	Tubulin tyrosine ligase	1.696313333	3.240717657	Up	3.582E-05
A0A8C0MVC6	ARMT1	Sugar phosphate phosphatase	1.698533333	3.245708269	Up	1.507E-05
A0A8I3PGG7	HMG20A	High mobility group 20A	1.69997	3.248942025	Up	3.234E-06
A0A8C0SS75	LOC403555	SET translocation	1.70277	3.255253734	Up	5.908E-06
A0A8I3Q180	LOC102153616	Caspase recruitment domain-containing protein 8	1.707813333	3.266653273	Up	4.653E-06
A0A8C0P8T9	C24H20orf27	Chromosome 24 C20orf27 homolog	1.71267	3.277668616	Up	0.0002171

A0A8C0PEU5	XPO6	Exportin 6	1.719506667	3.293237745	Up	8.965E-07
A0A8C0Q9R7	ACOT8	Acyl-CoA thioesterase 8	1.719933333	3.294211841	Up	0.0003364
A0A8C0MMY1	MED20	Mediator of RNA polymerase II transcription subunit 20	1.726716667	3.309737196	Up	4.541E-06
A0A8C0QB01	-	VPS37C subunit of ESCRT-I	1.73073	3.318957142	Up	1.514E-05
A0A8I3PZP9	DPH2	2-(3-amino-3-carboxypropyl)histidine synthase subunit 2	1.732573333	3.323200488	Up	1.892E-05
A0A8C0TWF6	-	Methionyl-tRNA formyltransferase, mitochondrial	1.73319	3.324621263	Up	1.286E-05
A0A8C0Q9B9	SLC25A32	Solute carrier family 25 member 32	1.738806667	3.337589829	Up	8.585E-07
A0A8I3N6Y7	KPNA7	Importin subunit alpha	1.73904	3.338129676	Up	2.254E-06
A0A8C0N0L2	-	FRA10A associated CGG repeat 1	1.74553	3.353180164	Up	5.238E-05
A0A8C0T8Q9	-	Cell proliferation regulating inhibitor of protein phosphatase 2A	1.750183333	3.364013122	Up	8.329E-06
A0A8C0T8R6	SLC25A44	Solute carrier family 25 member 44	1.751546667	3.367193586	Up	2.05E-05
A0A8I3N1W2	CSNK1D	Casein kinase 1 delta	1.75575	3.377018306	Up	6.864E-07
A0A8C0Q280	CENPQ	Centromere protein Q	1.75619	3.378048402	Up	0.0026839
A0A8C0PP00	-	Patatin like phospholipase domain containing 6	1.76031	3.387709107	Up	4.693E-05
A0A8P0NEP9	KMT2A	Histone-lysine N-methyltransferase	1.7627	3.393325911	Up	2.148E-06
A0A8C0S0R3	-	Iron-sulfur cluster assembly factor IBA57	1.76925	3.408767023	Up	2.718E-06
A0A8C0S928	-	Ubiquinone biosynthesis protein	1.769296667	3.408877288	Up	8.448E-06
A0A8I3NMV1	TRIM32	Tripartite motif containing 32	1.76973	3.409901345	Up	3.159E-07
A0A8P0PI53	LGALSL	Galectin	1.77022	3.411059688	Up	1.011E-05
A0A8C0TTN5	-	F-box protein 21	1.773096667	3.417867967	Up	0.0001134
A0A8C0ME21	-	S-adenosylmethionine decarboxylase proenzyme	1.775016667	3.42241964	Up	1.03E-05
A0A8I3MNK8	EEF2K	Eukaryotic elongation factor 2 kinase	1.779006667	3.43189798	Up	3.353E-05
Q1HE58	RAB27A	Ras-related protein Rab-27A	1.77908	3.432072431	Up	4.961E-06
Q32KI4	ARSB	Arylsulfatase B	1.782706667	3.440710875	Up	2.076E-05
A0A8I3PCL3	NECAP1	NECAP endocytosis associated 1	1.78513	3.446495186	Up	2.88E-05
A0A8C0RE52	-	Oxidized purine nucleoside triphosphate hydrolase	1.785443333	3.447243798	Up	2.168E-05
A0A8C0L	DHX8	RNA helicase	1.7933233	3.46612	Up	9.606

YS1			33	4158		E-08
A0A8C0SPD1	-	NFKB activating protein	1.794163333	3.468142874	Up	5.811E-06
A0A8I3MRX2	SEC16A	Protein transport protein sec16	1.796476667	3.473708435	Up	4.718E-07
A0A8I3RYK4	SLC7A6	Solute carrier family 7 member 6	1.797466667	3.476092966	Up	0.0003881
A0A8I3Q3T8	NCSTN	Nicastrin	1.801466667	3.485744115	Up	1.652E-05
A0A8C0TGC1	ARMH3	Armadillo like helical domain containing 3	1.806903333	3.49890461	Up	5.375E-07
A0A8I3P057	DDX55	DEAD-box helicase 55	1.816086667	3.521247579	Up	5.186E-07
A0A8C0MZV1	-	RP9 pre-mRNA splicing factor	1.817973333	3.525855459	Up	1.981E-06
A0A8I3NDE7	PBK	Protein kinase domain-containing protein	1.821366667	3.534158312	Up	6.045E-06
A0A8I3MCX5	TAOK2	TAO kinase 2	1.826266667	3.54618221	Up	3.447E-08
A0A8C0S7U9	-	Negative elongation factor complex member B	1.826943333	3.547845864	Up	1.864E-06
A0A8I3Q0N2	DDT	D-dopachrome decarboxylase	1.829493333	3.554122317	Up	0.0156772
A0A8P0PKT0	MED21	Mediator of RNA polymerase II transcription subunit 21	1.831363333	3.558732105	Up	2.553E-06
A0A8C0QEE5	UQCC1	Ubiquinol-cytochrome c reductase complex assembly factor 1	1.836453333	3.571309911	Up	1.77E-05
A0A8I3N920	NHSL1	NHS like 1	1.838203333	3.575644565	Up	1.991E-06
A0A8C0RAV1	-	Ras-related protein Rap-2a	1.841546667	3.583940451	Up	0.0001947
A0A8C0PR47	FUCA1	Alpha-L-fucosidase	1.84175	3.584445607	Up	1.292E-06
A0A8I3PNL5	INTS14	Integrator complex subunit 14	1.841996667	3.585058515	Up	9.93E-07
A0A8C0RGR7	DICER1	Dicer 1, ribonuclease III	1.843243333	3.588157787	Up	1.158E-05
A0A8C0PD54	-	M-phase phosphoprotein 8	1.845393333	3.593509085	Up	6.488E-07
A0A8I3MVW3	CNOT8	poly(A)-specific ribonuclease	1.847343333	3.598369489	Up	1.78E-06
A0A8I3RZ04	CENPH	Centromere protein H	1.856213333	3.620561189	Up	1.141E-07
A0A8I3RX25	TAF9	TATA-box binding protein associated factor 9	1.862133333	3.635448437	Up	1.099E-06
A0A8C0MI74	COMMD5	COMM domain-containing protein 5	1.862343333	3.635977655	Up	3.412E-05
A0A8C0QG76	-	TATA-box binding protein associated factor 3	1.863596667	3.639137763	Up	4.229E-07
A0A8C0RN30	SLC25A15	Solute carrier family 25 member 15	1.86463	3.641745236	Up	1.335E-06
A0A8I3PBY2	TMEM115	Transmembrane protein 115	1.865516667	3.643984106	Up	9.739E-06
A0A8I3QAH4	CHEK2	Checkpoint kinase 2	1.86683	3.647302857	Up	3.244E-06

A0A8I3MF34	NOP53	Ribosome biogenesis protein NOP53	1.86764	3.649351207	Up	1.404E-07
A0A8C0TKI6	ZNF740	Zinc finger protein 740	1.867836667	3.649848717	Up	7.178E-05
A0A8I3NUR1	TSSC4	Protein TSSC4	1.873673333	3.664644706	Up	1.915E-05
A0A8P0SKL6	WIZ	WIZ zinc finger	1.87499	3.667990748	Up	4.768E-08
A0A8C0QF48	PAFAH1B3	Platelet activating factor acetylhydrolase 1b catalytic subunit 3	1.88251	3.687159944	Up	1.44E-05
A0A8I3NP89	CCZ1	Vacuolar fusion protein CCZ1 homolog	1.88454	3.692351757	Up	4.996E-06
A0A8I3NF66	MRPS9	Mitochondrial ribosomal protein S9	1.88695	3.698524929	Up	4.261E-06
A0A8C0RZT3	-	non-specific serine/threonine protein kinase	1.891783333	3.710936549	Up	5.73E-06
V5LJQ6	ND2	NADH-ubiquinone oxidoreductase chain 2	1.892236667	3.712102807	Up	2.408E-05
A0A8C0Q6S1	-	Essential for reactive oxygen species protein	1.89772	3.726238454	Up	6.754E-06
A0A8C0N7T2	IFT20	Intraflagellar transport 20	1.90138	3.735703619	Up	7.336E-07
A0A8C0RQF1	KIF1C	Kinesin family member 1C	1.904086667	3.74271882	Up	3.838E-08
A0A8I3SBX9	RFT1	Protein RFT1 homolog	1.90583	3.747244205	Up	2.187E-07
A0A8C0TE16	KIAA0100	KIAA0100	1.910953333	3.760575165	Up	6.547E-06
A0A8I3P5K0	ZDHHC20	Palmitoyltransferase	1.91188	3.76299142	Up	6.587E-06
A0A8C0RL30	PRPF18	Pre-mRNA-splicing factor 18	1.912173333	3.763756601	Up	4.667E-06
A0A8C0RHA4	-	N-acetylglucosamine-1-phosphate transferase subunits alpha and beta	1.91654	3.775165782	Up	2.576E-06
A0A8C0PKZ3	ACTR8	Actin-related protein 8	1.924713333	3.796614013	Up	5.311E-07
A0A8C0PWH7	-	PBX homeobox interacting protein 1	1.931756667	3.815194655	Up	2.648E-06
A0A8I3MUC7	MICU1	Mitochondrial calcium uptake 1	1.93201	3.815864652	Up	3.417E-06
A0A8C0MB25	-	Protein phosphatase 4 regulatory subunit 1	1.93361	3.820098929	Up	4.367E-07
A0A8C0SBU6	CCNT2	Cyclin T2	1.934733333	3.823074551	Up	6.216E-06
A0A8I3NL82	LANCL2	LanC like 2	1.93588	3.826114372	Up	1.068E-06
A0A8I3PN63	SLC43A3	Solute carrier family 43 member 3	1.937126667	3.829422036	Up	1.803E-06
A0A8C0P082	-	Methyltransferase like 15	1.93755	3.830545877	Up	0.0001229
A0A8I3MQ14	RSPRY1	Ring finger and SPRY domain containing 1	1.938	3.831740873	Up	7.436E-06
A0A8C0RWR0	SMARCA2	SWI/SNF related, matrix associated, actin dependent	1.93897	3.834318021	Up	2.72E-06

		regulator of chromatin, subfamily a, member 2				
A0A8I3PDY8	GID8	GID complex subunit 8 homolog	1.941756667	3.841731432	Up	4.54E-08
A0A8I3N9W6	SCAP	Sterol regulatory element-binding protein cleavage-activating protein	1.941936667	3.842210782	Up	2.787E-05
A0A8C0RYD8	-	Metabolism of cobalamin associated A	1.944316667	3.848554471	Up	3.251E-07
A0A8P0P474	LOC476436	DRY_EERY domain-containing protein	1.94577	3.852433357	Up	1.092E-06
A0A8I3PD14	CAB39	Calcium binding protein 39	1.94683	3.855264919	Up	6.844E-06
A0A8C0SRT2	FIGNL1	Fidgetin like 1	1.955733333	3.87913056	Up	0.0003346
A0A8C0QDR6	-	GATOR complex protein WDR24	1.955846667	3.879435303	Up	0.0002239
A0A8P0NGY5	TULP3	Tubby-like protein	1.956706667	3.881748549	Up	4.342E-06
A0A8I3RZV6	TANC2	Tetratricopeptide repeat, ankyrin repeat and coiled-coil containing 2	1.960843333	3.892894732	Up	1.662E-07
A0A8I3NKF5	MELK	Maternal embryonic leucine zipper kinase	1.960883333	3.893002668	Up	4.372E-07
A0A8C0THG6	-	DGCR8 microprocessor complex subunit	1.962823333	3.898241131	Up	4.706E-07
A0A8C0PIX9	-	Adaptor related protein complex 4 subunit epsilon 1	1.97032	3.918550256	Up	1.744E-06
Q6EIY9	KRT1	Keratin, type II cytoskeletal 1	1.97055	3.919175016	Up	0.014856
A0A8C0Z6R3	-	Histone H2B	1.97087	3.920044414	Up	6.536E-06
A0A8C0TG11	-	Mitochondrial transcription termination factor 3	1.974693333	3.930446829	Up	3.434E-06
A0A8I3MSC5	FANCI	FA complementation group I	1.977056667	3.93689072	Up	4.514E-08
A0A8I3P6E4	WDR35	WD repeat-containing protein 35	1.977443333	3.937946014	Up	1.866E-07
A0A8I3S3E3	KYAT3	Kynurenine--oxoglutarate transaminase 3	1.97813	3.939820769	Up	1.433E-08
A0A8I3N9L2	GPATCH4	G-patch domain-containing protein	1.979323333	3.943080963	Up	6.01E-08
A0A8C0P731	RB1CC1	RB1 inducible coiled-coil 1	1.98075	3.946982164	Up	8.598E-06
A0A8C0MNG5	EZH2	Histone-lysine N-methyltransferase EZH2	1.98182	3.949910599	Up	8.722E-07
A0A8I3P8R4	NDUFAF5	NADH:ubiquinone oxidoreductase complex assembly factor 5	1.98368	3.95500632	Up	9.692E-06
A0A8C0S9G5	GOLPH3L	Golgi phosphoprotein 3 like	1.983853333	3.955481525	Up	1.096E-05
A0A8C0YW02	CXXC1	CXXC-type zinc finger protein 1	1.987986667	3.966830261	Up	3.004E-06
A0A8I3Q2V6	MINPP1	Multiple inositol polyphosphate phosphatase 1	1.988486667	3.968205298	Up	2.629E-07
A0A8I3R	MLX	BHLH domain-containing	1.99714	3.99207	Up	7.842

UP9		protein		8251		E-07
A0A8P0NR77	D2HGDH	D-2-hydroxyglutarate dehydrogenase	1.99998	3.999944549	Up	2.489E-07
A0A8C0SMR3	DNAJB9	DnaJ heat shock protein family (Hsp40) member B9	2.002646667	4.007344853	Up	9.854E-05
A0A8C0Z3P0	-	Golgin B1	2.00389	4.010799924	Up	2.231E-05
A0A8I3MT60	HEATR3	HEAT repeat containing 3	2.004123333	4.01144866	Up	7.863E-07
A0A8I3PY30	MARS2	Methionine--tRNA ligase, mitochondrial	2.004766667	4.013237863	Up	2.79E-06
A0A8I3QYU2	NIPSNAP1	Nipsnap homolog 1	2.00798	4.022186562	Up	8.316E-07
A0A8C0N7X5	SUGP1	SURP and G-patch domain containing 1	2.01284	4.035758931	Up	1.496E-06
A0A8C0SID5	-	Helicase SRCAP	2.022543333	4.06299428	Up	1.186E-06
A0A8C0T1P3	MED17	Mediator of RNA polymerase II transcription subunit 17	2.024836667	4.069458023	Up	3.569E-07
A0A8I3MDI0	ANGEL2	Angel homolog 2	2.02611	4.073051342	Up	1.262E-06
A0A8C0NG70	NR2C1	FYVE, RhoGEF and PH domain containing 6	2.039186667	4.110137523	Up	8.688E-06
A0A8C0YZ44	RAB2B	RAB2B, member RAS oncogene family	2.048553333	4.136909317	Up	2.106E-07
A0A8C0U0J1	LOC102156482	RNA-binding protein Musashi homolog 2	2.051603333	4.145664404	Up	7.926E-07
A0A8C0P4D7	TADA1	Transcriptional adapter 1	2.053173333	4.150178342	Up	3.313E-07
A0A8I3PRK1	SKA2	Spindle and kinetochore-associated protein 2	2.05671	4.160364696	Up	1.066E-05
Q9XT74	SLC11A1	Natural resistance-associated macrophage protein 1	2.06302	4.178600979	Up	2.866E-05
A0A8I3MYV2	SPEN	Spen family transcriptional repressor	2.069216667	4.196587514	Up	1.328E-06
A0A8C0YTJ9	-	Cytoplasmic linker associated protein 2	2.0709	4.201486941	Up	7.508E-07
A0A8C0T6T8	VTN	Vitronectin	2.072226667	4.205352301	Up	4.291E-05
A0A8P0SL80	TBC1D10B	Rab-GAP TBC domain-containing protein	2.07336	4.208657185	Up	1.427E-05
A0A8C0RE79	-	Serine/threonine-protein kinase	2.078193333	4.222780721	Up	1.002E-05
A0A8I3QTE9	ALS2	Alsin Rho guanine nucleotide exchange factor ALS2	2.084773333	4.242084425	Up	2.285E-05
A0A8C0YZQ4	MGAT4A	Cytochrome c oxidase assembly factor 5	2.089253333	4.255277841	Up	7.496E-06
A0A8I3S426	POLR1H	DNA-directed RNA polymerase I subunit RPA12	2.09063	4.259340304	Up	1.177E-05
A0A8C0RBH9	NSD1	Nuclear receptor binding SET domain protein 1	2.09093	4.260226101	Up	2.001E-06
A0A8C0NI81	-	Golgi associated PDZ and coiled-coil motif containing	2.100413333	4.288322282	Up	4.763E-06

A0A8C0SC44	SLC12A9	Solute carrier family 12 member 9	2.10237	4.294142299	Up	3.891E-06
A0A8I3PES9	SMARCD2	SWI/SNF related, matrix associated, actin dependent regulator of chromatin, subfamily d, member 2	2.104303333	4.29990067	Up	8.073E-07
A0A8C0P1X5	-	Glutathione S-transferase	2.10635	4.306005016	Up	3.067E-06
A0A8I3NXJ8	YEATS4	YEATS domain containing 4	2.107886667	4.310593941	Up	1.193E-06
A0A8I3PX52	TEX30	Testis expressed 30	2.110173333	4.317431635	Up	6.667E-06
A0A8I3PIK8	AHSG	Alpha-2-HS-glycoprotein	2.113773333	4.328218504	Up	3.428E-05
A0A8C0SF96	DCP2	Decapping mRNA 2	2.120676667	4.348978772	Up	5.761E-06
A0A8I3Q0D6	SUDS3	SDS3 homolog, SIN3A corepressor complex component	2.123933333	4.358807025	Up	7.814E-06
A0A8I3NT37	RAD23A	UV excision repair protein RAD23	2.124546667	4.360660479	Up	1.503E-05
A0A8I3MY92	MTX3	Metaxin 3	2.127906667	4.370828182	Up	3.577E-06
A0A8P0NG60	GNB1L	G protein subunit beta 1 like	2.129456667	4.375526628	Up	4.94E-06
A0A8I3S4D1	APOB	Apolipoprotein B	2.13009	4.377447876	Up	8.885E-06
A0A8I3NAI9	C8orf82	Chromosome 8 open reading frame 82	2.130303333	4.378095223	Up	7.283E-06
A0A8I3QD03	RNF220	RING-type domain-containing protein	2.1311	4.380513507	Up	4.806E-06
A0A8C0SNG6	-	Sec1 family domain containing 2	2.134256667	4.390108715	Up	9.363E-06
A0A8C0QN81	NCK2	NCK adaptor protein 2	2.13632	4.396391913	Up	7.942E-07
A0A8C0MDV0	-	Eukaryotic translation initiation factor 2 alpha kinase 3	2.13695	4.398312161	Up	3.057E-07
A0A8C0ME09	UBN2	Ubinuclein 2	2.137103333	4.398779649	Up	2.159E-06
A0A8I3P1R2	GEN1	GEN1 Holliday junction 5' flap endonuclease	2.139163333	4.405065079	Up	3.093E-06
A0A8I3PT05	TMCC1	Transmembrane and coiled-coil domain family 1	2.139516667	4.406144065	Up	2.584E-05
A0A8P0PD31	NXT1	NTF2-related export protein	2.141273333	4.411512379	Up	4.394E-06
A0A8C0Q593	-	Formin binding protein 1	2.141833333	4.413225095	Up	4.385E-06
A0A8P0SCW0	VAMP5	Vesicle associated membrane protein 5	2.143843333	4.419377999	Up	2.018E-06
A0A8I3PJR4	CKAP2	Cytoskeleton associated protein 2	2.149076667	4.435438273	Up	2.106E-06
A0A8C0S8Q1	DSCC1	Sister chromatid cohesion protein DCC1	2.155303333	4.45462298	Up	1.09E-05
A0A8C0Q8E6	GRPEL2	GrpE protein homolog	2.158063333	4.463153214	Up	3.434E-06
A0A8C0	NDST1	[heparan sulfate]-	2.1586266	4.46489	Up	1.124

QI33		glucosamine N-sulfotransferase	67	6295		E-06
A0A8I3NCG1	TONSL	Tonsoku-like protein	2.1587	4.465123255	Up	1.555E-05
A0A8I3S162	NOD1	Nucleotide binding oligomerization domain containing 1	2.15964	4.468033491	Up	2.955E-06
A0A8I3Q8I9	TNK2	non-specific protein-tyrosine kinase	2.159926667	4.468921388	Up	4.96E-06
A0A8C0M615	CENPL	Centromere protein L	2.160503333	4.470708039	Up	8.215E-06
A0A8C0M344	-	OMA1 zinc metallopeptidase	2.164203333	4.482188532	Up	4.389E-06
A0A8C0Q8A3	LLGL1	LLGL scribble cell polarity complex component 1	2.16513	4.48506844	Up	1.533E-07
A0A8C0QF78	NIPBL	Nipped-B protein	2.1659	4.487462864	Up	5.842E-07
A0A8I3PUI7	PRKDC	DNA-dependent protein kinase catalytic subunit	2.166323333	4.488779824	Up	5.068E-08
A0A8I3S0W9	NUBP2	Cytosolic Fe-S cluster assembly factor NUBP2	2.167836667	4.493490857	Up	6.167E-06
A0A8I3MHU4	AJUBA	Ajuba LIM protein	2.1695	4.498674547	Up	1.009E-06
A0A8C0N1T6	DOCK9	Dedicator of cytokinesis 9	2.181346667	4.535767424	Up	7.2E-07
A0A8C0S682	GATB	Glutamyl-tRNA(Gln) amidotransferase subunit B, mitochondrial	2.18273	4.540118647	Up	3.504E-06
A0A8C0Q899	-	Protein-arginine deiminase	2.187796667	4.556091328	Up	1.214E-06
A0A8C0Q349	SNX11	Sorting nexin 11	2.189633333	4.561895292	Up	6.421E-07
A0A8I3MU19	DNLZ	DNL-type zinc finger	2.19297	4.572458259	Up	3.807E-07
A0A8C0NXL4	-	Ribonuclease H2 subunit B	2.19313	4.572965389	Up	1.025E-06
A0A8I3PTK7	STK3	non-specific serine/threonine protein kinase	2.20096	4.597851911	Up	6.375E-07
A0A8I3MKP5	SERPINB5	Serpin B5	2.22443	4.673262252	Up	1.071E-05
A0A8C0M077	ADGRG1	Adhesion G-protein coupled receptor G1	2.235023333	4.707703088	Up	3.296E-06
A0A8I3N4A1	CDT1	Chromatin licensing and DNA replication factor 1	2.240033333	4.724079794	Up	1.002E-05
A0A8I3RRH9	FBXW11	F-box and WD repeat domain containing 11	2.244316667	4.738126336	Up	1.888E-06
A0A8C0TEP3	INTS8	Integrator complex subunit 8	2.246513333	4.745346165	Up	5.145E-08
A0A8I3N4K8	CDC26	Anaphase-promoting complex subunit CDC26	2.250676667	4.759060077	Up	9.515E-08
A0A8P0S743	C14H1orf35	Chromosome 14 C1orf35 homolog	2.250886667	4.75975286	Up	1.197E-05
A0A8C0MWZ1	ATG2B	Autophagy related 2B	2.25723	4.780726921	Up	3.394E-08
A0A8I3Q5Y5	KIF4A	Kinesin family member 4A	2.257643333	4.782096799	Up	3.486E-08

A0A8C0S9Q8	PRAF2	WD repeat domain phosphoinositide-interacting protein 4	2.27406	4.836823857	Up	3.115E-06
A0A8I3RR94	COG2	Conserved oligomeric Golgi complex subunit 2	2.278883333	4.853021775	Up	5.171E-06
A0A8I3MP86	TAF5L	TATA-box binding protein associated factor 5 like	2.28237	4.864764612	Up	5.47E-07
A0A8C0QFH2	EPN1	Epsin 1	2.286516667	4.878767277	Up	4.151E-06
A0A8C0NL66	C6H16orf91	Chromosome 6 C16orf91 homolog	2.291876667	4.896926922	Up	2.157E-06
A0A8I3NTN0	TOMM34	Translocase of outer mitochondrial membrane 34	2.297943333	4.917562312	Up	8.604E-07
A0A8C0M8G0	-	Annexin	2.30086	4.927514102	Up	3.592E-06
A0A8I3MZ72	RAPGEF6	Rap guanine nucleotide exchange factor 6	2.303563333	4.936755972	Up	1.808E-06
A0A8C0RGS0	-	RNA binding motif protein 15B	2.305666667	4.943958615	Up	4.624E-07
A0A8C0M6Q6	TMEM63A	Transmembrane protein 63A	2.306116667	4.945500956	Up	3.875E-05
A0A8C0TS74	CFAP58	Cilia and flagella associated protein 58	2.30891	4.955085667	Up	1.665E-05
A0A8I3Q1Y3	SLC35F6	Solute carrier family 35 member F6	2.312036667	4.965836173	Up	4.587E-06
A0A8C0P1U5	KIF18A	Kinesin-like protein	2.312513333	4.967477157	Up	1.518E-05
A0A8P0TGC8	RASA1	RAS p21 protein activator 1	2.312896667	4.968797223	Up	5.583E-06
A0A8I3N4L7	PRR3	Proline-rich protein 3	2.32214	5.000734461	Up	2.345E-06
A0A8C0M5D9	POLR3B	DNA-directed RNA polymerase subunit beta	2.33002	5.028123202	Up	1.794E-07
A0A8C0T2S0	SIRT5	NAD-dependent protein deacylase sirtuin-5, mitochondrial	2.33295	5.038345301	Up	1.318E-07
A0A8C0U0I0	-	CUE domain containing 1	2.340003333	5.063038074	Up	1.184E-06
A0A8C0NR41	ATM	Serine-protein kinase ATM	2.34369	5.07599272	Up	2.056E-07
A0A8I3N133	CCDC51	Coiled-coil domain containing 51	2.352343333	5.106530185	Up	5.855E-06
A0A8P0T6F5	RIC8A	Synembryn	2.358143333	5.127101054	Up	5.405E-06
A0A8C0N447	COG8	Conserved oligomeric Golgi complex subunit 8	2.360736667	5.136325623	Up	6.658E-07
A0A8I3RZA9	OTUD7B	ubiquitinyl hydrolase 1	2.361413333	5.138735277	Up	6.439E-07
A0A8I3PDL5	VPS72	Vacuolar protein sorting-associated protein 72 homolog	2.363686667	5.146839046	Up	2.656E-06
A0A8C0PPD0	TTC4	Tetratricopeptide repeat domain 4	2.367396667	5.160091566	Up	6.518E-06
A0A8C0SIB6	NDEL1	Nuclear distribution protein nudE-like 1	2.371376667	5.174346498	Up	0.0001837
A0A8C0NRW6	-	RNA helicase	2.371493333	5.174764949	Up	1.548E-07

A0A8C0NIZ7	MSANTD2	Myb/SANT DNA binding domain containing 2	2.37284	5.179597528	Up	5.353E-07
A0A8C0M3M6	KIFC3	Kinesin family member C3	2.374603333	5.185932159	Up	6.036E-06
A0A8I3NGE8	H2BC27	Histone H2B	2.375086667	5.187669847	Up	8.322E-08
A0A8C0MMU8	-	G2 and S-phase expressed 1	2.37716	5.195130537	Up	2.734E-06
A0A8C0TNT3	SMS	Spermine synthase	2.385433333	5.225008316	Up	1.102E-06
A0A8C0MG33	-	Cathepsin H	2.38688	5.230250336	Up	5.374E-07
A0A8C0NEB9	POLR3C	DNA-directed RNA polymerase III subunit RPC3	2.39675	5.266155054	Up	2.802E-09
A0A8I3P7G3	C5H17orf49	Chromosome 17 open reading frame 49	2.40166	5.28410817	Up	1.981E-07
A0A8P0P943	FAM193A	Family with sequence similarity 193 member A	2.408666667	5.30983366	Up	1.241E-07
A0A8P0SJH9	SUCO	SUN domain containing ossification factor	2.41091	5.318096662	Up	1.131E-05
A0A8I3PDU7	TACO1	Translational activator of cytochrome c oxidase I	2.421566667	5.357524962	Up	3.694E-06
A0A8C0T9D6	-	AT-rich interaction domain 2	2.421646667	5.357822054	Up	4.819E-07
A0A8C0Z0N4	SLC6A6	Transporter	2.42617	5.374646987	Up	3.271E-05
A0A8C0NJV3	-	60S ribosomal protein L21	2.433073333	5.400426441	Up	6.71E-06
A0A8C0QPE5	-	tRNA (adenine(58)-N(1))-methyltransferase non-catalytic subunit TRM6	2.43518	5.408318067	Up	1.201E-06
A0A8I3P736	MAPKAPK3	non-specific serine/threonine protein kinase	2.437916667	5.418586911	Up	4.333E-06
A0A8I3N9X1	KNOP1	Lysine rich nucleolar protein 1	2.43964	5.425063409	Up	7.128E-07
A0A8C0M3Z6	-	Phosphatidylinositol 4-kinase type 2	2.443243333	5.438630201	Up	1.086E-05
A0A8C0MAF2	NCOR1	Nuclear receptor corepressor 1	2.445456667	5.446980365	Up	3.535E-07
A0A8I3PKH5	MTMR1	Phosphatidylinositol-3,5-bisphosphate 3-phosphatase	2.446326667	5.450266092	Up	6.513E-07
A0A8C0NTS1	C4H1orf131	Chromosome 4 C1orf131 homolog	2.452586667	5.473966732	Up	3.84E-06
A0A8C0NG89	SUPT20H	SPT20 homolog, SAGA complex component	2.456583333	5.489152167	Up	3.177E-05
A0A8C0QQ09	TRMU	Mitochondrial tRNA-specific 2-thiouridylase 1	2.464093333	5.517800643	Up	7.038E-06
A0A8I3Q2Y1	CHAMP1	Chromosome alignment maintaining phosphoprotein 1	2.464146667	5.518004628	Up	9.47E-07
A0A8C0N2D2	METTL26	Methyltransferase like 26	2.466676667	5.527689835	Up	4.186E-08
A0A8P0SHY1	SIRT1	Sirtuin 1	2.47849	5.573138474	Up	4.532E-08
A0A8C0L	SLC26A2	Sulfate transporter	2.4906	5.62011	Up	2.058

YQ3				6358		E-05
A0A8C0T9K7	TRPM7	non-specific serine/threonine protein kinase	2.496933333	5.64484252	Up	6.966E-07
A0A8C0NP38	-	G protein-coupled receptor 155	2.499023333	5.653026004	Up	4.726E-06
J7MDG3	CDKAL1	tRNA-t(6)A37 methylthiotransferase	2.500176667	5.657547008	Up	7.431E-07
A0A8I3MYB5	PNISR	PNN interacting serine and arginine rich protein	2.509596667	5.694608522	Up	2.386E-07
A0A8C0NYP5	-	Prickle planar cell polarity protein 1	2.518156667	5.728497006	Up	1.872E-06
A0A8C0LU82	-	histone acetyltransferase	2.52261	5.746207139	Up	5.558E-07
A0A8I3PMJ4	DIS3L2	DIS3-like exonuclease 2	2.52538	5.757250557	Up	1.828E-06
A0A8C0RZM2	-	Cyclin D1	2.528306667	5.768941631	Up	5.08E-07
A0A8C0RXB1	LTV1	Protein LTV1 homolog	2.533413333	5.789397973	Up	8.14E-07
A0A8I3NX48	ORC6	Origin recognition complex subunit 6	2.53356	5.789986563	Up	2.531E-07
A0A8C0M9N3	-	Cytochrome P450 family 27 subfamily C member 1	2.53492	5.795447241	Up	5.546E-06
A0A8I3RUW6	NDE1	NudE neurodevelopment protein 1	2.535543333	5.797951774	Up	2.245E-07
A0A8I3N491	PIAS4	Protein inhibitor of activated STAT 4	2.548216667	5.849108158	Up	1.831E-09
A0A8I3PQ14	MTRR	5-methyltetrahydrofolate-homocysteine methyltransferase reductase	2.556506667	5.882814996	Up	3.269E-07
A0A8C0N5D8	SLC9A1	Sodium/hydrogen exchanger	2.558876667	5.892486984	Up	7.055E-06
A0A8I3P056	MRPL47	Mitochondrial ribosomal protein L47	2.560616667	5.899598059	Up	7.298E-06
A0A8P0TU12	EBAG9	Receptor-binding cancer antigen expressed on SiSo cells	2.57095	5.942005745	Up	5.14E-07
A0A8I3Q673	PDZD8	PDZ domain containing 8	2.579323333	5.976593138	Up	1.588E-07
A0A8C0N699	VPS13B	Vacuolar protein sorting 13 homolog B	2.582246667	5.988715788	Up	5.514E-08
A0A8C0NIZ1	TXLNG	Taxilin gamma	2.625526667	6.171095695	Up	2.523E-07
A0A8I3S7X1	OCRL	phosphoinositide 5-phosphatase	2.627056667	6.177643708	Up	5.752E-07
A0A8C0SIV0	EXD2	Exonuclease 3'-5' domain containing 2	2.62712	6.177914908	Up	1.431E-06
A0A8I3S4P9	GPS2	G protein pathway suppressor 2	2.64829	6.269237565	Up	7.737E-07
A0A8C0TCF4	-	TATA-box binding protein associated factor 4	2.64916	6.273019294	Up	1.571E-05
A0A8I3N6U4	MDC1	Mediator of DNA damage checkpoint protein 1	2.650813333	6.280212316	Up	3.881E-06
A0A8C0SJK6	CTTNBP2NL	CTTNBP2 N-terminal like	2.655143333	6.299089603	Up	2.3E-06
A0A8C0S	CNOT10	CCR4-NOT transcription	2.6565966	6.30543	Up	1.957

CZ2		complex subunit 10	67	8339		E-07
A0A8I3MY05	TUBB	Tubulin beta chain	2.656833333	6.306472798	Up	1.401E-06
A0A8C0T526	-	ArfGAP with RhoGAP domain, ankyrin repeat and PH domain 1	2.658856667	6.315323628	Up	2.024E-06
A0A8C0NJA9	MSL1	MSL complex subunit 1	2.66171	6.327826308	Up	1.247E-06
A0A8P0PB33	PDLIM2	PDZ and LIM domain 2	2.665383333	6.343958499	Up	2.425E-06
A0A8I3MXD6	LOC479708	tRNA 4-demethylwyosine synthase (AdoMet-dependent)	2.668586667	6.358060157	Up	2.003E-06
A0A8C0QLM8	CUTA	CutA divalent cation tolerance homolog	2.668983333	6.359808536	Up	2.137E-06
A0A8C0QDL4	SELENOF	Sep15_SelM domain-containing protein	2.673253333	6.378659789	Up	2.416E-06
A0A8I3NE51	DEGS1	Sphingolipid delta(4)-desaturase DES1	2.677283333	6.39650274	Up	2.879E-06
A0A8I3RRB2	PLEKHF1	Pleckstrin homology and FYVE domain containing 1	2.682566667	6.419970494	Up	2.867E-05
A0A8I3PJM3	-	AP2 domain transcription factor AP2X-11	2.70472	6.51931326	Up	6.701E-07
A0A8C0T108	PIH1D1	PIH1 domain containing 1	2.704826667	6.519795288	Up	2.979E-06
A0A8C0RAS3	RABGAP1L	RAB GTPase activating protein 1 like	2.722156667	6.59858491	Up	1.399E-06
A0A8P0NQ50	AURKA	Aurora kinase	2.722686667	6.601009465	Up	5.508E-09
A0A8C0M966	-	Chromobox 4	2.722813333	6.60158905	Up	5.194E-07
A0A8I3NUU3	SCRN2	Secernin 2	2.727986667	6.62530406	Up	2.893E-06
A0A8I3S1K9	HIP1R	Huntingtin interacting protein 1 related	2.728493333	6.627631239	Up	1.737E-07
A0A8C0MT88	ACVR1	Serine/threonine-protein kinase receptor	2.72853	6.627799685	Up	7.596E-06
A0A8I3MIB6	ARHGAP18	Rho GTPase activating protein 18	2.736283333	6.663514698	Up	3.099E-07
A0A8I3PQA8	NOCT	Nocturnin	2.7491	6.722976003	Up	9.347E-07
A0A8C0PUJ6	DLGAP5	DLG associated protein 5	2.751456667	6.733967072	Up	1.309E-06
A0A8C0T477	CNNM4	Cyclin and CBS domain divalent metal cation transport mediator 4	2.752216667	6.737515406	Up	2.431E-07
A0A8C0PUV7	-	HAUS augmin like complex subunit 5	2.768736667	6.815108694	Up	9.74E-07
A0A8I3PJ60	SETDB1	SET domain bifurcated histone lysine methyltransferase 1	2.77883	6.862955499	Up	9.193E-07
A0A8I3PFH3	ARRDC1	Arrestin domain containing 1	2.781676667	6.87651057	Up	9.858E-07
A0A8C0TZ26	SPINT1	Serine peptidase inhibitor, Kunitz type 1	2.78698	6.901835076	Up	8.586E-07
A0A8I3PP46	MON1A	Vacuolar fusion protein MON1 homolog	2.797786667	6.953728147	Up	4.23E-05

A0A8I3S0Y2	XPNPEP3	X-prolyl aminopeptidase 3	2.80771	7.001723061	Up	1.533E-06
A0A8I3PI45	SREBF1	Sterol regulatory element binding transcription factor 1	2.80871	7.006577968	Up	2.641E-08
A0A8I3MLN4	RNF214	Ring finger protein 214	2.808793333	7.006982695	Up	6.793E-07
A0A8I3NDT1	ECD	Ecdysoneless cell cycle regulator	2.81099	7.017659747	Up	4.514E-06
A0A8C0MNM9	-	tRNA (guanine(10)-N2)-methyltransferase homolog	2.81138	7.019557069	Up	1.272E-06
A0A8P0SG41	TM7SF3	Transmembrane 7 superfamily member 3	2.818426667	7.053927103	Up	2.13E-06
A0A8C0SJF5	TEFM	Transcription elongation factor, mitochondrial	2.82685	7.095232697	Up	2.512E-07
A0A8C0QBB8	ATR	Serine/threonine-protein kinase ATR	2.82802	7.100989138	Up	7.091E-07
A0A8C0MHX1	UBE2R2	Ubiquitin conjugating enzyme E2 R2	2.831	7.115671948	Up	2.87E-06
A0A8C0M3Y7	DCTPP1	dCTP pyrophosphatase 1	2.839176667	7.15611547	Up	2.225E-06
A0A8C0TC95	-	Melanoregulin	2.856956667	7.244854252	Up	2.629E-06
A0A8I3PJC8	SUSD2	Sushi domain containing 2	2.864106667	7.280848887	Up	7.827E-07
A0A8C0Z1J4	-	CP-type G domain-containing protein	2.875546667	7.338812645	Up	2.603E-08
A0A8I3PIP6	SENP8	SUMO peptidase family member, NEDD8 specific	2.878213333	7.352390196	Up	6.724E-05
A0A8C0SBS7	MTBP	MDM2 binding protein	2.88374	7.380609701	Up	3.068E-05
A0A8I3NYG9	NELFA	Negative elongation factor complex member A	2.896863333	7.44805297	Up	7.914E-07
A0A8C0Q8D4	TKFC	Triokinase/FMN cyclase	2.90042	7.466437259	Up	1.944E-05
A0A8C0PVY5	-	RAD51 paralog C	2.923923333	7.589071217	Up	3.323E-07
A0A8C0MZ41	TENT4B	Terminal nucleotidyltransferase 4B	2.925206667	7.595824995	Up	1.392E-06
A0A8I3PA98	NUDT16L1	Nudix hydrolase 16 like 1	2.936326667	7.654598285	Up	3.54E-07
A0A8I3RXS7	DPH1	2-(3-amino-3-carboxypropyl)histidine synthase subunit 1	2.942743333	7.688719422	Up	8.694E-07
A0A8C0Z0A4	-	Frizzled class receptor 1	2.953663333	7.747137434	Up	1.626E-07
A0A8C0PAF4	-	Protein SPT2 homolog	2.9583	7.772075954	Up	2.918E-07
A0A8C0YRB2	ATXN10	Ataxin-10	2.96476	7.80695525	Up	1.263E-06
A0A8C0NHR5	MACO1	Macoilin	2.968436667	7.826876423	Up	3.742E-07
A0A8C0Q4S5	ARID4B	AT-rich interaction domain 4B	2.970916667	7.840342434	Up	2.575E-07
A0A8I3RZK6	MGME1	Mitochondrial genome maintenance exonuclease 1	2.98554	7.92021723	Up	4.006E-06
A0A8C0S	-	Chromosome 11 open	2.9868866	7.92761	Up	4.336

Z26		reading frame 54	67	3715		E-06
A0A8C0PYE5	-	Probable glutamate--tRNA ligase, mitochondrial	2.993576667	7.964460652	Up	2.185E-06
A0A8C0Q6I9	-	Pro-apoptotic WT1 regulator	3.013176667	8.073401647	Up	3.676E-06
A0A8P0SM50	INF2	Inverted formin-2	3.01403	8.07817836	Up	6.29E-07
A0A8P0T0P2	LARS2	leucine--tRNA ligase	3.014546667	8.081071884	Up	3.191E-07
A0A8P0T631	CALU	Calumenin	3.01688	8.094152328	Up	4.701E-07
A0A8C0ND40	ERCC6L	ERCC excision repair 6 like, spindle assembly checkpoint helicase	3.03087	8.173024164	Up	7.352E-07
A0A8I3MG00	MED7	Mediator of RNA polymerase II transcription subunit 7	3.03169	8.177670874	Up	2.007E-05
A0A8I3P5D6	PCBP2	Poly(RC)-binding protein 2	3.032926667	8.184683713	Up	2.861E-05
A0A172R7M8	ND1	NADH-ubiquinone oxidoreductase chain 1	3.038996667	8.219192525	Up	2.873E-07
A0A8C0MA79	-	TSPY like 1	3.048096667	8.271200077	Up	7.274E-06
A0A8C0MXT1	KIF3A	Kinesin-like protein	3.063003333	8.357105412	Up	4.216E-06
A0A8C0T4P1	ICE2	Interactor of little elongation complex ELL subunit 2	3.071773333	8.40806215	Up	6.133E-06
A0A8I3NSD8	VPS50	VPS50 subunit of EARP/GARPII complex	3.093083333	8.533179128	Up	3.511E-07
A0A8C0S633	-	Coiled-coil domain containing 90B	3.096916667	8.555882482	Up	6.169E-07
A0A8C0MSI1	FAM91A1	Family with sequence similarity 91 member A1	3.09928	8.569909683	Up	7.932E-08
A0A8I3RQN0	POLE2	DNA polymerase epsilon subunit	3.11649	8.672752867	Up	5.393E-07
P63091	GNAS	Guanine nucleotide-binding protein G(s) subunit alpha	3.136593333	8.794449867	Up	1.087E-06
A0A8I3N7R6	TADA3	Transcriptional adapter 3	3.138356667	8.805205451	Up	2.459E-06
A0A8C0MA29	-	LLGL scribble cell polarity complex component 2	3.166686667	8.979820872	Up	1.303E-06
A0A8I3PTN7	SUFU	Suppressor of fused homolog	3.195423333	9.160480871	Up	5.401E-07
A0A8I3PKM8	NFX1	Nuclear transcription factor, X-box binding 1	3.205943333	9.227522392	Up	1.225E-07
A0A8I3NKZ0	SPPL2B	Signal peptide peptidase like 2B	3.213903333	9.278575511	Up	2.366E-06
A0A8C0MAM6	ZNF451	Zinc finger protein 451	3.22618	9.357868788	Up	3.33E-07
A0A8P0NRL7	SIKE1	Suppressor of IKBKE 1	3.23443	9.411534723	Up	2.503E-07
A0A8I3PLS2	DYNLT2	PHD finger protein 10	3.26484	9.61202233	Up	3.439E-06
A0A8I3NQG7	PITPNA	Phosphatidylinositol transfer protein alpha	3.288903333	9.773689913	Up	9.267E-07
A0A8I3P	MTFR1	Mitochondrial fission	3.3018633	9.86188	Up	1.782

291		regulator	33	4342		E-07
A0A8C0NXI2	SAP30	Sin3A associated protein 30	3.301876667	9.861975486	Up	2.207E-07
A0A8C0MY13	SATB2	DNA-binding protein SATB	3.31088	9.923712916	Up	6.625E-07
A0A8I3N9A9	FRYL	FRY like transcription coactivator	3.313796667	9.943795775	Up	1.844E-08
A0A8I3MHY5	LOC102152446	Coronin	3.31807	9.97329345	Up	1.376E-07
A0A8C0PA05	SENP1	SUMO specific peptidase 1	3.318876667	9.978871464	Up	8.141E-07
A0A8C0P458	PLXND1	Plexin D1	3.359083333	10.2608855	Up	8.88E-07
A0A8I3PSS1	SIGMAR1	Sigma non-opioid intracellular receptor 1	3.366256667	10.31203147	Up	3.821E-07
A0A8C0RD50	C18H7orf25	Chromosome 18 C7orf25 homolog	3.368513333	10.32817419	Up	3.718E-06
A0A8C0SHK5	DSG3	Desmoglein-3	3.37423	10.36918069	Up	1.458E-06
A0A8I3Q3C9	WDR6	WD repeat domain 6	3.39652	10.53063114	Up	1.281E-07
A0A8C0TA69	EXOC6	Exocyst complex component	3.40437	10.58808664	Up	1.273E-06
A0A8C0MCG0	PNPLA7	Patatin like phospholipase domain containing 7	3.422463333	10.72171163	Up	5.204E-07
A0A8C0PXU3	DAAM1	Dishevelled associated activator of morphogenesis 1	3.45014	10.9293826	Up	1.24E-06
A0A8I3MV69	MYO10	Myosin X	3.451743333	10.94153567	Up	4.081E-07
A0A8I3S2W5	CDS1	Phosphatidate cytidylyltransferase	3.460363333	11.00710626	Up	2.043E-06
A0A8C0PPG8	-	DNA topoisomerase	3.470456667	11.08438381	Up	1.949E-06
A0A8I3Q8M9	P4HTM	Prolyl 4-hydroxylase, transmembrane	3.483333333	11.18375947	Up	6.711E-08
A0A8I3PP41	RPS6KB1	Ribosomal protein S6 kinase	3.489046667	11.228137	Up	1.237E-06
A0A8C0LW53	-	GH3 domain containing	3.49109	11.24405102	Up	1.456E-06
A0A8C0RWJ1	-	Zinc finger MYND-type containing 11	3.544896667	11.67132676	Up	4.426E-06
A0A8C0SZX7	PRR12	Proline rich 12	3.56591	11.84256758	Up	1.213E-07
A0A8C0Q6H0	DMXL1	Dmx like 1	3.566333333	11.84604308	Up	1.159E-06
A0A8I3PL23	KIF13B	Kinesin-like protein KIF13B	3.604586667	12.16434443	Up	4.84E-08
A0A8C0PHM2	-	Ess-2 splicing factor homolog	3.623733333	12.32685904	Up	6.277E-07
A0A8C0MTG2	RAD18	RING-type E3 ubiquitin transferase	3.64147	12.47934233	Up	3.566E-06
A0A8P0PNW9	WDR76	WD repeat domain 76	3.697413333	12.97275809	Up	1.443E-06
A0A8C0RGH9	-	Bromodomain containing 7	3.70227	13.01650298	Up	1.13E-07
A0A8I3P	GCC1	GRIP and coiled-coil	3.70316	13.0245	Up	2.812

SB1		domain containing 1		3535		E-08
A0A8I3RSG8	L2HGDH	L-2-hydroxyglutarate dehydrogenase	3.706233333	13.05231073	Up	2.69E-05
A0A8C0T3V0	GGT1	Glutathione hydrolase	3.73573	13.32191891	Up	3.266E-06
A0A8C0TJJ8	-	FHA domain-containing protein	3.761216667	13.55935518	Up	2.244E-07
A0A8C0N5U4	ANKIB1	RBR-type E3 ubiquitin transferase	3.767133333	13.61507791	Up	4.805E-09
A0A8P0NDZ8	KIF7	Kinesin family member 7	3.773786667	13.67801211	Up	9.281E-07
A0A8I3PMC1	FASTKD1	FAST kinase domains 1	3.780106667	13.73806268	Up	1.621E-07
A0A8C0MQV2	-	Sulfiredoxin	3.782143333	13.75747053	Up	3.811E-07
A0A8C0SIC4	GDAP2	Ganglioside induced differentiation associated protein 2	3.798653333	13.9158134	Up	6.236E-06
A0A8C0NC30	GMPS	GMP synthase (glutamine-hydrolyzing)	3.798993333	13.91909332	Up	6.285E-07
A0A8I3PMS6	PDE12	Phosphodiesterase 12	3.800733333	13.93589094	Up	1.387E-08
A0A8C0MNP2	-	ATP binding cassette subfamily F member 1	3.81326	14.05742067	Up	1.805E-07
A0A8I3NAF4	BAIAP2L1	BAR/IMD domain containing adaptor protein 2 like 1	3.821736667	14.14025926	Up	1.171E-07
A0A8C0MTY9	CCNB2	Cyclin N-terminal domain-containing protein	3.844443333	14.3645743	Up	6.197E-07
A0A8C0PHH3	TRAPPC10	Trafficking protein particle complex 10	3.8628	14.54851504	Up	3.089E-06
A0A8I3PA68	ING1	Inhibitor of growth protein	3.950276667	15.45794536	Up	2.315E-05
A0A8I3NHW8	ABCA1	ATP binding cassette subfamily A member 1	3.958476667	15.5460555	Up	1.096E-05
A0A8C0R9V3	SMG5	SMG5 nonsense mediated mRNA decay factor	3.96	15.56247916	Up	6.765E-07
A0A8P0SLS9	HDHD2	Haloacid dehalogenase like hydrolase domain containing 2	3.96932	15.66334026	Up	1.247E-06
A0A8C0PRY5	ENO3	phosphopyruvate hydratase	3.972263333	15.69532865	Up	1.062E-06
A0A8P0SEK4	MGA	MAX dimerization protein MGA	3.9746	15.72077025	Up	8.658E-08
A0A8C0SYY0	-	Lipase A, lysosomal acid type	3.978726667	15.76580212	Up	1.17E-07
A0A8P0T5E9	SCAPER	S-phase cyclin A associated protein in the ER	4.000476667	16.00528728	Up	6.707E-07
A0A8C0M7J8	ATP9B	Phospholipid-transporting ATPase	4.0174	16.19414056	Up	6.162E-08
A0A8I3Q234	CCDC77	Coiled-coil domain containing 77	4.02568	16.28735015	Up	8.841E-08
A0A8I3SBB0	PIGQ	Phosphatidylinositol glycan anchor biosynthesis class Q	4.059276667	16.67109158	Up	3.781E-06
A0A8I3NH38	TIMP3	Metalloproteinase inhibitor 3	4.140373333	17.63504477	Up	3.84E-06
A0A8I3PJ	BTF3L4	Transcription factor BTF3	4.14301	17.6673	Up	6.755

G4				0401		E-07
A0A8I3MRK6	YJU2	Splicing factor YJU2	4.155653333	17.82281524	Up	5.534E-06
A0A8I3N494	EXT2	Exostosin glycosyltransferase 2	4.168156667	17.97795062	Up	6.917E-07
A0A8C0RPI2	-	KH RNA binding domain containing, signal transduction associated 3	4.17349	18.04453424	Up	1.581E-06
A0A8P0P0A4	SPDL1	Protein Spindly	4.191056667	18.26559274	Up	1.039E-06
A0A8C0SE28	-	tRNA (adenine(58)-N(1))-methyltransferase catalytic subunit TRMT61A	4.212603333	18.54043687	Up	1.331E-05
A0A8C0SEM1	-	Zinc finger CCCH-type containing, antiviral 1	4.240463333	18.90195212	Up	2.753E-06
A0A8C0PXM0	THNSL1	Threonine synthase like 1	4.251853333	19.05177266	Up	1.262E-06
A0A8I3NS11	CBX2	Chromobox 2	4.257153333	19.12189149	Up	2.733E-06
A0A8C0NFU2	KANSL3	KAT8 regulatory NSL complex subunit 3	4.26732	19.25711931	Up	2.983E-05
A0A8I3PUZ0	MED13	Mediator of RNA polymerase II transcription subunit 13	4.276966667	19.38631464	Up	9.591E-08
A0A8C0T4N0	NES	Nestin	4.30039	19.70363633	Up	5.044E-08
A0A8C0SDV7	CNTRL	Centriolin	4.304143333	19.75496429	Up	3.605E-07
A0A8C0TMQ2	-	Lactamase beta	4.326723333	20.06658672	Up	5.869E-07
A0A8C0QJ67	-	ADP-ribosylation factor	4.357336667	20.49694038	Up	1.179E-07
Q7YR70	AAMP	Angio-associated migratory cell protein	4.36497	20.60567758	Up	2.923E-06
A0A8C0NTP1	-	Rabenosyn, RAB effector	4.371196667	20.69480381	Up	1.966E-07
A0A8I3N386	ALKBH1	AlkB homolog 1, histone H2A dioxygenase	4.390823333	20.97826316	Up	1.945E-06
A0A8C0SDB6	-	RB transcriptional corepressor 1	4.430053333	21.55653411	Up	3.47E-06
A0A8C0PSH5	RBM42	RNA-binding protein 42	4.472406667	22.19875201	Up	2.082E-07
A0A8I3N972	ZFHX3	Zinc finger homeobox 3	4.49275	22.51399229	Up	1.054E-07
A0A8C0SLK9	DAGLB	sn-1-specific diacylglycerol lipase	4.56711	23.7048441	Up	2.828E-05
A0A8I3NGD6	TACC3	Transforming acidic coiled-coil containing protein 3	4.573856667	23.81595782	Up	6.168E-08
A0A8I3RT24	TSEN34	tRNA-splicing endonuclease subunit Sen34	4.623883333	24.6562815	Up	1.349E-06
A0A8I3PIE5	LOC119867740	Tripartite motif-containing protein 26	4.64413	25.00474522	Up	5.112E-07
A0A8C0RUA1	CDCA7L	Cell division cycle associated 7 like	4.66355	25.34360763	Up	9.284E-08
A0A8C0Z2D2	-	Protein POLR1D	4.72968	26.53233975	Up	1.519E-07
Q38QA2	-	Actin (Fragment)	4.77982	27.4706	Up	1.361

				6633		E-09
A0A8C0Z0A5	KDM5C	[histone H3]-trimethyl-L-lysine(4) demethylase	4.819186667	28.23057613	Up	1.235E-06
A0A8I3MZ51	TTYH3	Protein tweety homolog	4.841386667	28.66834401	Up	1.346E-06
A0A8C0T7C5	BRD1	Bromodomain containing 1	4.84737	28.78748801	Up	4.862E-08
A0A8I3S7Q9	APOD	Apolipoprotein D	4.85543	28.94876707	Up	1.699E-07
A0A8I3NLR6	RMC1	Regulator of MON1-CCZ1	4.873	29.30347797	Up	2.196E-06
A0A8I3NU49	BMPR2	receptor protein serine/threonine kinase	4.94365	30.77421177	Up	2.656E-06
A0A8I3MMW1	TASOR2	Transcription activation suppressor family member 2	4.98306	31.62645613	Up	1.919E-06
A0A8C0RDG7	-	RNA helicase	4.98774	31.72921668	Up	4.403E-07
A0A8I3MJM1	S100A2	Protein S100	4.99866	31.97029165	Up	1.447E-06
A0A8P0PDL7	IARS2	isoleucine--tRNA ligase	5.02149	32.48023127	Up	7.796E-08
A0A8I3NCX6	CALCOCO2	Calcium binding and coiled-coil domain 2	5.06137	33.3905974	Up	1.02E-07
A0A8C0RG25	ZNHIT2	Zinc finger HIT-type containing 2	5.06789	33.54184168	Up	6.767E-06
A0A8C0TPY7	PSMD1	26S proteasome non-ATPase regulatory subunit 1	5.120083333	34.77752437	Up	1.566E-07
A0A8I3P3C8	TOP3A	DNA topoisomerase	5.188893333	36.47644771	Up	1.719E-09
A0A8C0P7A6	ATG16L1	Autophagy related 16 like 1	5.363043333	41.15635564	Up	1.844E-06
A0A8C0TP28	VPS37B	VPS37B subunit of ESCRT-I	5.389666667	41.92290156	Up	4.508E-07
A0A8C0NLW6	-	sulfite oxidase	5.40055	42.24035341	Up	1.547E-05
A0A8I3PSA2	HECTD3	HECT domain E3 ubiquitin protein ligase 3	5.453186667	43.80995011	Up	8.463E-07
A0A8C0Q4N0	RAI1	Retinoic acid induced 1	5.57615	47.70769239	Up	1.45E-06
A0A8P0N8M4	CEP290	Centrosomal protein 290	5.583446667	47.94959307	Up	2.311E-06
A0A8I3MXC5	HNRNPK	Heterogeneous nuclear ribonucleoprotein K	5.804876667	55.90388601	Up	2.852E-08
A0A8C0N772	-	Methyltransferase like 7A	5.862716667	58.19069882	Up	1.096E-07
A0A8C0PUE0	-	Family with sequence similarity 120A	5.900973333	59.75441197	Up	2.956E-08
A0A8I3P212	DSP	Desmoplakin	6.537573333	92.89785198	Up	1.224E-08
A0A8C0MX95	DGKE	Diacylglycerol kinase	6.726053333	105.8629046	Up	3.267E-08
A0A8C0YS41	-	Epiplakin 1	7.76534	217.5706286	Up	1.144E-07
