# Supplementary material for: Fascin-1 Promotes Cell Metastasis through Epithelial–Mesenchymal Transition in Canine Mammary Tumor Cell Lines
Source: Vet Sci. 2024 May 25;11(6):238. doi: 10.3390/vetsci11060238 (PMC11209228; doi:10.3390/vetsci11060238)
Supplement: Supplementary file 1 [file vetsci-11-00238-s001.zip › Westernblot_full L/Figure 1.pptx]

## Slide 1
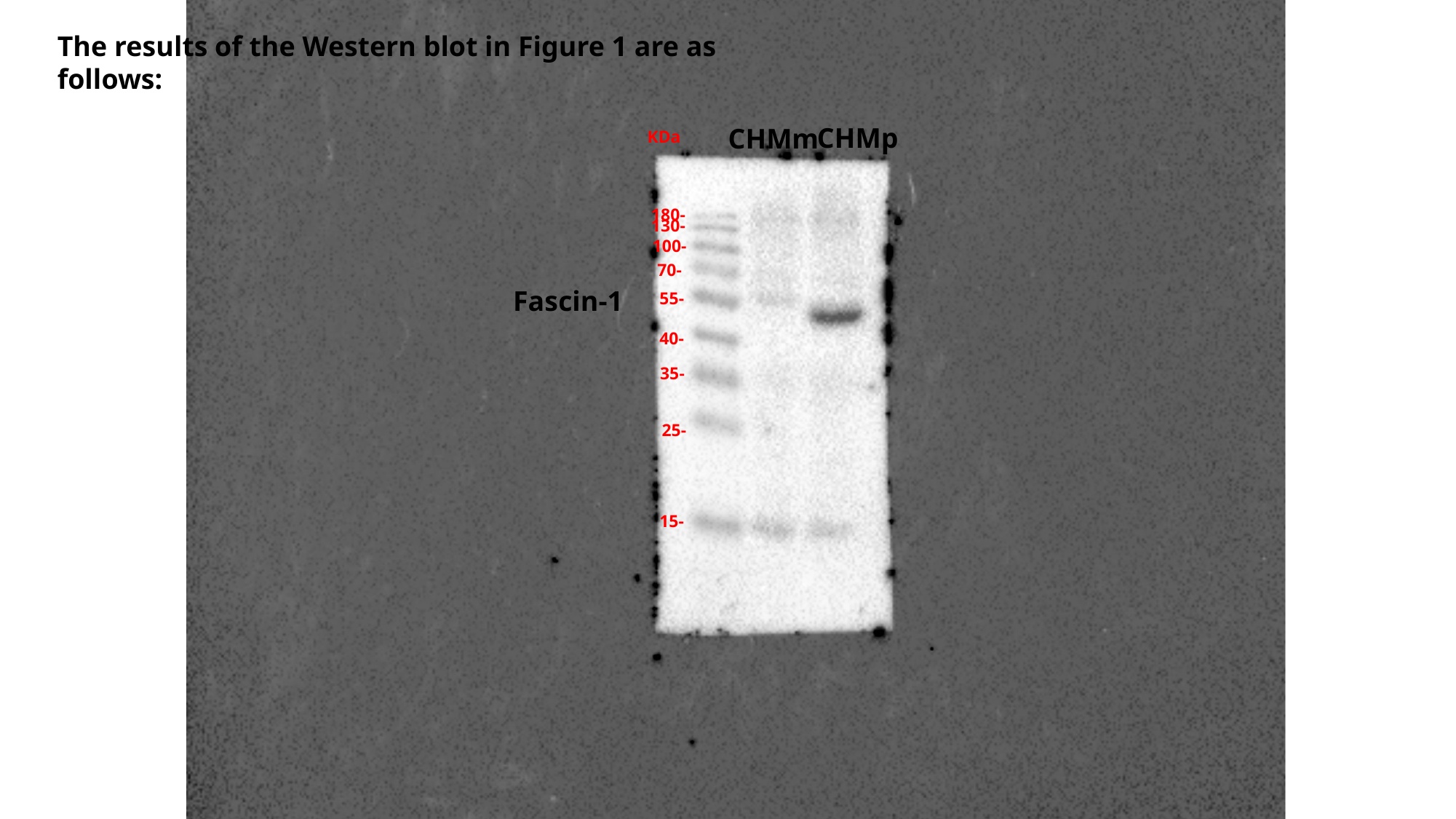

The results of the Western blot in Figure 1 are as follows:
CHMp
CHMm
KDa
180-
130-
100-
70-
Fascin-1
55-
40-
35-
25-
15-

## Slide 2
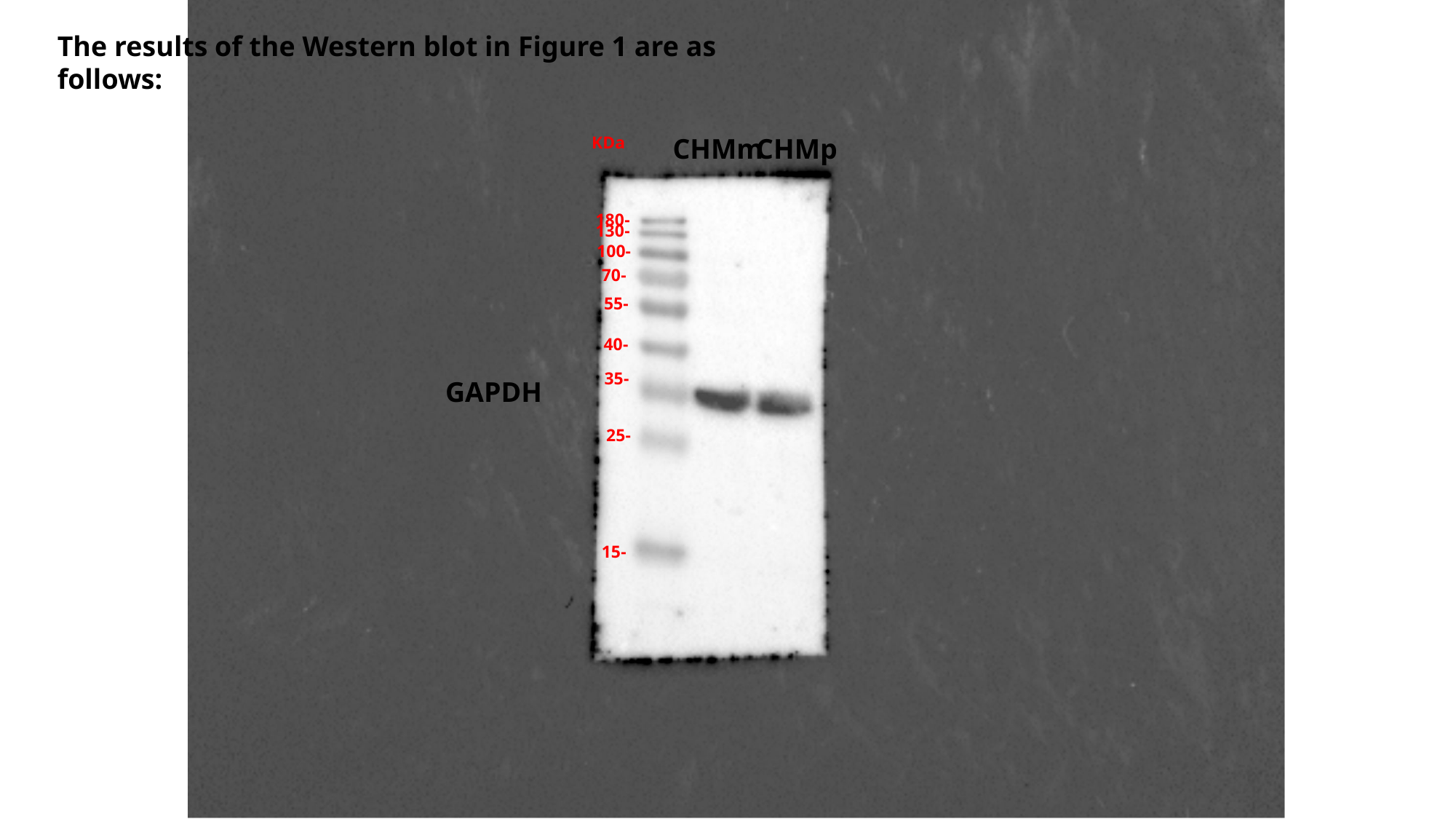

The results of the Western blot in Figure 1 are as follows:
KDa
CHMm
CHMp
180-
130-
100-
70-
55-
40-
35-
GAPDH
25-
15-
