# Supplementary material for: Fascin-1 Promotes Cell Metastasis through Epithelial–Mesenchymal Transition in Canine Mammary Tumor Cell Lines
Source: Vet Sci. 2024 May 25;11(6):238. doi: 10.3390/vetsci11060238 (PMC11209228; doi:10.3390/vetsci11060238)
Supplement: Supplementary file 1 [file vetsci-11-00238-s001.zip › Westernblot_full L/Figure 10.pptx]

## Slide 1
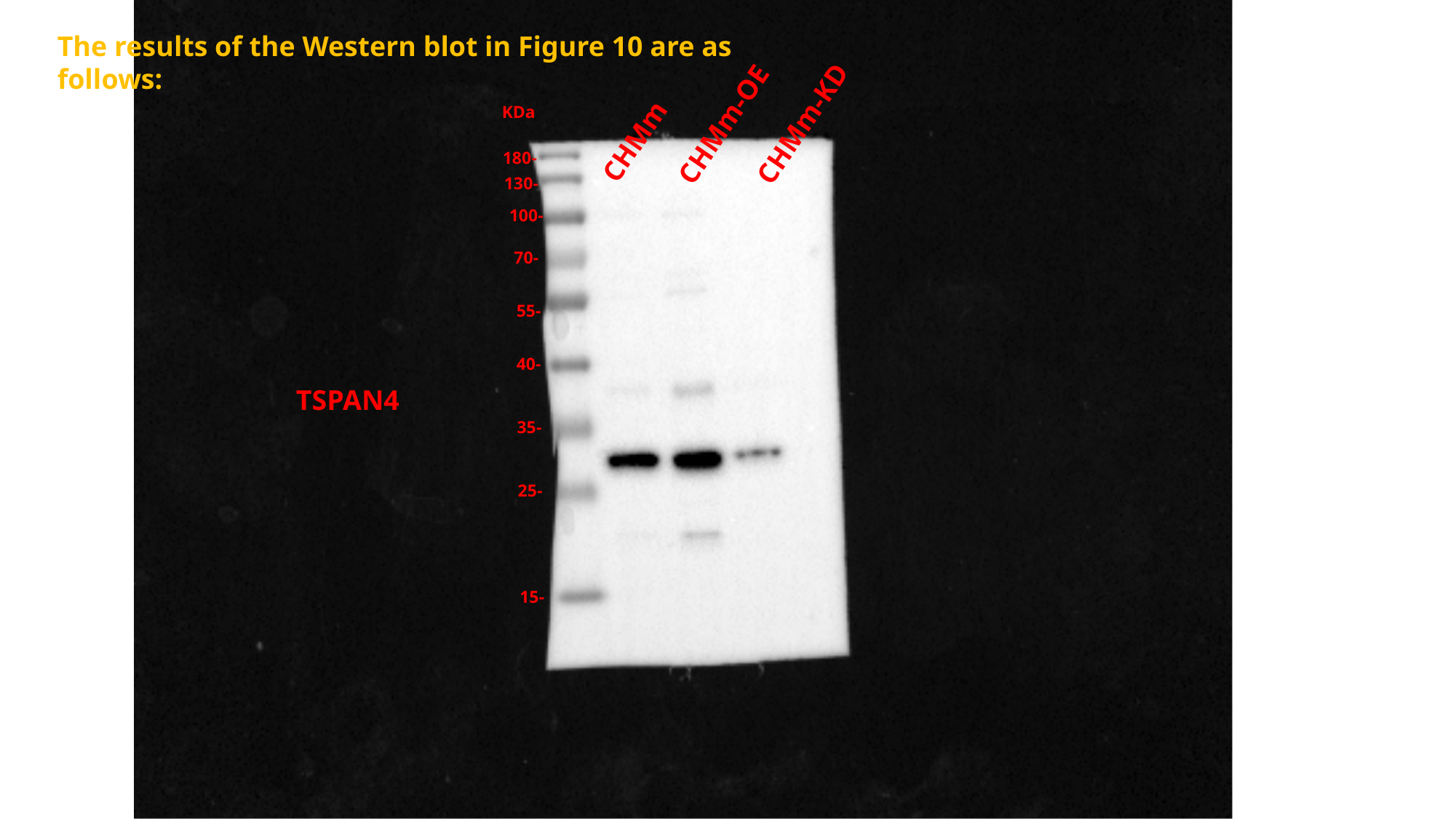

The results of the Western blot in Figure 10 are as follows:
KDa
CHMm-KD
CHMm-OE
CHMm
180-
130-
100-
70-
55-
40-
TSPAN4
35-
25-
15-

## Slide 2
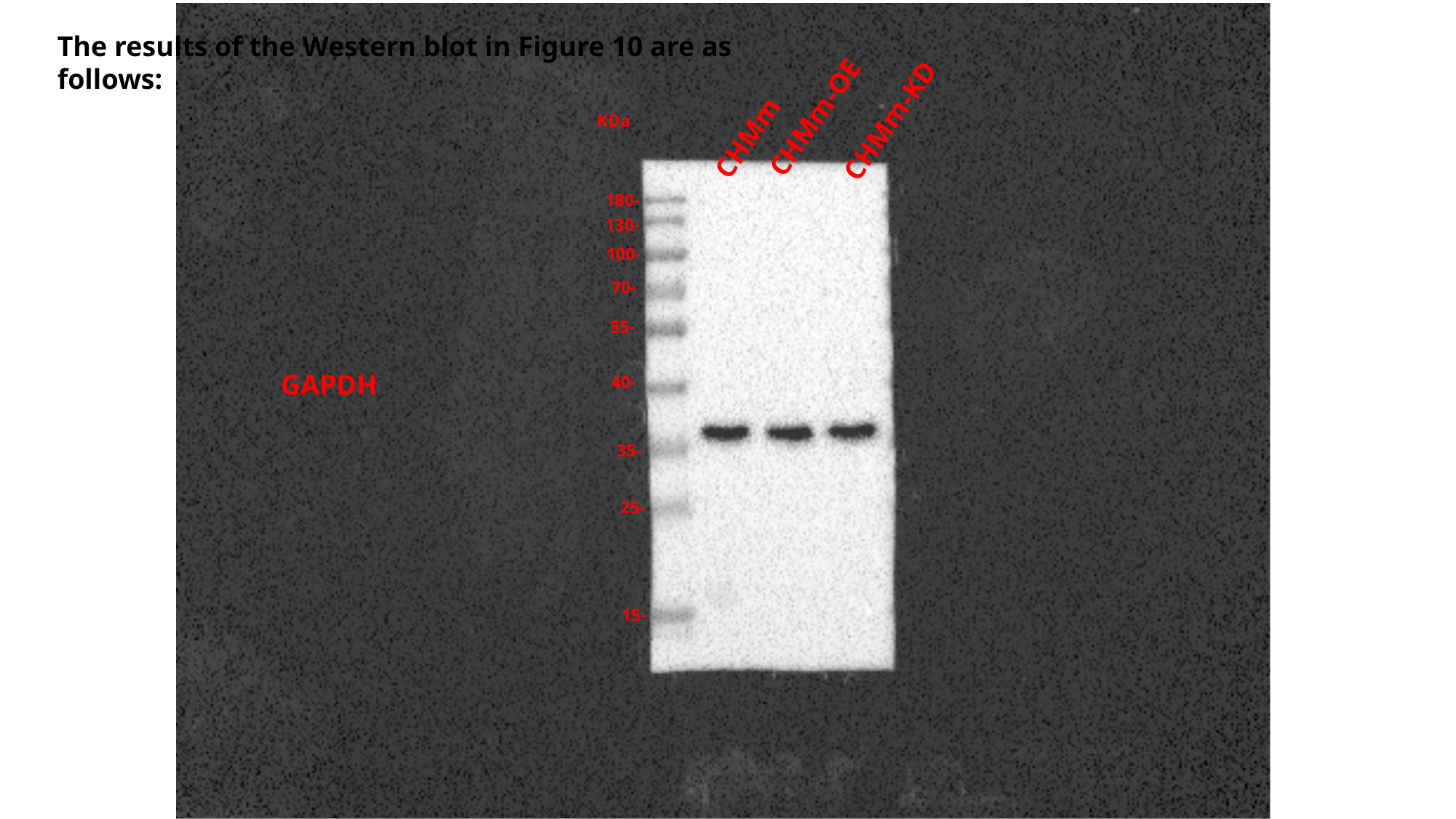

The results of the Western blot in Figure 10 are as follows:
CHMm-OE
CHMm-KD
KDa
CHMm
180-
130-
100-
70-
55-
GAPDH
40-
35-
25-
15-

## Slide 3
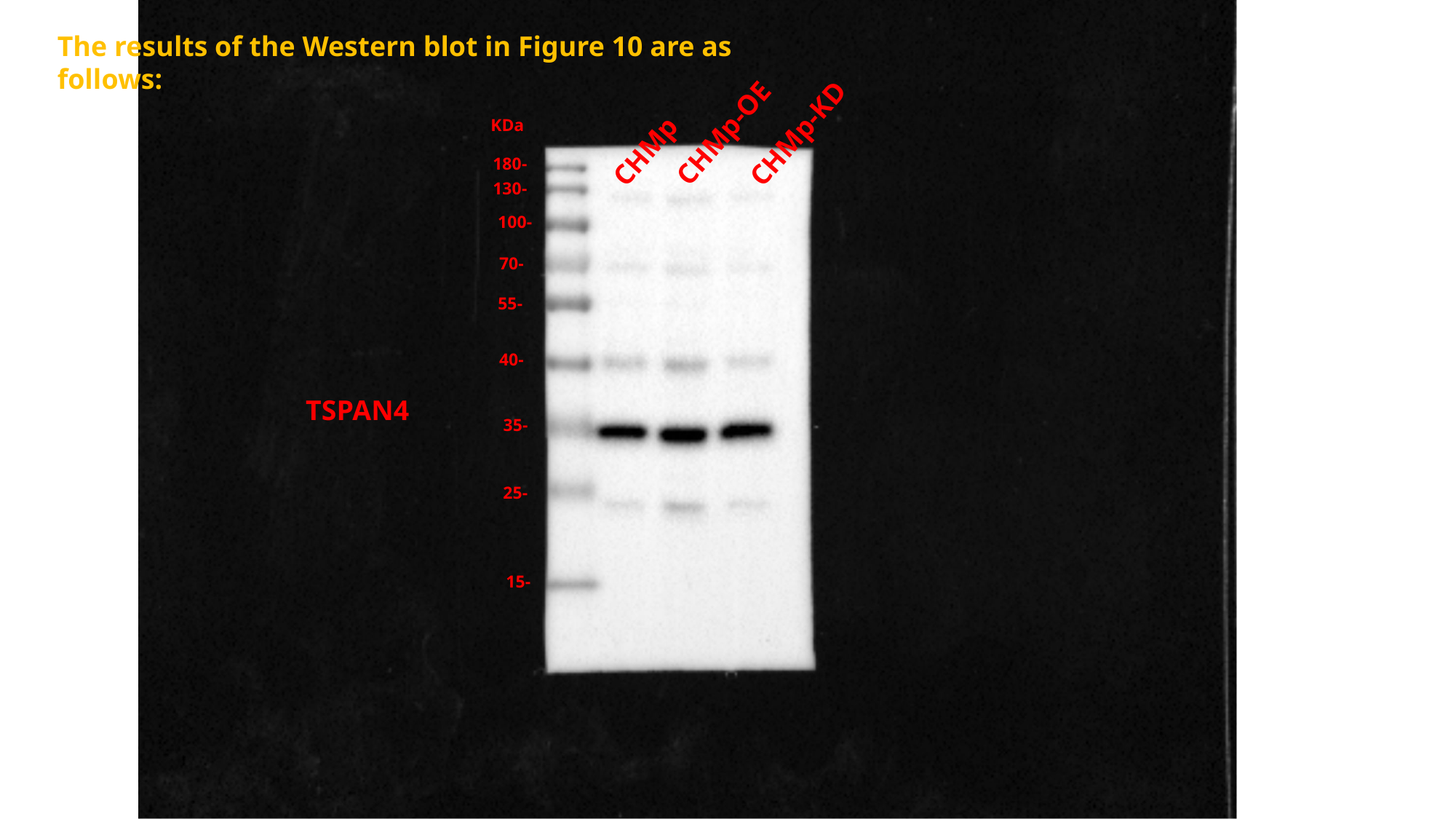

The results of the Western blot in Figure 10 are as follows:
KDa
CHMp-OE
CHMp-KD
CHMp
180-
130-
100-
70-
55-
40-
TSPAN4
35-
25-
15-

## Slide 4
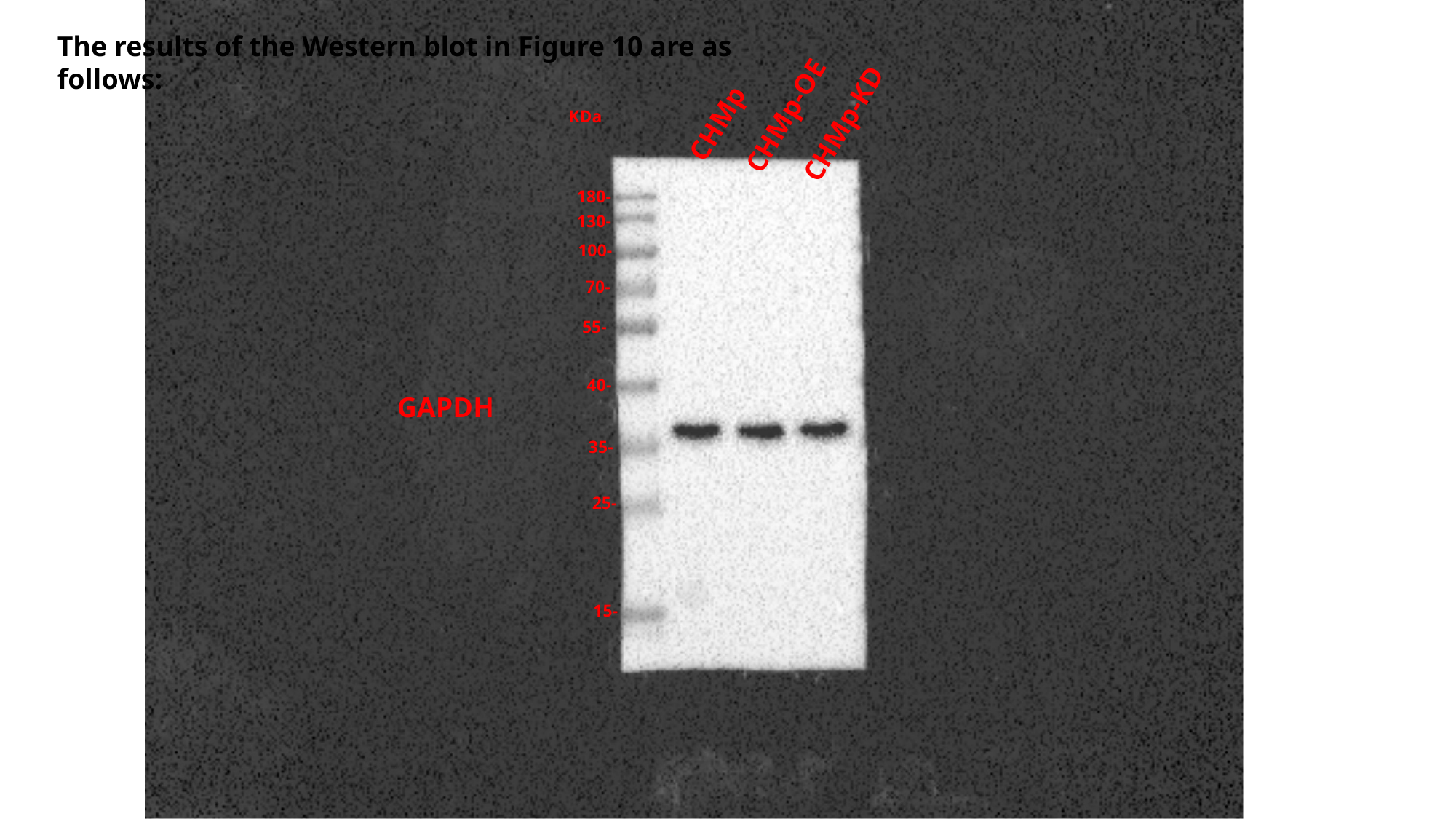

The results of the Western blot in Figure 10 are as follows:
CHMp-OE
KDa
CHMp
CHMp-KD
180-
130-
100-
70-
55-
40-
GAPDH
35-
25-
15-
