# Supplementary material for: Fascin-1 Promotes Cell Metastasis through Epithelial–Mesenchymal Transition in Canine Mammary Tumor Cell Lines
Source: Vet Sci. 2024 May 25;11(6):238. doi: 10.3390/vetsci11060238 (PMC11209228; doi:10.3390/vetsci11060238)
Supplement: Supplementary file 1 [file vetsci-11-00238-s001.zip › Westernblot_full L/Figure 2.pptx]

## Slide 1
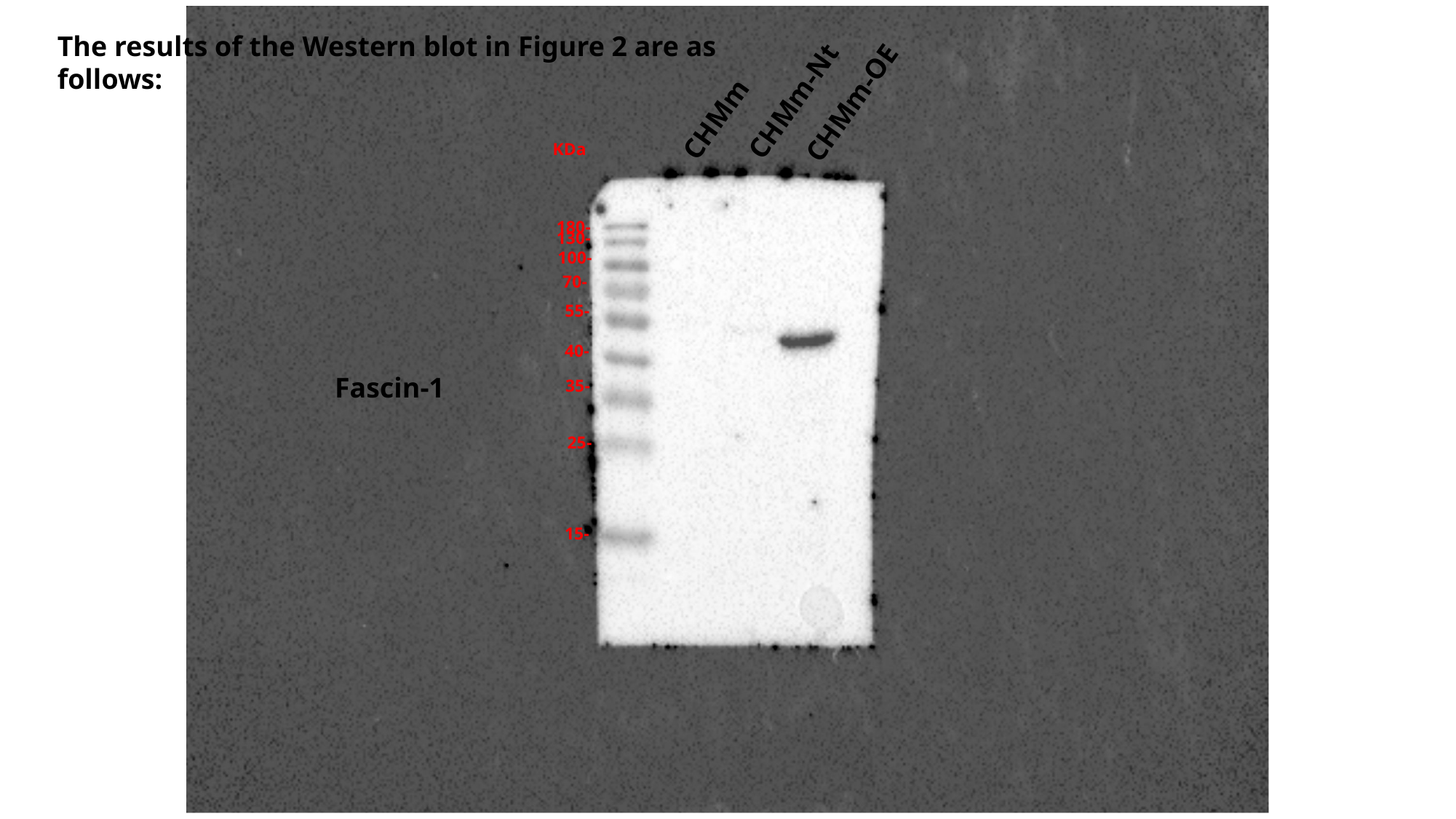

The results of the Western blot in Figure 2 are as follows:
CHMm-Nt
CHMm-OE
CHMm
KDa
180-
130-
100-
70-
55-
40-
Fascin-1
35-
25-
15-

## Slide 2
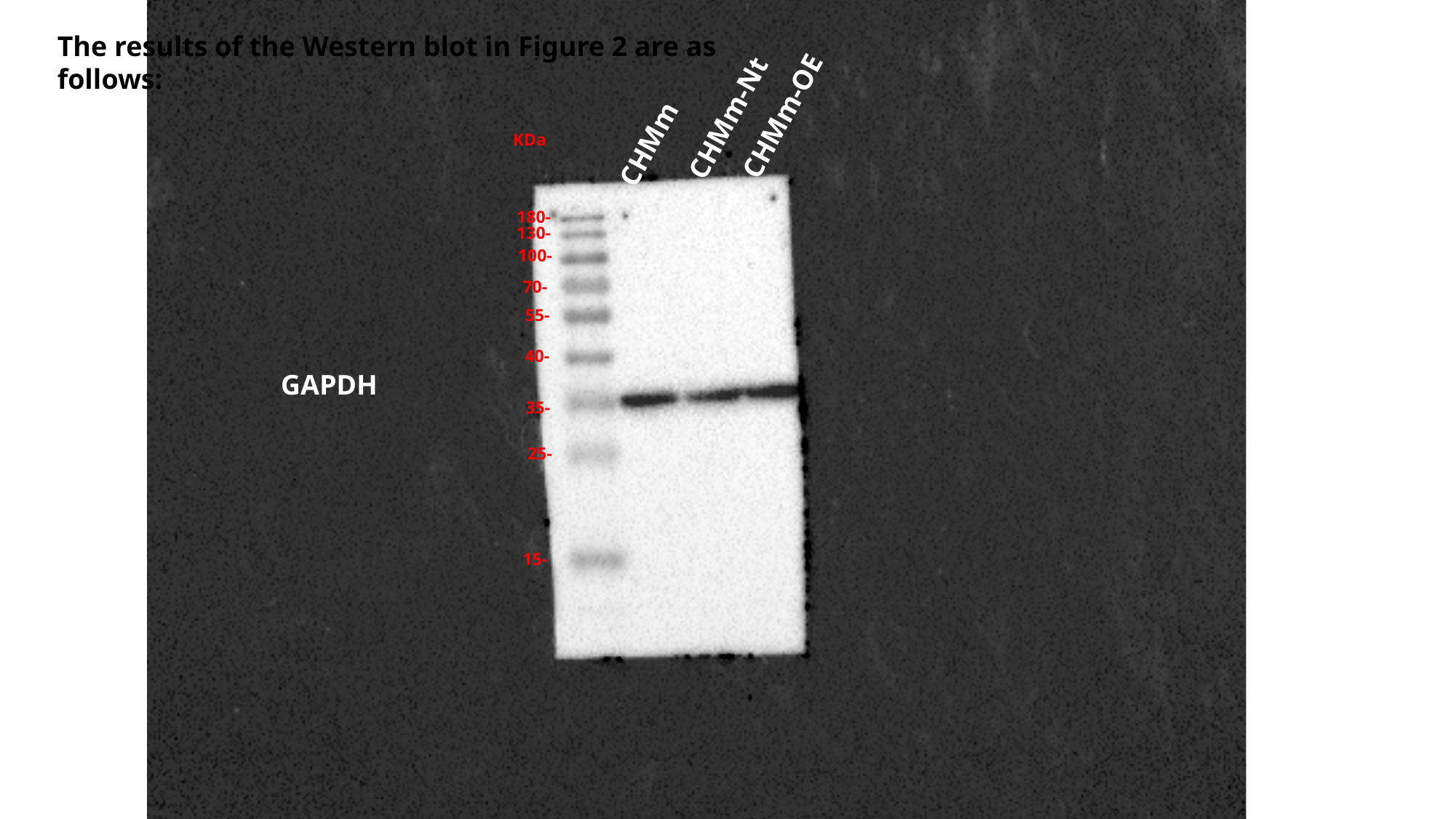

The results of the Western blot in Figure 2 are as follows:
CHMm-OE
CHMm-Nt
CHMm
KDa
180-
130-
100-
70-
55-
40-
GAPDH
35-
25-
15-

## Slide 3
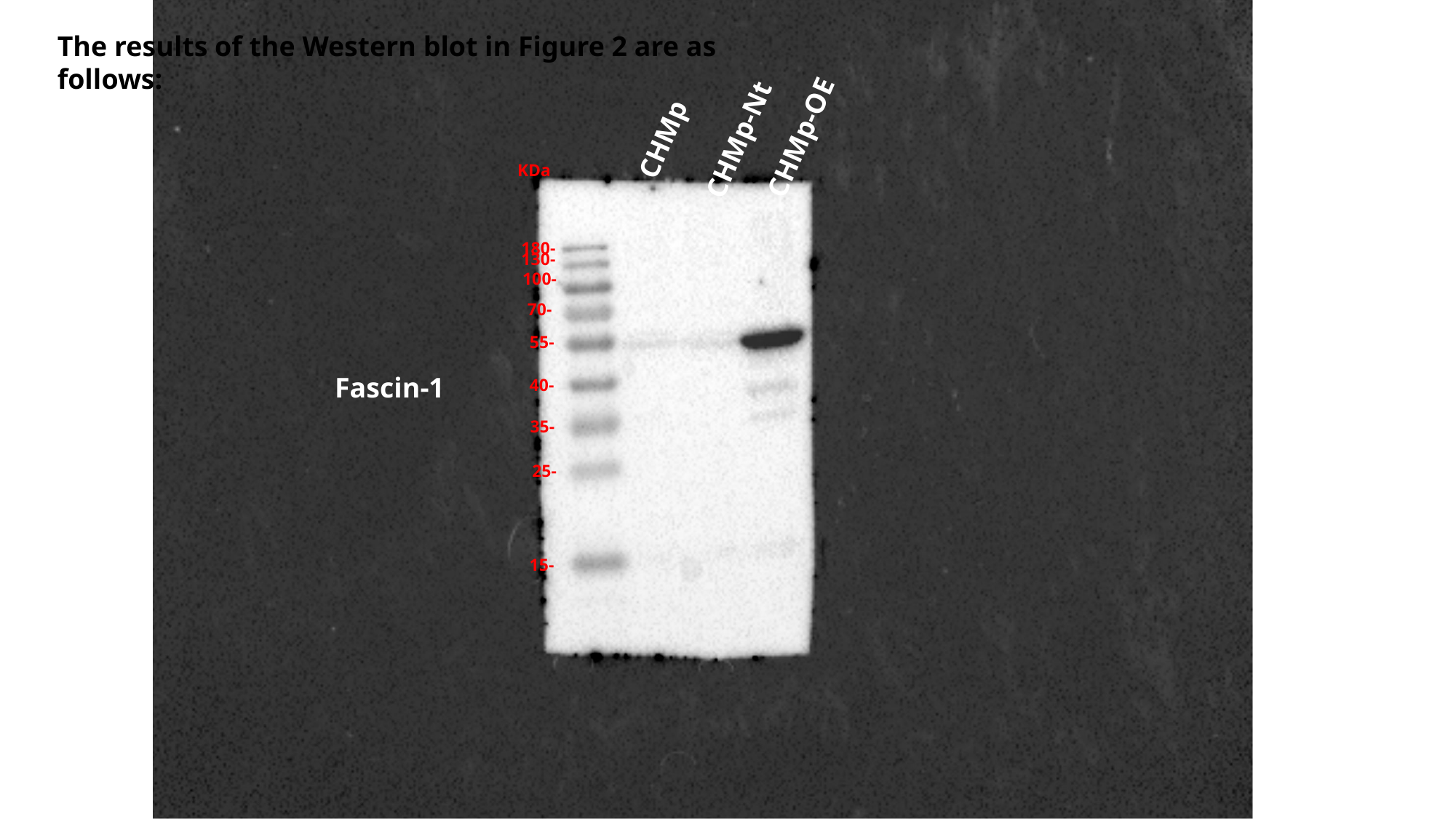

The results of the Western blot in Figure 2 are as follows:
CHMp-OE
CHMp
CHMp-Nt
KDa
180-
130-
100-
70-
55-
Fascin-1
40-
35-
25-
15-

## Slide 4
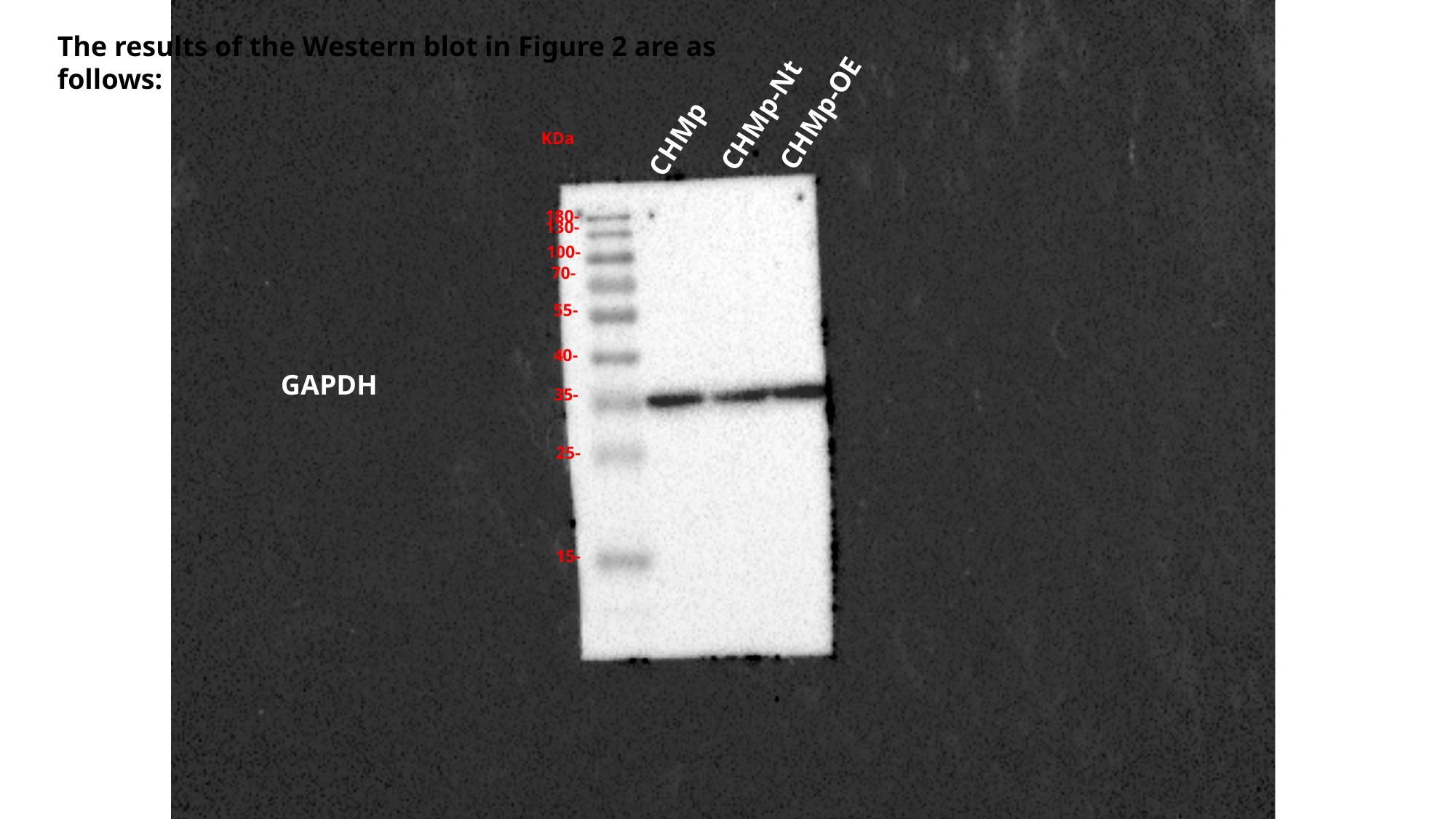

The results of the Western blot in Figure 2 are as follows:
CHMp-OE
CHMp-Nt
CHMp
KDa
180-
130-
100-
70-
55-
40-
GAPDH
35-
25-
15-

## Slide 5
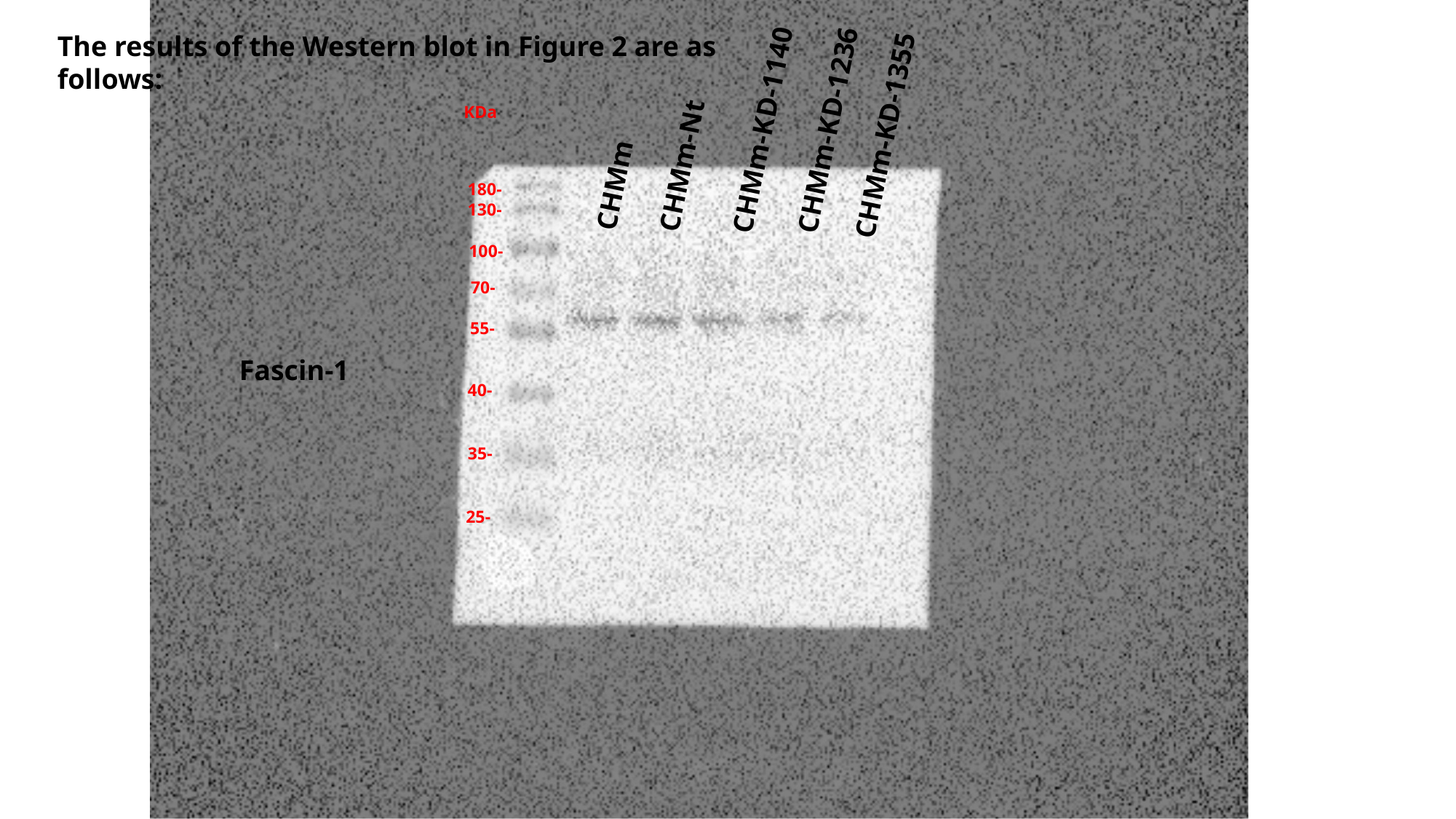

The results of the Western blot in Figure 2 are as follows:
KDa
CHMm-KD-1140
CHMm-KD-1236
CHMm-KD-1355
CHMm-Nt
CHMm
180-
130-
100-
70-
55-
Fascin-1
40-
35-
25-

## Slide 6
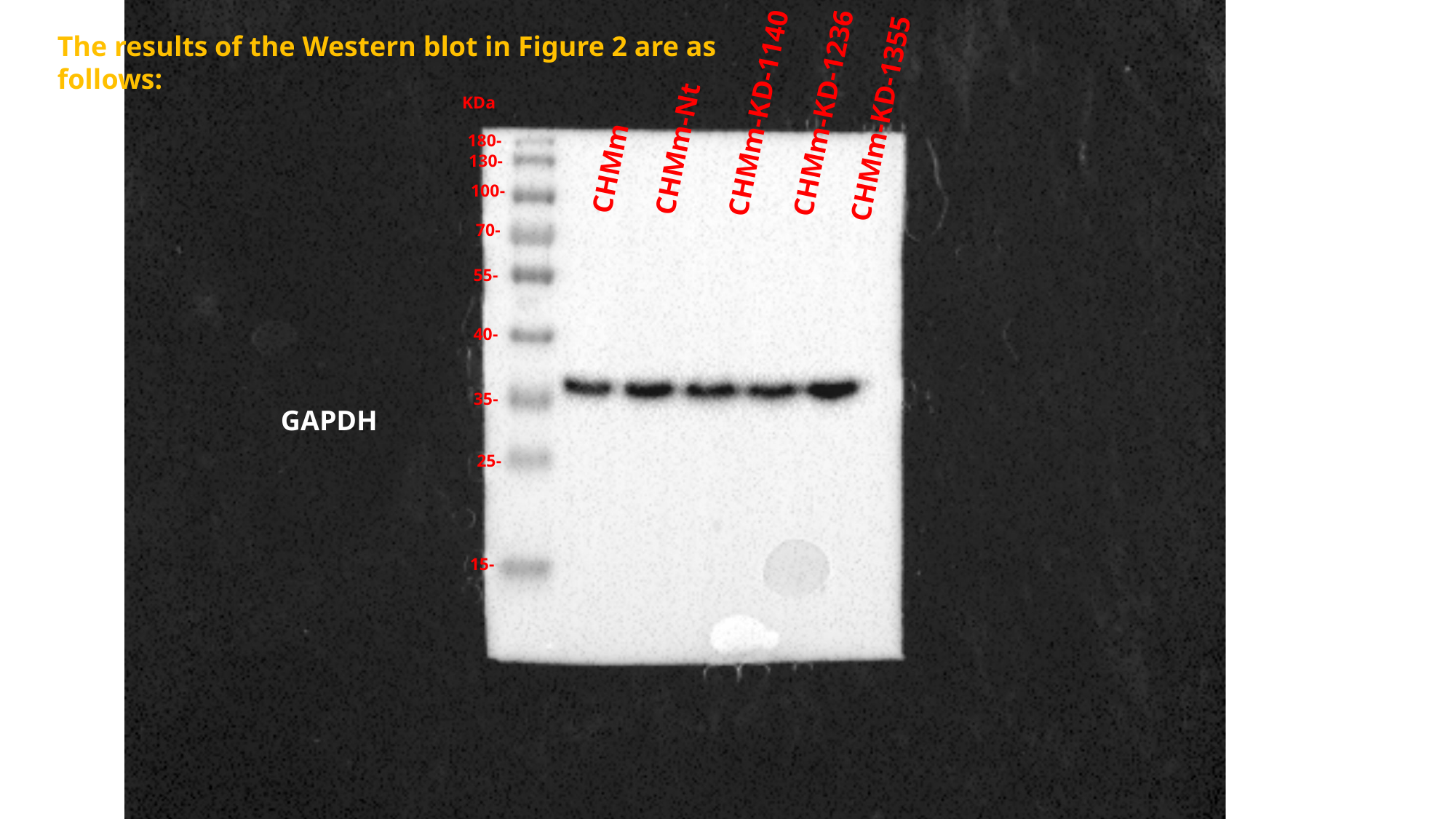

The results of the Western blot in Figure 2 are as follows:
KDa
CHMm-KD-1140
CHMm-KD-1236
CHMm-KD-1355
180-
CHMm-Nt
130-
CHMm
100-
70-
55-
40-
35-
GAPDH
25-
15-

## Slide 7
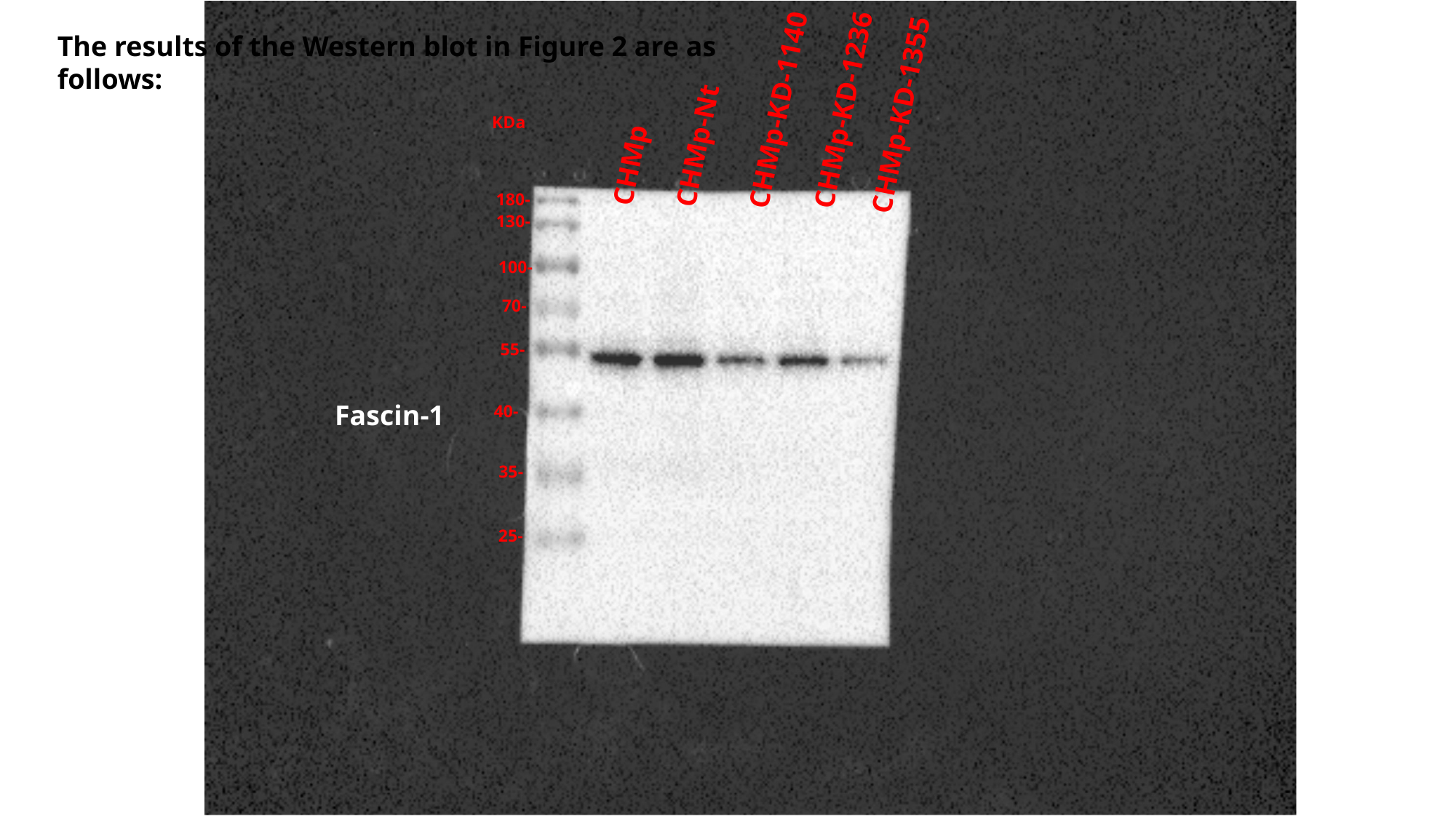

The results of the Western blot in Figure 2 are as follows:
CHMp-KD-1140
CHMp-KD-1236
CHMp-KD-1355
KDa
CHMp-Nt
CHMp
180-
130-
100-
70-
55-
Fascin-1
40-
35-
25-

## Slide 8
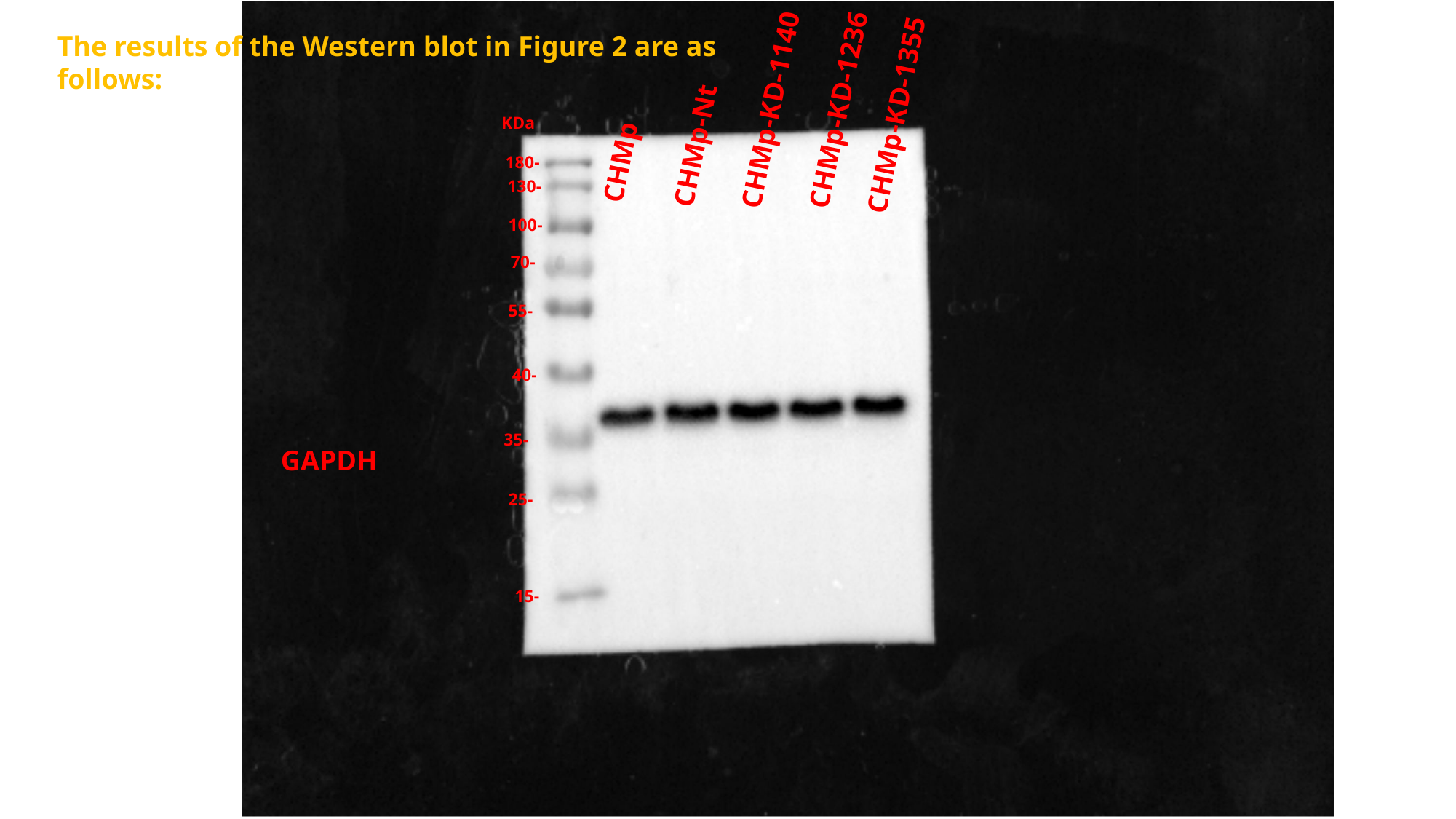

The results of the Western blot in Figure 2 are as follows:
CHMp-KD-1140
CHMp-KD-1236
CHMp-KD-1355
KDa
CHMp-Nt
CHMp
180-
130-
100-
70-
55-
40-
35-
GAPDH
25-
15-
