# Supplementary material for: Fascin-1 Promotes Cell Metastasis through Epithelial–Mesenchymal Transition in Canine Mammary Tumor Cell Lines
Source: Vet Sci. 2024 May 25;11(6):238. doi: 10.3390/vetsci11060238 (PMC11209228; doi:10.3390/vetsci11060238)
Supplement: Supplementary file 1 [file vetsci-11-00238-s001.zip › Westernblot_full L/Figure 8.pptx]

## Slide 1
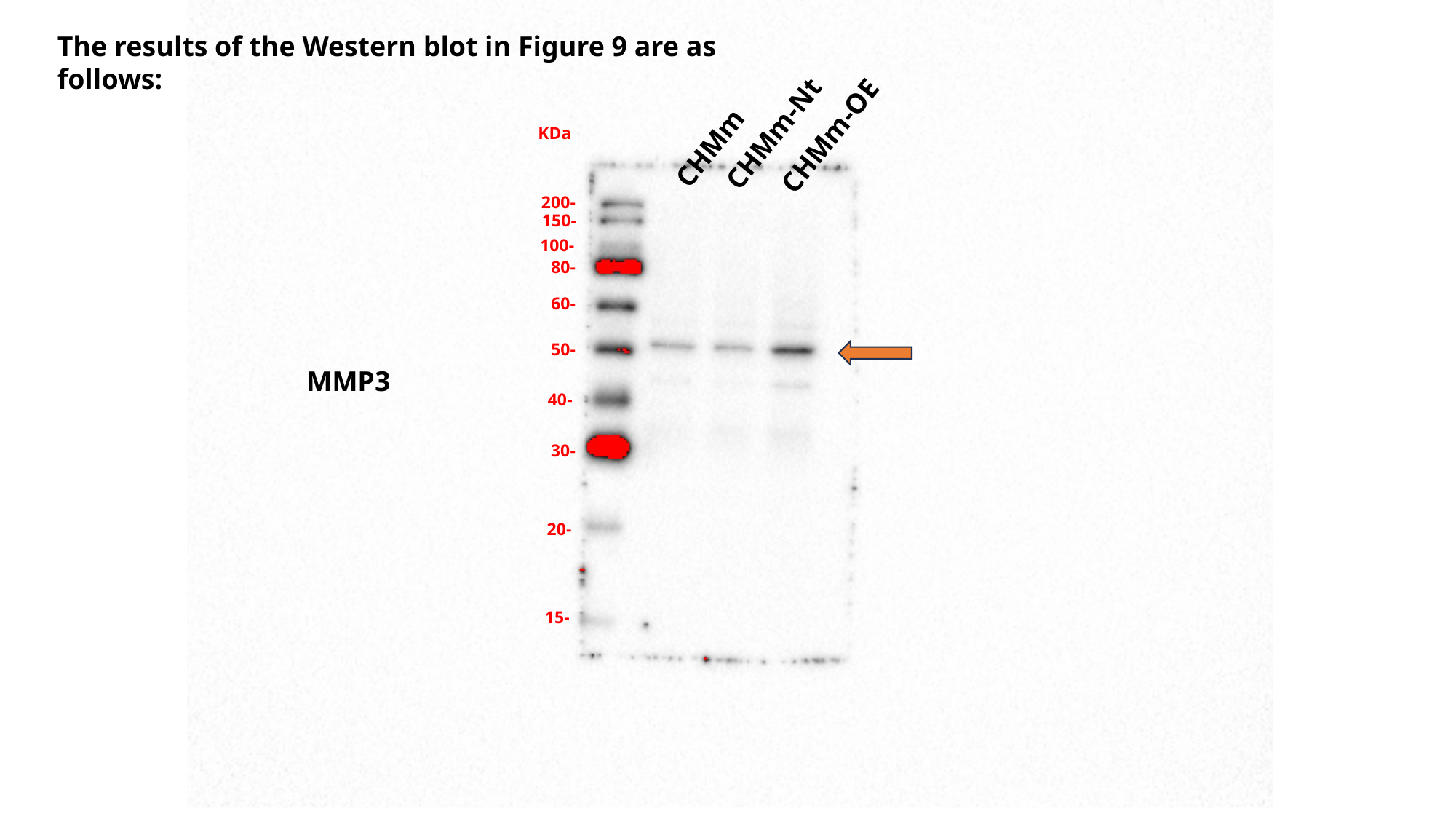

The results of the Western blot in Figure 9 are as follows:
CHMm-Nt
CHMm-OE
KDa
CHMm
200-
150-
100-
80-
60-
50-
MMP3
40-
30-
20-
15-

## Slide 2
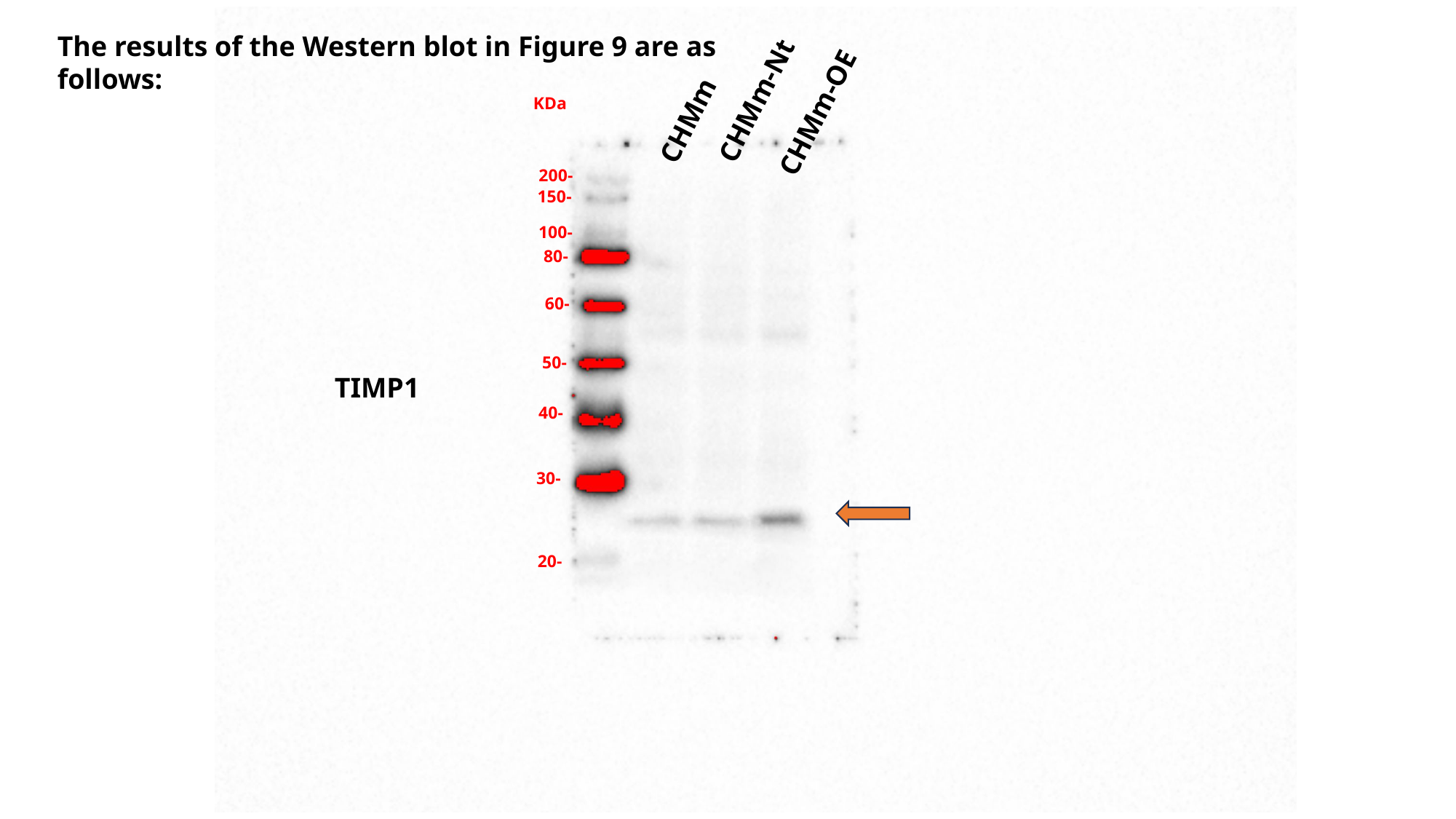

The results of the Western blot in Figure 9 are as follows:
CHMm-Nt
KDa
CHMm-OE
CHMm
200-
150-
100-
80-
60-
50-
TIMP1
40-
30-
20-

## Slide 3
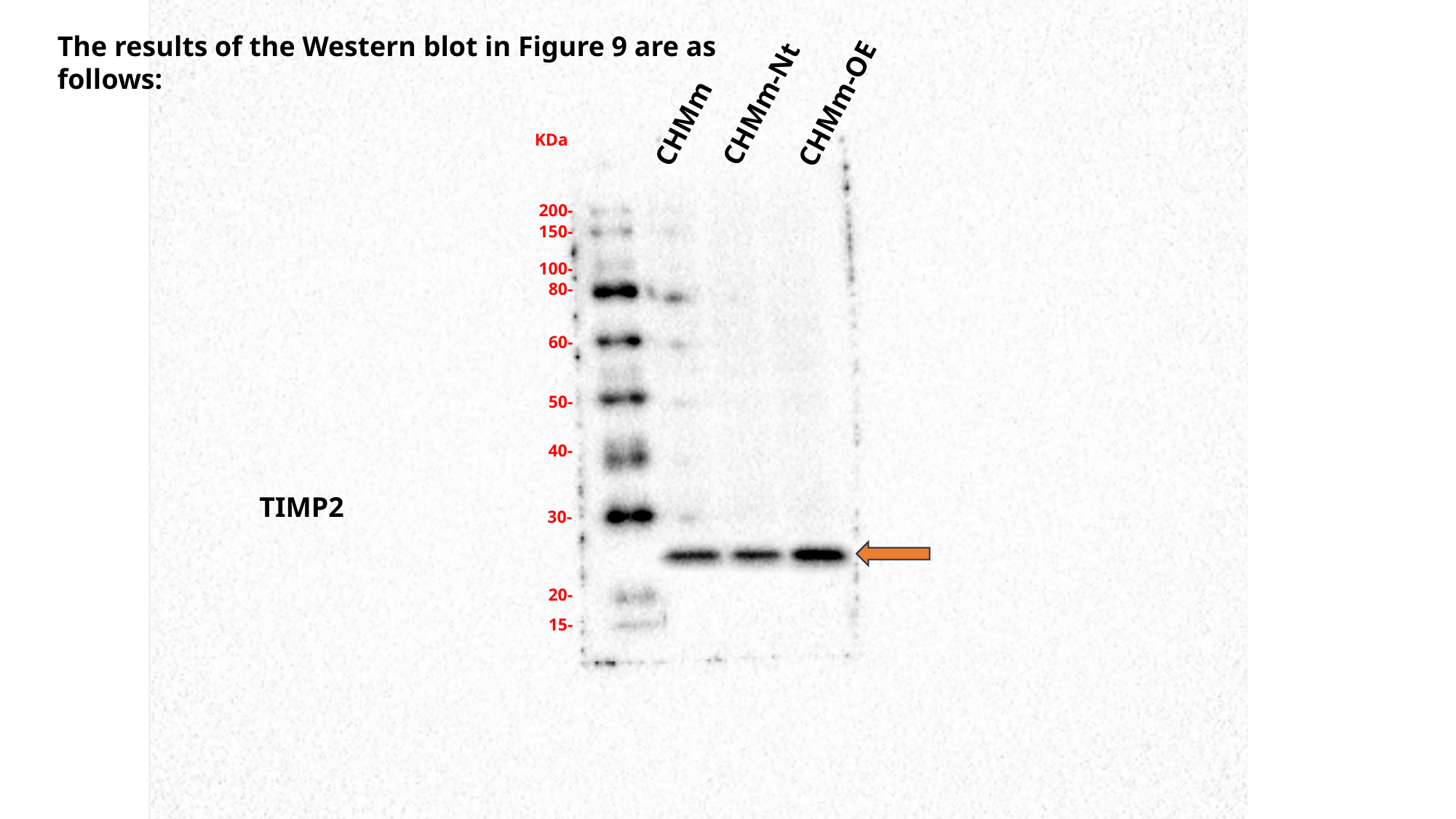

The results of the Western blot in Figure 9 are as follows:
CHMm-OE
CHMm-Nt
CHMm
KDa
200-
150-
100-
80-
60-
50-
40-
TIMP2
30-
20-
15-

## Slide 4
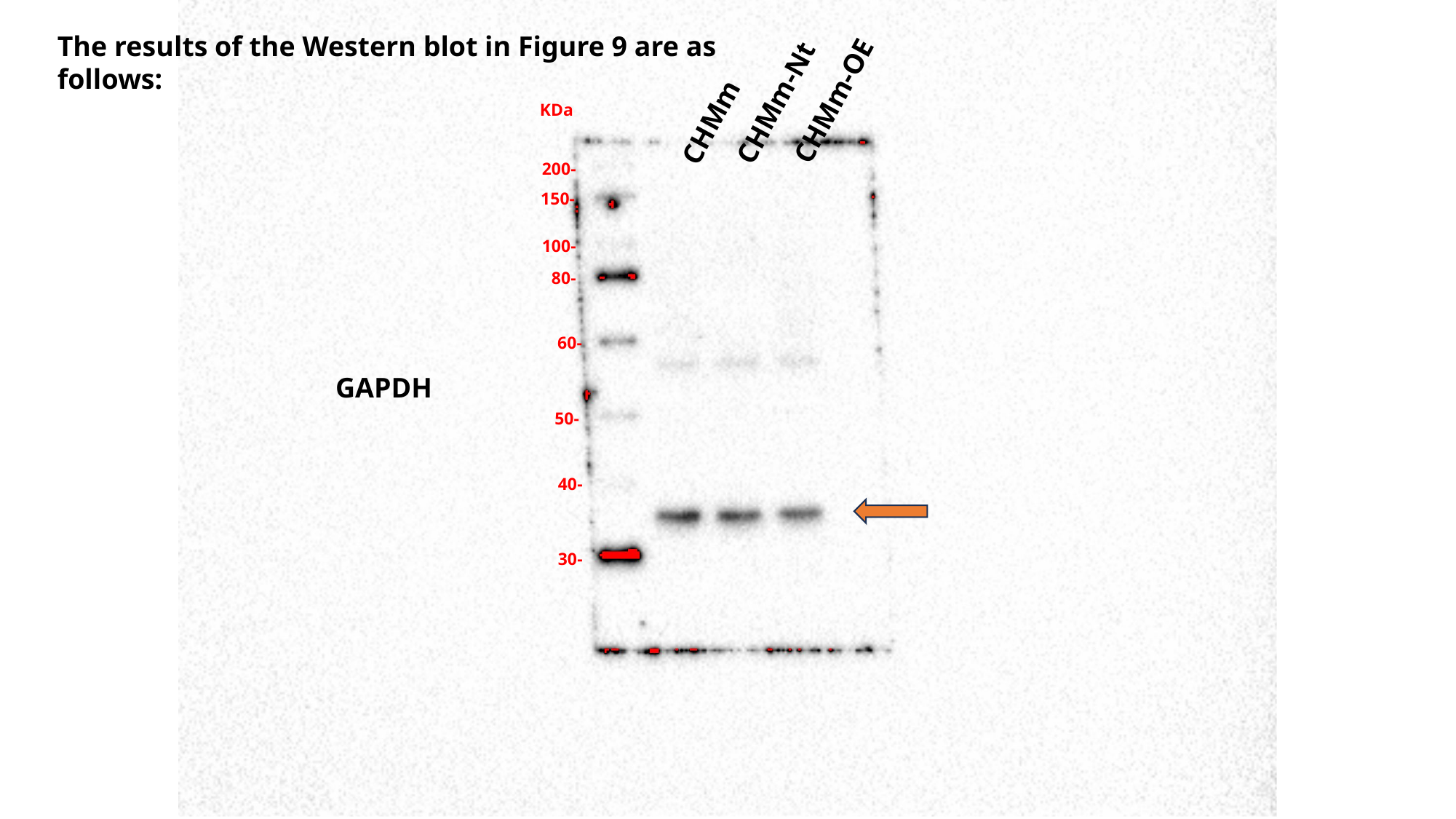

The results of the Western blot in Figure 9 are as follows:
CHMm-OE
CHMm-Nt
KDa
CHMm
200-
150-
100-
80-
60-
GAPDH
50-
40-
30-
